# A four-phase framework for culturally responsive vaccine messaging

**DOI:** 10.1080/21642850.2026.2666714

**Published:** 2026-05-19

**Authors:** Kyra Hamilton, Jessica Balla, Amber Carmen Arroyo, Mayra Y. Bámaca, Anna E. Epperson, Rosa D. Manzo, Mercedes Peña, Martin S. Hagger

**Affiliations:** a School of Applied Psychology, Griffith University, Brisbane, Australia; b Faculty of Sport and Health Sciences, University of Jyväskylä, Jyväskylä, Finland; c Health Sciences Research Institute, University of California, Merced, U.S; d Department of Psychological Sciences, University of California, Merced, U.S; e Department of Medical Education, University of California, Merced, U.S

**Keywords:** Vaccination, mixed-methods, design science, intervention, behavior change

## Abstract

**Background:**

Efficacious messaging campaigns are essential for increasing vaccine uptake, particularly in communities with low coverage and heightened hesitancy. A major barrier to equitable vaccination is the lack of culturally responsive messaging that reflects the values and experiences of underserved minoritized populations. Despite growing recognition of cultural responsiveness, few studies offer systematic, theory-informed approaches to message design, implementation, and validation. To address this gap, we propose a four-phase framework that explicitly centers culture and behavioral theory into vaccine messages, with the goal of improving relevance, resonance, and effectiveness among marginalized groups.

**Methods:**

Grounded in a design science approach, the framework comprises four integrated phases across seven sequential steps. *Phase 1* involves formative research comprising an environmental scan of public health messaging (Step 1) and a systematic review of message efficacy studies (Step 2), as well as focus groups assessing the cultural responsiveness of existing messages (Step 3). *Phase 2* includes belief elicitation through open-ended surveys to identify key factors influencing vaccine decisions (Step 4). *Phase 3* uses quantitative methods to examine belief-based correlates of vaccination hesitancy and intentions (Step 5). *Phase 4* applies findings from earlier phases to develop culturally informed message prototypes, assessed for appropriateness through focus groups (Step 6) and evaluated in a feasibility trial using a pre-post design (Step 7).

**Conclusion:**

This four-phase framework offers a rigorous, replicable approach for developing theory-driven, culturally tailored health messages. It is intended for future vaccine communication initiatives and can be adaptable to other health behavior contexts and diverse minoritized populations.

## Introduction

The development of culturally responsive messages aimed at changing behaviour is one promising way to improve health equity and enhance the efficacy of public health interventions among diverse minoritized populations (Torres-Ruiz et al., 2018). Yet, despite growing recognition of the need for culturally tailored health communication, there remains a notable paucity of systematic, theory-driven frameworks to guide the design, implementation, and evaluation of behaviour change messages specifically for marginalised communities (Lapinski et al., 2025). Conventional approaches to health messaging often rest on generalised assumptions that neglect the nuanced sociocultural factors shaping health beliefs, behaviours, and decision-making processes (Noar et al., 2009). As a result, these approaches risk reinforcing disparities by failing to engage meaningfully with the lived experiences, linguistic preferences, and historical contexts of diverse minoritized populations, particularly those marked by structural disadvantage and medical mistrust (Bogart et al., 2021; Halpern-Felsher & McLaughlin, 2018).

In contrast, culturally responsive messaging needs to account for and be informed by the community-specific values, norms, and communication practices of the target audience. This consideration of contextual and audience characteristics and needs enhances message salience, credibility, and behavioural resonance (Lapinski et al., 2025). By incorporating cultural relevance into the design of health messages, interventionalists can promote more equitable access to health information, foster trust and agency, and encourage active engagement among historically underserved groups (Barrera et al., 2017; Lapinski et al., 2025; Peprah et al., 2023). Indeed, contemporary health communication research has increasingly begun to utilise community involvement in health information design, co-construction processes, and contextual tailoring in vaccine and health promotion contexts (Kemp et al., 2022; Langford, 2020; Parsa et al., 2025; Upshaw et al., 2024), illustrating their practicality and effectiveness in applied settings. Thus, cultural responsiveness should be regarded not merely as a gesture of inclusivity but as a fundamental determinant of the efficacy and effectiveness, reach, and sustainability of health behaviour change strategies (Barrera et al., 2017). Given this imperative, there is a pressing need for robust, integrative frameworks that encompass behavioural science as the preeminent means of providing a conceptual basis for individuals’ behaviour, alongside considerations of cultural relevance and norms (Arana-Chicas et al., 2025; World Health Organisation [WHO], 2022). Such frameworks can support the development of interventions that have a sound conceptual basis, are empirically grounded, and are informed by relevant cultural factors, which will ultimately improve message efficacy and increase the likelihood of adaptive health outcomes and mitigation of persistent disparities observed in marginalised populations (Lapinski et al., 2025).

Given the critical need for culturally responsive, theory-informed communication strategies in behavioural interventions, research has shed light on the psychological, social, and socio-structural factors that promote engagement in preventive health behaviours (Hagger & Hamilton, 2022b; Hagger et al., 2020), including vaccination (Hagger & Hamilton, 2022a; Hamilton & Hagger, 2022). The efficacy of messaging campaigns in motivating vaccination uptake is therefore contingent on the extent to which the target can activate or change these determinants, particularly those that are modifiable, through carefully crafted content (Labbé et al., 2025; Liu et al., 2024; Malik et al., 2023; Nyhan et al., 2014). Notably, ‘bottom-up’ approaches, which involve co-design processes with target communities, have demonstrated superior outcomes in changing health behaviour compared to more traditional ‘top-down’ strategies driven by government agencies or academic researchers (Marathe et al., 2011; Wallerstein & Duran, 2006). In parallel, messaging interventions grounded in behavioural theory consistently outperform those that are merely ‘theory-inspired’ or entirely atheoretical, in promoting behaviour change (Hagger & Weed, 2019). Despite these insights, there remains a marked lack of research employing community-engaged, contextually tailored strategies with behavioural science methods to develop theory-based vaccination messages aimed at underserved populations. This gap is of particular concern in light of the urgent need for effective communication strategies in communities that experience systemic barriers to healthcare access and demonstrate persistently low vaccination uptake and high vaccination hesitancy (Arana-Chicas et al., 2025; Liu et al., 2024). Bridging this gap necessitates a deliberate emphasis on participatory, culturally responsive approaches to intervention design that integrate theoretical rigour with community-specific relevance, thereby advancing efforts to promote health equity and reduce disparities in preventive care (Barrera et al., 2017; Wallerstein & Duran, 2006).

Addressing these persistent gaps requires moving beyond ad hoc or generalised communication efforts toward structured, evidence-based strategies that are both culturally responsive and theoretically grounded. Doing so necessitates the development of systematic protocols to develop culturally responsive vaccine messaging that employ an integrative framework that simultaneously encompasses theoretical insight from behavioural science and procedures to ensure cultural relevance and community engagement. Such an approach should pervade each developmental step of message development, including the inception, design, implementation, and evaluation of the messages across multiple phases. To this end, we propose a four-phase approach that offers a structured methodology purposed for use during early-stage message development to ensure cultural considerations are embedded at every stage of the messaging intervention process. While some points of convergence situate this four-phase framework within the broader intellectual tradition of community-engaged, co-constructed, and culturally-grounded methods to understand and communicate about health behaviours, such as the PEN-3 model and Dutta’s Culture-Centred Approach (Airhihenbuwa, 1989; Dutta, 2008), the distinct procedural contribution of this framework lies in its integration of knowledge from theory-based behavioural science disciplines with culture-centred approaches towards the aim of optimising message effectiveness in influencing prominent health behaviour determinants while ensuring accessibility, resonance, and appropriateness of messages to the target community and population.

Specifically, phase one centres on formative research, including environmental scans, literature reviews, and cultural assessments, to establish a foundational evidence base regarding existing vaccine messaging practices, their effectiveness, and their degree of cultural responsiveness. In phase two, elicitation methods are employed to identify salient beliefs and perceptions related to vaccination within the target populations. Phase three utilises theoretical and quantitative approaches to isolate key belief constructs and determinants of vaccine-related behaviours specific to communities from minoritized backgrounds. Finally, phase four applies these insights to inform the development, refinement, and empirical validation of culturally tailored message prototypes through iterative testing and feasibility assessments. By anchoring the protocol in both behavioural science and cultural competence, this framework supports the creation of vaccine messages for use in communication-based interventions that are empirically grounded and contextually meaningful. Such an approach is designed to enhance vaccine uptake in populations from culturally diverse communities, particularly those that are minoritized and underserved in healthcare and public health, and, in doing so, address entrenched health disparities through systematically developed communication interventions that are culturally congruent and rigorously evaluated. Moreover, this protocol is anticipated to serve as a valuable template for future researchers aiming to develop and assess culturally appropriate messaging not only across diverse vaccine-preventive contexts and populations but also within broader health behaviour domains. The following section outlines the four-phase framework, along with its objectives and corresponding component steps.

### A four-phase framework for culturally responsive messaging

#### Phase 1. formative research

The objective of Phase 1 is to establish foundational knowledge on the content, purpose, target outcomes, framing, method of delivery, and target audience of existing vaccination messaging practices and their perceived efficacy and effectiveness, with particular attention to cultural responsiveness. This phase comprises three interrelated steps. Step 1 involves conducting an environmental scan of vaccination messages disseminated by authorised health authorities, including government agencies and public health organisations. In Step 2, a systematic review of the scientific literature is undertaken to identify prior studies that have tested the efficacy of vaccination messages on key targeted outcomes such as vaccine intentions, hesitancy, and uptake. Step 3 entails evaluating the cultural appropriateness and sensitivity of the messages identified in Steps 1 and 2 through participatory research methods—such as focus groups, interviews, or community advisory boards—with members of the target population. This participatory evaluation aims to provide critical insights into the degree to which existing messages resonate with the lived experiences, values, and communication norms of underserved communities, thereby informing culturally attuned message development in subsequent phases.

#### Phase 2. elicitation research

The objective of Phase 2 is to identify the core beliefs, perceptions, and motivational determinants of vaccination decision-making within the target population, grounded in established behavioural theory. This phase consists of Step 4, which involves the use of qualitative elicitation methods—such as in-depth interviews, focus groups, or open-ended survey items—to identify the salient beliefs that influence vaccine-related attitudes, intentions, and behaviours. Drawing on established frameworks that have been applied to identify health behaviour determinants and the mechanisms involved, and to inform interventions particularly those using messages and persuasive communication, such as the theory of planned behaviour (Ajzen, 1991), the health action process approach (Schwarzer & Hamilton, 2020; Schwarzer, 2008) or integrated models of behaviour (Hagger & Hamilton, 2020), this step is designed to source the culturally-specific beliefs that can be leveraged in message design. The findings inform the development of theoretically guided, culturally resonant message content in later phases.

#### Phase 3. Theoretical research

The objective of Phase 3 is to empirically test the extent to which determinants generated during the formative (Phase 1) and elicitation (Phase 2) phases relate to key outcomes salient to vaccination messaging, such as vaccination intentions and hesitancy, based on an integrated model of vaccination behaviour that synthesises insights from behavioural theory. This phase involves Step 5, which entails the development and administration of psychometric measures of constructs derived from the prior phases and quantifying their association with these outcomes in a sample of participants from the target population. Guided by integrated theoretical frameworks (Hagger & Hamilton, 2020), the model is expected to include constructs identified as salient in the earlier phases, such as perceived risk, social norms, self-efficacy, and trust in health authorities. Multivariate statistical analyses (e.g. regression, structural equation modelling) are used to determine the relative strength of these predictors. The goal is to isolate key belief constructs that can be strategically targeted in the design of culturally tailored vaccine messages in the subsequent phase.

#### Phase 4. Applied research

The objective of Phase 4 is to utilise the insights from the steps of the preceding phases to develop and refine prototype vaccination messages that are culturally appropriate and tailored to the specific needs of the target population. This phase includes Step 6, which focuses on the creation of draft messages informed by the key belief-based constructs identified in previous phases, followed by participatory validation to confirm their cultural relevance and sensitivity. Subsequently, Step 7 involves rigorous testing of the prototype messages to assess their acceptability, feasibility, appropriateness, and preliminary efficacy in promoting vaccination intentions and allying hesitancy. This evaluation examines the messages’ potential to affect change in these critical outcomes in groups representative of the targeted community. Iterative feedback and empirical data from these feasibility and pilot studies guide further refinement, ensuring that the final messages are contextually resonant and their potential efficacy in changing salient vaccination outcomes.

## Materials and methods

**Figure 1.**
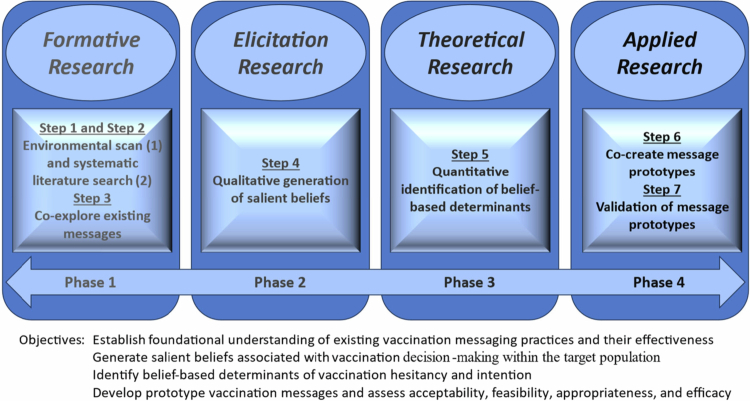
A Four-Phase Framework for Culturally Responsive Vaccine Messaging.

The proposed framework employs a multidisciplinary, mixed-methods ‘design science’ approach (Peffers et al., 2007) and comprises seven sequential steps across four integrated phases (see Figure 1). The evidence generated through this process guides the development and pilot testing of the primary deliverable: a set of prototype vaccination messages that are culturally responsive and contextually tailored. These prototype messages are then primed for subsequent efficacy evaluation via large-scale randomised controlled trials within the target population. The following section provides an overview of each phase and its associated steps, with illustrative examples drawn from efforts to develop culturally relevant COVID-19 vaccination for Latinos living in the California Central Valley, a key underserved community group known to have high rates of vaccine hesitancy and low uptake rates relative to the California and broader national population (Centres for Disease Control & Prevention, 2024; Moon et al., 2023; Riley et al., 2021; Scott et al., 2022). The proposed framework and protocol for the subsequent research studies was reviewed and approved by the Institutional Review Board (IRB) of the University of California, Merced.

### Phase 1 and Phase 2

Phase 1 utilises existing message analysis, literature reviews, and co-design methods to generate formative evidence to inform the development of first-draft prototypes of the culturally appropriate messages in subsequent phases. The evidence base is built using data from an environmental scan of governmental sources (Step 1) and a scoping or systematic review of the scientific literature on existing vaccine promotion messaging interventions (Step 2). This is followed by participatory research involving focus group discussions with members of the target population to evaluate and contextualise the messages identified in Steps 1 and 2 (Step 3). Phase 2 is then implemented using belief elicitation techniques to identify and map the set of beliefs that the target population deems most salient in their decision-making process regarding vaccination uptake (Step 4).

## Step 1 (Phase 1): environmental scan

Step 1 adopts an *environmental scan* methodology (Charlton et al., 2019)^1^, involving a systematic search of digital and online media, as well as national or sub-national government and broader health authority websites, to identify officially sanctioned vaccination messages. These messages are expected to originate from public health campaigns disseminated through a range of channels, including print media (e.g. newspapers, magazines, posters, and billboards), broadcast media (e.g. television and radio announcements), and digital platforms such as social media.

**Sampling method.** The systematic search targets digital and online media hosted on national or sub-national government public health websites (e.g. California state government), prominent national or regional health authority websites (e.g. the Centres for Disease Control and Prevention), community health organisation or non-profit websites specific to the nation or sub-national region (e.g. those serving California’s Central Valley), as well as websites for hospitals or clinics operating within the specified geographic area. These source websites are identified through structured queries using a web search engine (e.g. Google).

To be eligible for inclusion, vaccination messages must meet the following criteria:

1. Be published in a language commonly used by the target population (e.g. English and Spanish if targeting Latinos);
2. Originate from a credible national or sub-national health authority (e.g. government agency, non-profit organisation, or healthcare institution);
3. Specifically address the vaccine of interest (e.g. COVID-19 vaccine); and
4. Be released during a period of relevance (e.g. for the COVID-19 vaccine, this would be defined as on or after December 22, 2020—the date when the Pfizer-BioNTech COVID-19 vaccine became available to individuals aged 16 years and older).

For example, using this criteria, on identified websites as described above, the search terms ‘vaccination’/‘vacunación’, ‘vaccine’/‘vacuna’, ‘COVID’, and ‘COVID-19’ are entered in both English and Spanish to locate relevant messages. If a website does not contain a search feature, each page of the website is visited to identify messages related to COVID-19 vaccination. If a website provides a link to an organisation’s social media page, its social media feed is checked for messages that meet eligibility criteria.

**Data analysis.** The data are analysed using content analysis (Bengtsson, 2016) to extract, code, and collate discrete messages, which are then categorised according to the belief-based psychological constructs they target derived from typical social cognition theories applied in health behaviour contexts (Abraham et al., 2013; McMillan & Conner, 2007) and the behaviour change techniques (BCTs) they employ (Michie et al., 2013). This process facilitates the identification of messages that are representative of existing public health interventions and campaigns. Extracted data on the psychological constructs and BCTs associated with each message are systematically recorded in a spreadsheet. Descriptive statistics, including frequency counts, are computed to quantify the prevalence of specific constructs and techniques across the dataset.

### Step 2 (Phase 1): systematic literature search

Parallel to Step 1, Step 2 involves a systematic search of the scientific literature to identify studies that have empirically tested messages related to the target vaccine (e.g. such as those related to COVID-19) on outcomes including vaccine intentions, vaccination behaviour, and vaccine hesitancy.

**Sampling method.** Searches of digital databases are conducted by the Preferred Reporting Items for Systematic Reviews and Meta-Analyses (PRISMA) guidelines (Page et al., 2021). Studies are eligible for inclusion if they meet the same criteria outlined in Step 1 and explicitly report at least one vaccination message (e.g. COVID-19 vaccine message), either within the main research article or in supplementary materials. If possible, the search strategy is developed in collaboration with a university health sciences librarian to ensure comprehensiveness and methodological rigour. For example, a search for COVID-19 vaccination could be conducted using the scientific databases of MEDLINE (Ovid), Web of Science, PubMed, and PsycInfo and using the following search strings: (‘COVID-19’ OR ‘covid-19’ OR ‘coronavirus’ OR ‘2019-ncov’ OR ‘sars-cov-2’ OR ‘cov-19’ OR ‘COVID’) AND (‘vaccin*’) AND (‘messag*’ OR ‘intervention*’ OR ‘campaign*’). Search could be restricted by language but limited to those published on or after December 22, 2020.

**Data analysis.** Data from vaccination messages extracted from the studies identified in the systematic searches are analysed using narrative synthesis methods (Popay et al., 2006). In addition to coding each message for the belief-based psychological constructs targeted and the BCTs employed, additional information is recorded, including the type of study design (e.g. intervention study, randomised controlled trial, study protocol), whether the study assessed the efficacy of the messaging intervention in promoting vaccination, and—where available—effect size data related to intervention outcomes. All extracted data are organised into a structured spreadsheet, and descriptive statistics outlining prevalence and usage are generated.

### Step 3 (Phase 1): co-explore existing messages

Step 3 adopts focus group methods with participants from the target population to gain insight into their comprehension and perceptions of cultural responsiveness toward the existing messages for the target vaccine (e.g. COVID-19 vaccine) identified in Step 1.

**Sampling method.** The target population is recruited to participate (e.g. California Central Valley residents who self-identify as Latino, recruited from the local population in Madera County, California). Community networks (e.g. community health workers from the Camarena Health Promotores de Salud programme), data recruitment agencies, or convenience sampling methods are used to recruit participants. Participants are expected to have varying experience with the target vaccination and varying levels of vaccine hesitancy. Eligibility criteria are specified (e.g. adult residents of the California Central Valley who self-identify as Latino). Focus groups (*N* = 3 to 4) comprising six to ten participants and can be mixed or single sex, or a combination of the two, consistent with the specific cultural sensitivities of the target population and also with due consideration paid to the need to canvass views from all members of the community. Focus groups should be conducted in the first- or normatively-spoken language of the target population, as determined by participant preference, unless there is good reason to conduct them in an alternative language, such as in communities where bilingualism is a norm. Each focus group discussion is moderated by a trained facilitator guided by a semi-structured interview protocol.

**Measures and procedure.** A semi-structured interview protocol is developed to guide the focus group and prompt participants’ views on the comprehension and cultural appropriateness of existing vaccination messages for the target vaccine. The semi-structured interview protocol (for example, see Appendix A, Supplemental Material) is developed by the project team in close consultation with an established advisory group comprised of members of the target population who offer feedback and suggestions in a 1-hour protocol development meeting and consistent with prior research (Umaña-Taylor & Bámaca, 2004). The representative messages (approx. *N* = 4 to 6) identified in the scan and review conducted in Steps 1 and 2, respectively, serve as target stimuli for focus group discussions. After being presented with a detailed overview of the study and its expectations, participants are asked to complete an informed consent form. Next, participants complete an initial ‘icebreaker’ activity and a vaccination understanding exercise, led by the facilitator. Thereafter, participants are presented with each representative vaccination message in sequence and, after each, asked a series of questions by the facilitator. For each message, participants are asked to discuss their initial thoughts and interpretation of the message, evaluate its perceived effectiveness of promoting vaccination for themselves and, importantly, their community, and provide suggestions concerning how it could be improved. Discussions are expected to last for about one hour and are audio recorded. A member of the study is present at each focus group to make notes on the details of the discussions and their first impressions of central themes expressed, which is used as an additional information source in conjunction with the data analysis, where applicable. Audio recordings are transcribed using appropriate software with a rigorous follow-up check for transcription errors by a project team member.

**Data analysis**. Qualitative thematic analysis (Braun & Clarke, 2006) is used to identify themes relating to message comprehension and cultural relevance from the focus group discussion transcripts. Data collation and theme organisation are collated and summarised on annotated spreadsheets.

### Step 4 (Phase 2): qualitative generation of salient beliefs

Step 4 adopts qualitative, open-ended survey methods to elicit the beliefs that participants from the target population consider most salient when deciding whether to receive the target vaccine. The protocol is adapted from standardised belief elicitation techniques that have been widely used in prior research (Ajzen, 2002; Hamilton & White, 2010).

**Sampling and procedure**. A qualitative open-ended survey is conducted in a sample of the target population (e.g. residents self-identifying as Latino living in the California Central Valley). Eligible criteria for inclusion are established (e.g. candidate participants are required to be adult residents of one of the following counties that make up the San Joaquin Valley, often referred to as the Central Valley of California: Butte, Colusa, Fresno, Glenn, Kern, Kings, Madera, Merced, Placer, Sacramento, San Joaquin, Shasta, Stanislaus, Sutter, Tehama, Tulare, Yolo, and Yuba). Participants are anticipated to exhibit a range of vaccination experiences and degrees of hesitancy and recruited via the methods established in Phase 1. A sample of participants from the targeted community (*N* ~ 50) is recruited, with quotas designed to align with the distribution of key population characteristics. The open-ended survey questions are administered either through an online platform, over the phone, or in face-to-face settings and are delivered in the preferred language of the target population. Participants are prompted to respond to questions exploring personal, normative, and control-related beliefs relevant to their decision to receive the target vaccine.

**Measures.** The open-ended survey is developed by the project team based on established belief elicitation procedures (Ajzen, 2002) and translated into the preferred language of the target population. The survey comprises a series of open-ended questions prompting participants to report their behavioural beliefs representing their perceived costs and benefits of getting the vaccine (e.g. ‘What do you see as the advantages of getting [insert target vaccine]?’), their normative beliefs reflecting their perceived expectations and support of important others for getting the vaccine (e.g. ‘Please list the people who are important to you who might encourage you to get [insert target vaccine]’), and their control beliefs relating to the barriers thought to interfere with getting vaccinated (e.g. ‘Please list any factors or circumstances that would make it difficult or prevent you or someone in your community from getting [insert target vaccine]’) or facilitate or motivate getting the vaccine (‘Please list any factors or circumstances that would make it easy or enable you or someone in your community to get [insert target vaccine]’). For a list of example survey questions, see Appendix B, Supplemental Materials.

**Data analysis**. Data is analysed using content analysis consistent with prior research (Hamilton & White, 2010). Participants' responses to the open-ended questions are collated, listed, and content analysed for the beliefs in each category—behavioural, normative, and control beliefs—and their frequency of occurrence computed. Coding of the responses into the belief groups is conducted by a trained researcher. Frequency data for each set of beliefs is entered into a spreadsheet, and descriptive statistics of sample demographics and sample-level frequencies of the extracted themes are calculated.

#### Phase 3

Phase 3 focuses on identifying the key belief-based psychological correlates of intentions and hesitancy toward the target vaccine within the target population (Step 5). The findings are expected to inform the development of culturally responsive messages aimed at promoting vaccination and reducing hesitancy.

### Step 5 (Phase 3): quantitative identification of belief-based determinants

Step 5 is a quantitative, cross-sectional observational survey study that examines the relationships between the belief-based psychological constructs identified in the belief-elicitation study (Step 4), relevant social psychological constructs identified in the empirical literature, and intentions and hesitancy toward the target vaccine within the target population.

**Sampling method and measures**. Study participants and eligibility criteria are identical to those in Step 4. The projected sample size is determined through an a priori statistical power analysis. For example, where there are similar or equivalent prior studies that have tested effects of conceptually similar psychological determinants of behavioural outcomes in the vaccination behaviour domain, a candidate analytic technique would be path analysis or structural equation modelling analysis, and projected sample size could be estimated using the model fit derived from the conceptually similar analysis (see MacCallum et al., 1996). Sampling quotas reflecting the distribution of characteristics in the target population are applied where feasible and relevant.

The online questionnaire begins with a landing page that provides study details and outlines participant expectations, followed by an informed consent section detailing participants’ rights with a button click affirmation or opt-out and exit. The survey first collects demographic information (e.g. age, sex, employment status, annual income, education level), followed by scaled items assessing salient theory-based constructs reflecting the behavioural, normative, and control beliefs expected to be related to intentions to receive the target vaccine as specified in the proposed integrated model on which the study is based and consistent with prior theories that have been applied to predict health behaviour (Hagger et al., 2020). These belief items are developed based on the content analysis from the belief elicitation study (Step 4). Participants then complete items measuring global, direct summary measures of expected belief-based constructs derived from the typical theories, such as attitudes, subjective norms, risk perceptions, and perceived behavioural control, as an accompaniment to the specific belief-based measures with respect to the prospect of getting vaccinated. In addition, participants complete measures of past vaccination behaviours, including whether receipt of the target vaccine, if applicable, and intentions to receive the vaccine, following published guidelines (Ajzen, 2002). Finally, participants report general vaccination concerns and hesitancy, including fears related to the target vaccine, using an adapted version of the Vaccine Concerns in COVID-19 Scale (VACCS) (Hamilton & Hagger, 2022). Example items for the updated COVID-19 vaccine, adapted from prior research and guidelines, are provided in Appendix C of the Supplemental Materials to illustrate the expected structure and content of the survey.

**Data analysis**. Composite scores and prior analytic statistics, such as internal consistency estimates, are computed for each belief-based psychological construct in the first instance to describe the sample of the whole and provide some support for the psychometric integrity of measures used. Fit of a model specifying theory-implied relationships between belief-based constructs and the key outcomes of interest, vaccination intentions and hesitancy, is estimated using regression-based analytic techniques, permitting simultaneous analysis of theory-based nomological networks of associations such as path analysis or structural equation modelling. The model would be expected to include simultaneous effects of behavioural, normative, and control belief measures, direct measures of attitudes, subjective norms, and perceived behavioural control, vaccination concern measures, and key sample-based demographic variables. The effects of belief-based constructs and demographic factors on vaccine intentions and hesitancy are hypothesised to be indirect, mediated by attitudes, subjective norms, and perceived behavioural control.

#### Phase 4

Phase 4 involves the co-creation of prototype vaccine messages for the target vaccine, informed by the evidence generated in Phases 1 through 3. This phase includes conducting focus groups (Step 6) and a pilot feasibility study (Step 7) to gather feedback from the target population and identify insights that inform the further refinement of the final project deliverable—a culturally informed set of messages designed to promote uptake of the target vaccine.

### Step 6 (Phase 4): co-create message prototypes

Step 6 adopts intervention mapping techniques and focus group interviews to develop and obtain feedback on prototype messages aimed at increasing intention to receive the target vaccine and reducing vaccine hesitancy within the target population. Step 6 consists of three sequential stages.

**Sampling method, measures, and data analysis**. In the first stage, draft prototype messages are developed using an intervention mapping approach (Bartholomew et al., 1998), drawing on data and findings from the prior three Phases. This approach translates the key belief-based correlates of intentions to receive the target vaccine and vaccine hesitancy within the target population into message content designed to modify those beliefs. It is based on the assumption that belief-based constructs reliably associated with key outcomes—namely, vaccination intentions and hesitancy—reflect the cognitive and motivational processes underlying decision-making and behavioural tendencies related to vaccination (Sheeran et al., 2017). The approach further assumes that these beliefs are modifiable through persuasive communication specifically tailored to address them (Hamilton & Johnson, 2020). For example, if individuals express anxiety or concern regarding vaccine safety, messages emphasising the rigorous safety protocols and reliability of vaccine development processes may help to alleviate these concerns, thereby enhancing vaccination intentions and reducing hesitancy. A comprehensive discussion of the theoretical assumptions underpinning this approach is described elsewhere (Hagger & Hamilton, 2022b).

In addition, when developing message prototypes, the project team must ensure that the content aligns with the cultural norms and preferences of the target population. A key source of information for this process is the testimonies of participants from the Phase 1 (Step 3) focus groups—particularly their expressed preferences and aversions regarding existing messages—which are systematically mapped onto the language and content of the draft prototype messages. Furthermore, message tailoring is inherent in the methods used to identify the belief-based constructs targeted by the messages. The belief elicitation (Step 4) and subsequent confirmatory analysis (Step 5) draw on multiple samples from the target population, enabling the project team to identify beliefs perceived as most salient, while implicitly excluding beliefs deemed non-salient. Collectively, these methods ensure that the messages developed are meaningfully tailored to the specific population group.

In the second stage, an additional set of focus groups is conducted in new samples of individuals from the target population. Participant recruitment methods and eligibility criteria are identical to those used in Phase 2 (Step 4). The focus groups are guided by a semi-structured interview protocol similar to that employed in Step 4 and are moderated by a trained facilitator. Unlike Step 4, however, the purpose of these interviews is to gather participant feedback on the comprehension, clarity, acceptability, and feasibility of the draft prototype messages developed in Stage 1. As in Step 4, the session begins with the facilitator providing study information and obtaining informed consent. Participants then complete an introductory icebreaker activity and a vaccination understanding exercise. Each prototype message is presented in turn, and participants are invited to share their initial impressions, perceptions of the message’s effectiveness in promoting vaccination for themselves and their communities, and suggestions for improving the message’s impact. Discussions are expected to last approximately one hour and are audio recorded. A project team member attends each focus group to take notes, which support subsequent data analysis. Audio recordings are transcribed using appropriate software, followed by manual cheques for transcription accuracy by a project team member. An outline of the focus group protocol is provided in Appendix D, Supplemental Materials. Transcripts are analysed using qualitative thematic analysis (Braun & Clarke, 2006).

In the third and final stage, the project team revise the draft prototype messages based on findings from the focus group analysis, with particular attention to tailoring the language to maximise comprehension, acceptability, appropriateness, and clarity within the target population. The aim is to produce a set of prototype messages, each consisting of contextualising passages that introduce the targeted behaviour, followed by key persuasive content designed to promote intentions to receive the target vaccine and reduce vaccine hesitancy. The messages are text-based and will not include any visual or infographic elements.

### Step 7 (Phase 4): validation of message prototypes

The final step, Step 7, aims to conduct an initial evaluation of the feasibility of the draft prototype messages developed in Step 6 within the target population. The primary focus of this study is to assess the feasibility, acceptability, and appropriateness of the messages, rather than their efficacy. However, parallel measurement of key targeted outcomes—specifically, intentions to receive the target vaccine and levels of vaccine hesitancy—may also be included to provide preliminary insights into the messages’ potential impact.

**Sampling method, measures, and data analysis**. Step 7 adopts a repeated-measures (pre–post) intervention design to conduct an initial evaluation of the feasibility, acceptability, and appropriateness (Weiner et al., 2017) of the prototype messages developed in Step 6 within the target population. While the primary focus is on assessing implementation outcomes, parallel measurement of key behavioural outcomes—namely, intentions to receive the target vaccine and vaccine hesitancy—is also undertaken to explore potential preliminary effects of message exposure. This design aligns with established methodological frameworks for pilot and feasibility research in health communication interventions (Andrade et al., 2023; Ball et al., 2023).

Participants are recruited using the same procedures and eligibility criteria as those applied in Step 5. The study is conducted online using a secure survey platform. Participants are initially directed to a landing page containing study information, followed by an electronic informed consent form with assent provided via a button press. Participants subsequently complete baseline assessments, including measures of vaccine intentions and hesitancy, as well as ratings of message feasibility, acceptability, and appropriateness. These implementation constructs are operationalized through items assessing beliefs about the content, format, appeal, and relevance of the prototype messages. Participants are then exposed to the prototype messages in a randomised order. Following each message, participants are prompted to provide a summary in an open-text field to confirm message engagement. To reduce cognitive carryover effects, a brief, non-demanding filler task (e.g. a simple puzzle) is administered. Thereafter, participants complete post-intervention assessments of vaccine intentions and hesitancy. Data is analysed using descriptive statistics and appropriate inferential difference tests to evaluate changes in key variables across time points (e.g. paired-samples *t*-tests with Bonferroni correction applied to control for Type I error). The primary outcomes of interest are feasibility, acceptability, and appropriateness. An a priori power analysis is conducted to estimate the required sample size, based on a repeated-measures design with two time points. This preliminary study is expected to generate foundational evidence regarding the implementation viability of the prototype messages and offer early insights into their potential to influence vaccination intentions and hesitancy. Findings will inform the refinement of message content and delivery and serve as a basis for future full-scale efficacy testing in randomised controlled trials.

## Discussion

In this article, we present a novel, theory-informed, and culturally responsive framework for developing vaccine communication messages tailored to underserved minoritized populations. By integrating cultural responsiveness and community engagement practices with established behavioural theory, the proposed four-phase, seven-step framework addresses a notable gap in public health communication: the lack of systematic, theory-driven methods for designing and evaluating behaviour change messages that are responsive to, and tailored toward, the needs of individuals from historically underserved communities.

The significance of this framework lies in its holistic and participatory design, which encompasses a comprehensive process from initial formative research phases to pilot and feasibility testing. In Phase 1 of the framework, the triangulation of environmental scans, literature synthesis, and qualitative enquiry provides a comprehensive, detailed, and rich understanding of existing message landscapes and the extent to which they are culturally misaligned and not directly tailored to community needs. These insights establish foundational knowledge necessary to inform the belief elicitation process in Phase 2, which is purposed to uncover salient beliefs as well as attitudinal, normative, and control factors influencing vaccine decision-making sourced directly from individuals from the targeted underserved community of interest. The transition into quantitative assessment in Phase 3 is focused on providing broader converging evidence of the specific key belief-based determinants of vaccine hesitancy and intention from the targeted community, highlighting the needs to be considered when tailoring messages to potentially modifiable psychological determinants specifically for individuals from the community of interest. Finally, Phase 4 completes to process through translational procedures in which findings from the previous phases are developed into tangible message prototypes followed up with testing their real-world applicability and cultural appropriateness among members of the target community, ensuring both theoretical fidelity and community relevance and responsiveness.

This framework differentiates itself from previous procedural frameworks by explicitly embedding cultural values and community needs in the message development process, moving away from a sole focus of tailoring toward the demographic characteristic of the target community and toward a cultural and community need basis endemic in each phase of the design process. In doing so, it aligns with calls from scholars and public health practitioners for culturally situated interventions that move beyond superficial or tokenistic consideration of these factors toward a foundational, structural responsiveness that comprehensively captures the needs and perspectives of the targeted population. Moreover, the use of a progressive mixed method approach across the framework that integrates iterative focus group discussions with members of the target population with progressive belief elicitation, review, survey, and feasibility methods ensures face and construct validity of the developed messages and that the messages are appropriately responsive and tailored toward the target community.

Nevertheless, the framework is not without limitations. As a design science approach, its implementation may be resource-intensive, requiring multidisciplinary collaboration and sustained community engagement. The generalisability of findings from focus groups and feasibility trials may also be constrained by local cultural norms and needs, necessitating adaptation across contexts. Further, specialised training and skill development may be required for the project team to conduct the multiple methods adopted in each phase, including, but not limited to: training on focus group delivery in the first-spoken language of the target population; translation of materials, scripts, survey items, and responses; expertise in qualitative and quantitative data collation and analytic procedures; competences in intervention mapping and message tailoring; and feasibility trial design, implementation, analysis, and interpretation.

We also see this current framework as paving the way for future research in which the framework is applied across a broader range of health behaviour contexts and minoritized populations to provide broad comprehensive data on its value, adaptability, and scalability. However, we emphasise that although this framework is adaptable in principle, it is currently tailored to the distinctive sociocultural and psychological dynamics of vaccination communication that differentiate vaccination promotion from other health behaviours, including challenges such as politicisation, collective-risk dynamics, historical mistrust, and exposure to widespread misinformation, which should be considered when applying it to alternative contexts. Moreover, although this proposed framework is purposed to provide a theory-based and culturally responsive roadmap for the development of vaccine messaging, subsequent randomised controlled trials and field-based behavioural evaluations are necessary to evaluate the efficacy and applicability of messages developed using these guidelines; however, the conceptual and empirical foundations underpinning this framework provide strong justification for expecting improved downstream efficacy. Finally, future empirical work should directly compare messages developed using this framework to those developed using less structured approaches to evaluate their effectiveness and feasibility compared to more conventional messages.

In conclusion, the current framework contributes a robust and culturally responsive, community engaged approach to the development of vaccine messages targeted to the values and needs of typically underserved communities. The framework holds promise for enhancing the efficacy of public health campaigns aimed at reducing disparities in vaccine uptake and may serve as a transferable model for other health communication efforts in diverse and underserved communities.

## Supplementary Material

Supplementary Material A_Four_Phase_Framework_Supplemental_Materials_SubmittedCleanVersion.docx

## Data Availability

Not applicable for this paper. It is recommended that all data and materials be stored on an open-access platform to promote transparency and reproducibility.

## Endnotes

^1^
Environmental scans are a useful research method used by researchers and organisations to assist with decision making (Charlton et al., 2019). The process involves searching, locating, collating, interpreting, and utilising information from multiple sources to inform future decisions. Multiple data gathering strategies are used in this process including but not limited to, focus groups and interviews, surveys, literature reviews, and reviews of communications and policy documents. Unlike traditional literature review processes, the scan does not confine itself to formal research items, but encompasses multiple data sources.
