# Supplementary material for: A four-phase framework for culturally responsive vaccine messaging
Source: Health Psychol Behav Med. 2026 May 19;14(1):2666714. doi: 10.1080/21642850.2026.2666714 (PMC13217510; doi:10.1080/21642850.2026.2666714)
Supplement: Supplementary Material — A_Four_Phase_Framework_Supplemental_Materials_SubmittedCleanVersion.docx [file RHPB_A_2666714_SM3610.docx]

Appendix A

*Example Semi-Structured Interview Protocols for Step 3 (Phase 1)*

**English**

# **Focus Group 1a Protocol**

**Pre-Interview**

- "Welcome, thanks for coming in. I will explain the focus and purpose of today’s research study to the group soon. But before we can start, we have a few formalities. Could I ask you to read this form and brief survey, and, if you agree, please complete the consent form and return it to me. If you have any questions at all about this study please feel free to ask me now.”
- *Present participants with consent form and demographic survey*.
- *Participants provide written consent and completed demographic survey, collected by researcher/facilitator.*

# **Introduction**

- *Promotora introduces themself and their affiliation.*

“Welcome again, I would like to thank you for agreeing to participate in this focus group discussion for our research study. My name is [NAME] and my role is to facilitate this focus group discussion by asking questions and, if needed, asking for clarification or further information. My role is to also guide the conversation so we do not deviate from the topic of discussion.

- "Participating in this study today has two parts that will take about 1 hour and 30 minutes in total. The first part is a focus group discussion. When the focus group is done you will each take a survey privately.”
- “This is a ***focus group*** discussion, this means that I will ask the group questions, and anyone can volunteer to give an answer. It is meant to be a discussion, and all of you can contribute and give your responses. Also, not everyone has to agree with the views and opinions expressed, and there may be some differences in opinion, and this is completely natural, we encourage everyone to express their own views and perspectives on the ideas we discuss. There are no specific rules about giving your responses, you can just indicate that you are about to talk and go ahead and contribute. We just ask you all to please respect other people when they are talking and try not to interrupt. It is also important that if you disagree, please do so in a respectful, friendly way *<<smile>>*.”
- “In this focus group discussion, we are interested in exploring your views of the COVID-19 vaccines. So, I will ask the group a number of questions about some COVID-19 messages, and I expect those questions will be a starting point for the discussion. There are NO right or wrong answers so please respond as honestly as you can. We really value your opinion on this topic and so please feel free to share your opinions as openly as possible. I expect to spend about an hour discussing this issue, but there is no specific fixed time.”
- “Just a reminder, although you will have seen it on the form I just gave you, it is important to remember that we will not write down any information today that can identify you, and all the information and opinions we gather during this focus group will be recorded in such a way that no individual participant can be identified. So, please remember not to state your name or other people’s names during our discussion.”
- “The focus group session is being audio-recorded. Please try to speak as clearly as you can to help with the audio recording. This will allow me to focus on what you are saying and not rely on my memory. The audio tapes will be securely stored and only accessible to the researchers. When the study is completed, all audio recordings will be destroyed.”

# **Icebreaker activity**

- “To begin the discussion, I’m wondering if you would like to share a little about your hobbies or things you like to do?”

# **COVID Understanding Exercise**

- I’d also like to learn what you all think about COVID-19. What do you think it is?
- How do you think COVID-19 spreads from person to person?

# **Main Interview (Group Questions)**

- “The specific topic of today’s focus group will be a discussion about your views and opinions on a series of COVID-19 vaccine messages. We are particularly interested in how well you think these messages will be understood by people from this community in <<LOCATION>>, whether people and people like you will understand them, and whether they are clear and well-put or phrased, or whether could be improved. As a reminder, all information that you give in this focus group will be kept anonymous, so please do not state your name or anyone else’s name during this focus group.”
- “So I am now going read a series of passages to you which make up the COVID-19 vaccination messages I was talking about earlier. As you can see I have also projected the messages on the screen. Please listen to the messages, and read along if you like. All the while, please think about whether you think they would be effective in promoting you to get vaccinated, and whether they would be useful or effective for people in your community. Also, think about whether you think they could be improved, or whether there are other things that could be added or changed to improve understanding. Also, please also let me know if there is something you would like me to repeat”.

# **Message review: Appropriateness & Feasibility**


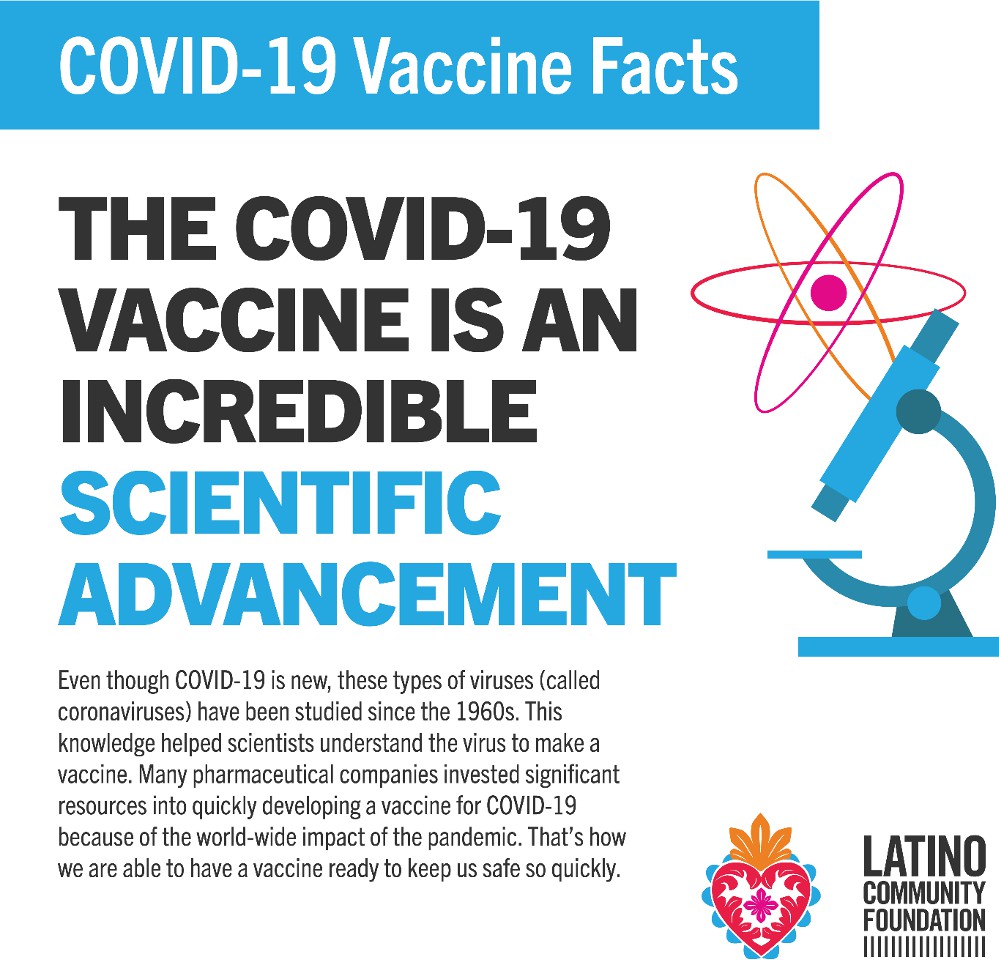


- What is your first reaction or initial thoughts when you see this message? [Do you think this message would work for you; for example, would you be persuaded to get vaccinated?] [Why or why not?]
- Do you think the message would be effective for the people in the community you interact and live with? [How so?] [How could it be improved?]


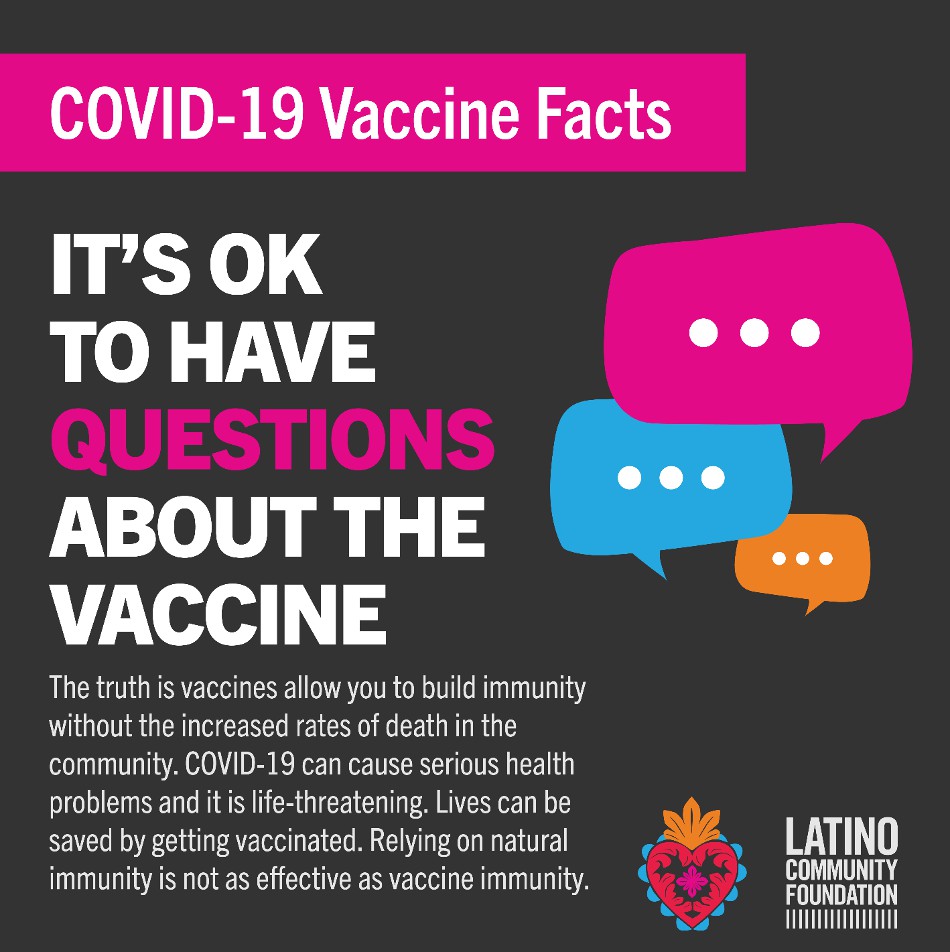


- What is your first reaction or initial thoughts when you see this message? [Do you think this message would work for you; for example, would you be persuaded to get vaccinated?] [Why or why not?]
- Do you think the message would be effective for the people in the community you interact and live with? [How so?] [How could it be improved?]


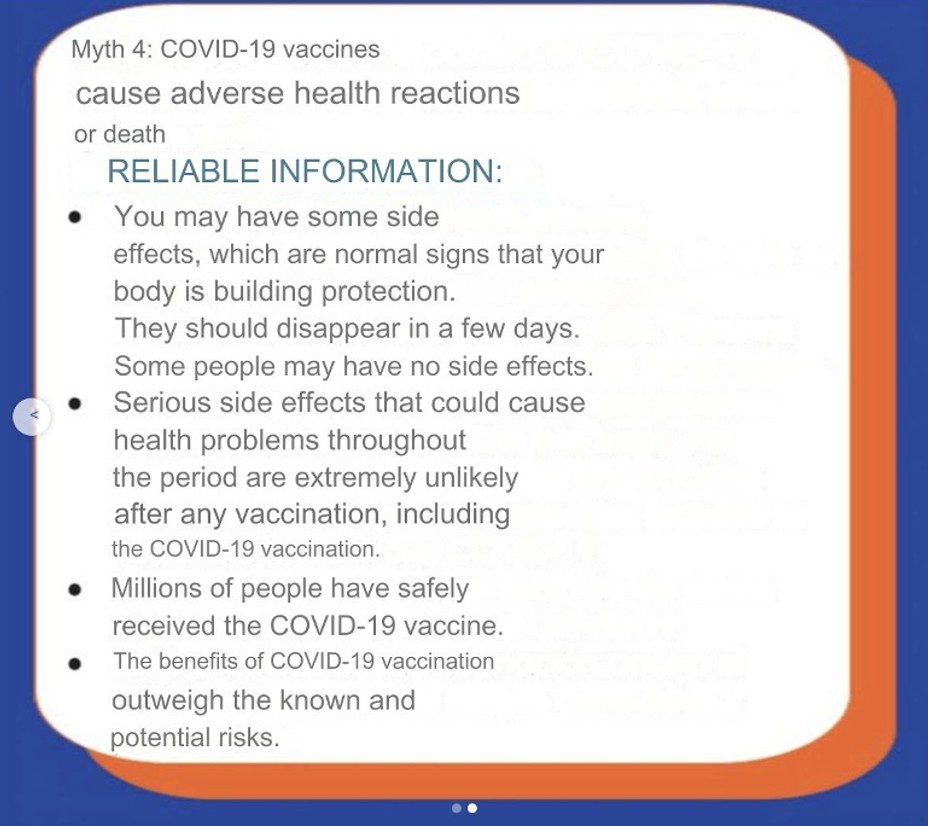


- What is your first reaction or initial thoughts when you see this message? [Do you think this message would work for you; for example, would you be persuaded to get vaccinated?] [Why or why not?]
- Do you think the message would be effective for the people in the community you interact and live with? [How so?] [How could it be improved?]


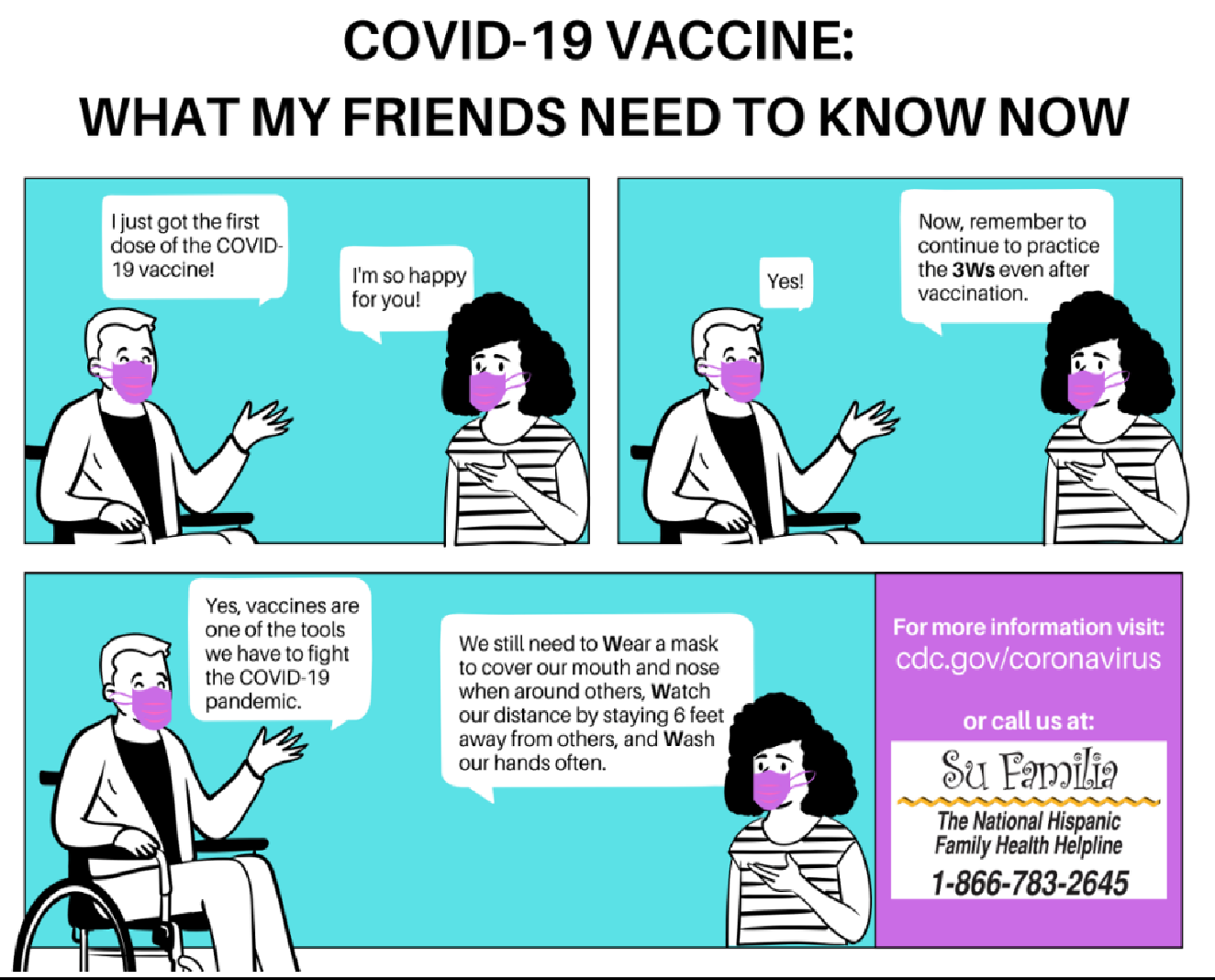


- What is your first reaction or initial thoughts when you see this message? [Do you think this message would work for you; for example, would you be persuaded to get vaccinated?] [Why or why not?]
- Do you think the message would be effective for the people in the community you interact and live with? [How so?] [How could it be improved?]


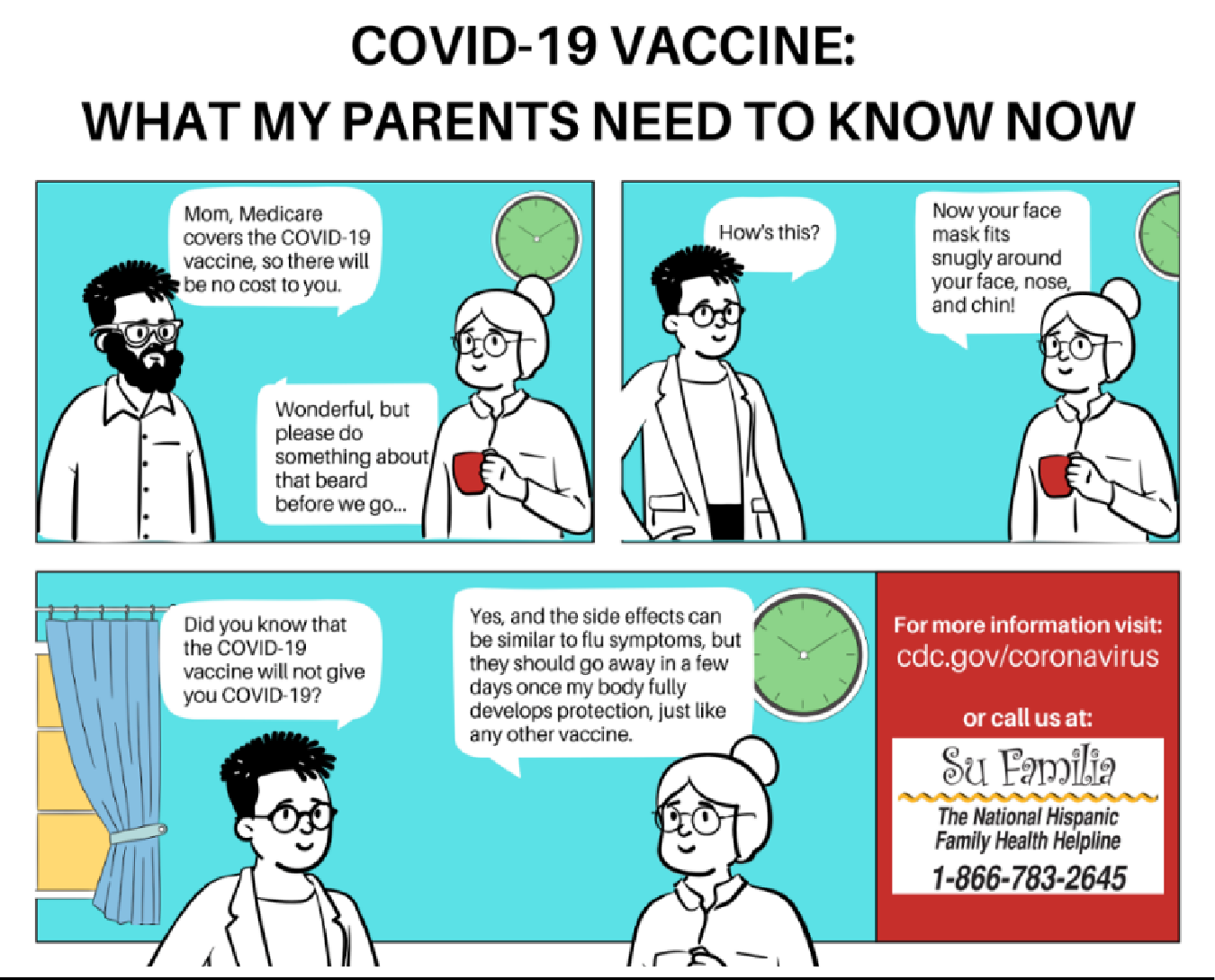


- What is your first reaction or initial thoughts when you see this message? [Do you think this message would work for you; for example, would you be persuaded to get vaccinated?] [Why or why not?]
- Do you think the message would be effective for the people in the community you interact and live with? [How so?] [How could it be improved?]


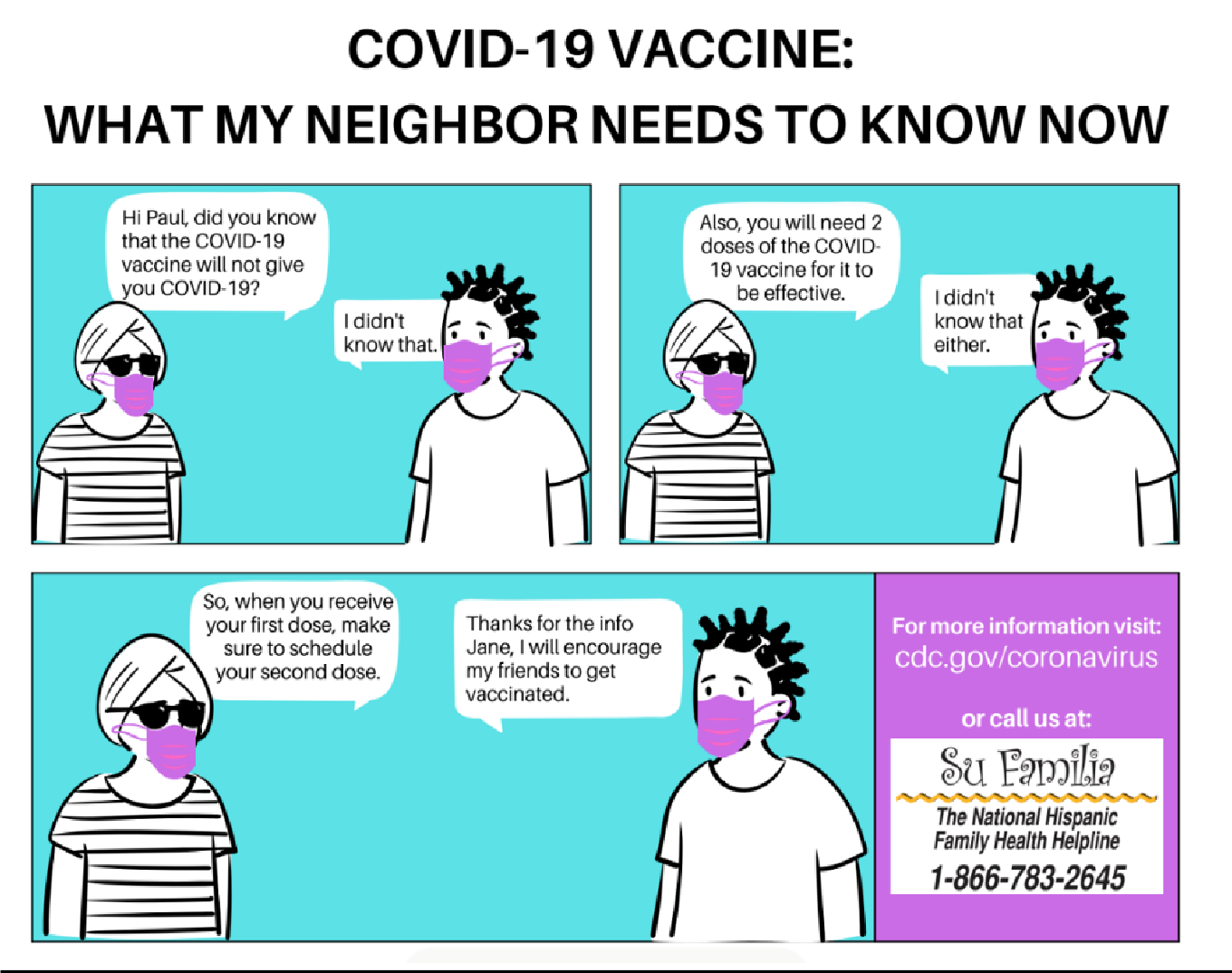


- What is your first reaction or initial thoughts when you see this message? [Do you think this message would work for you; for example, would you be persuaded to get vaccinated?] [Why or why not?]
- Do you think the message would be effective for the people in the community you interact and live with? [How so?] [How could it be improved?]

# **Post-Interview**

- - “Thank you very much for sharing your honest thoughts and opinions related to the COVID-19 vaccine. This concludes our focus group discussion.”
  - “Now, we are going to take a short 10-minute break and then I will ask each of you to complete a post-discussion survey. The topic is like our discussion today and is related to your opinions and beliefs about COVID-19”.
  - “The survey should take approximately 30 minutes, but some of you will need less or more time.”
  - “After you are done with the survey, please hand it in to me and I will give you your gift card for participating in our research study today. Thank you very much.”

**Spanish**

**Protocolo del grupo focal 1a**

**Antes del Grupo Focal**

- "Bienvenido, gracias por venir. Pronto les explicaré al grupo el enfoque y el propósito del estudio de investigación de hoy. Pero antes de que podamos comenzar, tenemos algunas formalidades. ¿Podría pedirles que lean esta forma y breve encuesta y, si están de acuerdo, por favor completen la forma de consentimiento y entréguenmelo. Si tienen alguna pregunta sobre este estudio, no duden en preguntarme ahora."
- *Entregue la forma de consentimiento y la encuesta de información demográfica a los participantes*
- *Los partcipantes entreguen la forma de consentimiento y la encuesta de información demográfica completa a los facilitadores*

**Introducción**

- *La facilitadora se presenta y tambien presenta a su afiliación*

“Bienvenido nuevamente, me gustaría agradecerle por aceptar participar en esta discusión de grupo focal para nuestro estudio de investigación. Mi nombre es [NOMBRE] y mi función es facilitar esta discusión de grupo focal haciendo preguntas y, si es necesario, solicitando aclaraciones o más información. Mi función también es guiar la conversación para que no nos desviemos del tema de discusión.”

- "Participar en este estudio hoy tiene dos partes que tomarán aproximadamente 1 hora y 30 minutos en total. La primera parte es una discusión de grupo focal. Cuando el grupo focal termine, cada uno de ustedes realizará una encuesta en privado".
- "Esto es un discusión ***de grupo de enfoque***, esto significa que haré preguntas al grupo y cualquiera puede ofrecerse como voluntario para dar una respuesta. Está destinado a ser una discusión y todos ustedes pueden contribuir y dar sus respuestas. Además, no todos tienen que estar de acuerdo con los puntos de vista y opiniones expresados, y puede haber algunas diferencias de opinión, y esto es completamente natural; alentamos a todos a expresar sus propios puntos de vista y perspectivas sobre las ideas que discutimos. No existen reglas específicas para dar tus respuestas, simplemente puedes indicar que estás a punto de hablar y seguir adelante y contribuir. Sólo les pedimos a todos que por favor respeten a las demás personas cuando hablan y traten de no interrumpir. También es importante que si no está de acuerdo, lo haga de manera respetuosa y amigable.*<<sonrisa>>*.”
- “En esta discusión de grupo focal, estamos interesados ​​en explorar sus experiencias con las vacunas COVID-19. Entonces, le haré al grupo una serie de preguntas sobre experiencias relacionadas con las vacunas COVID-19, y espero que esas preguntas sean un punto de partida para la discusión. NO hay respuestas correctas o incorrectas, así que responda lo más honestamenste posible. Realmente valoramos su opinión sobre este tema y, por lo tanto, siéntase libre de compartir sus experiencias de la manera más abierta posible. Espero dedicar aproximadamente una hora a discutir este tema, pero no hay un tiempo fijo específico”.
- “Solo un recordatorio, aunque habrá visto en el formulario que le acabo de dar, es importante recordar que hoy no escribiremos ninguna información que pueda identificarlo, ni toda la información y opiniones que recojamos durante este grupo focal. Se registrarán de tal manera que no se pueda identificar a ningún participante individual. Por lo tanto, recuerde no decir su nombre ni el de otras personas durante nuestra conversación.”
- “La sesión del grupo focal se está grabando en audio. Intente hablar lo más claramente posible para ayudar con la grabación de audio. Esto me permitirá concentrarme en lo que estás diciendo y no confiar en mi memoria. Las cintas de audio se almacenarán de forma segura y solo serán accesibles para los investigadores. Cuando se complete el estudio, todas las grabaciones de audio serán destruidas”.

**Rompehielo**

- “Para comenzar la discusión, pregunto si le gustaría compartir un poco sobre sus pasatiempos o las cosas que le gusta hacer.”

**Ejercicio de comprensión de COVID**

- También me gustaría saber qué piensan todos ustedes sobre el COVID-19. ¿Qué creen que es?
- ¿Cómo creen que se transmite el COVID-19 de persona a persona?

**Entrevista Principal (preguntas para el grupo)**

- “El tema específico del grupo focal de hoy será una discusión sobre sus puntos de vista y opiniones sobre una serie de mensajes sobre la vacuna COVID-19.. Estamos particularmente interesados ​​en saber qué tan bien cree que estos mensajes serán entendidos por las personas de esta comunidad en <<LOCATION>>, si personas como usted los entenderán, y si son claros y bien expresados ​​o redactados, o si podría ser mejorado. Como recordatorio, toda la información que proporcione en este grupo de enfoque se mantendrá anónima, así que no diga su nombre ni el de ninguna otra persona durante este grupo de enfoque”.
- “Así que ahora les leeré una serie de pasajes que componen los mensajes de vacunación COVID-19 de los que hablé antes. También he proyectado los mensajes en la pantalla. Escuche los mensajes y léalos si lo desea. Mientras, piense si cree que serían eficaces para promover que usted se vacune y si serían útiles o eficaces para las personas de su comunidad. Además, piense si cree que podrían mejorarse o si hay otras cosas que podrían agregarse o cambiarse para mejorar la comprensión. Además, por favor déjame saber si hay algo que te gustaría que repita”.

**Revisión de los mensajes: La Idoneidad y Viabilidad**


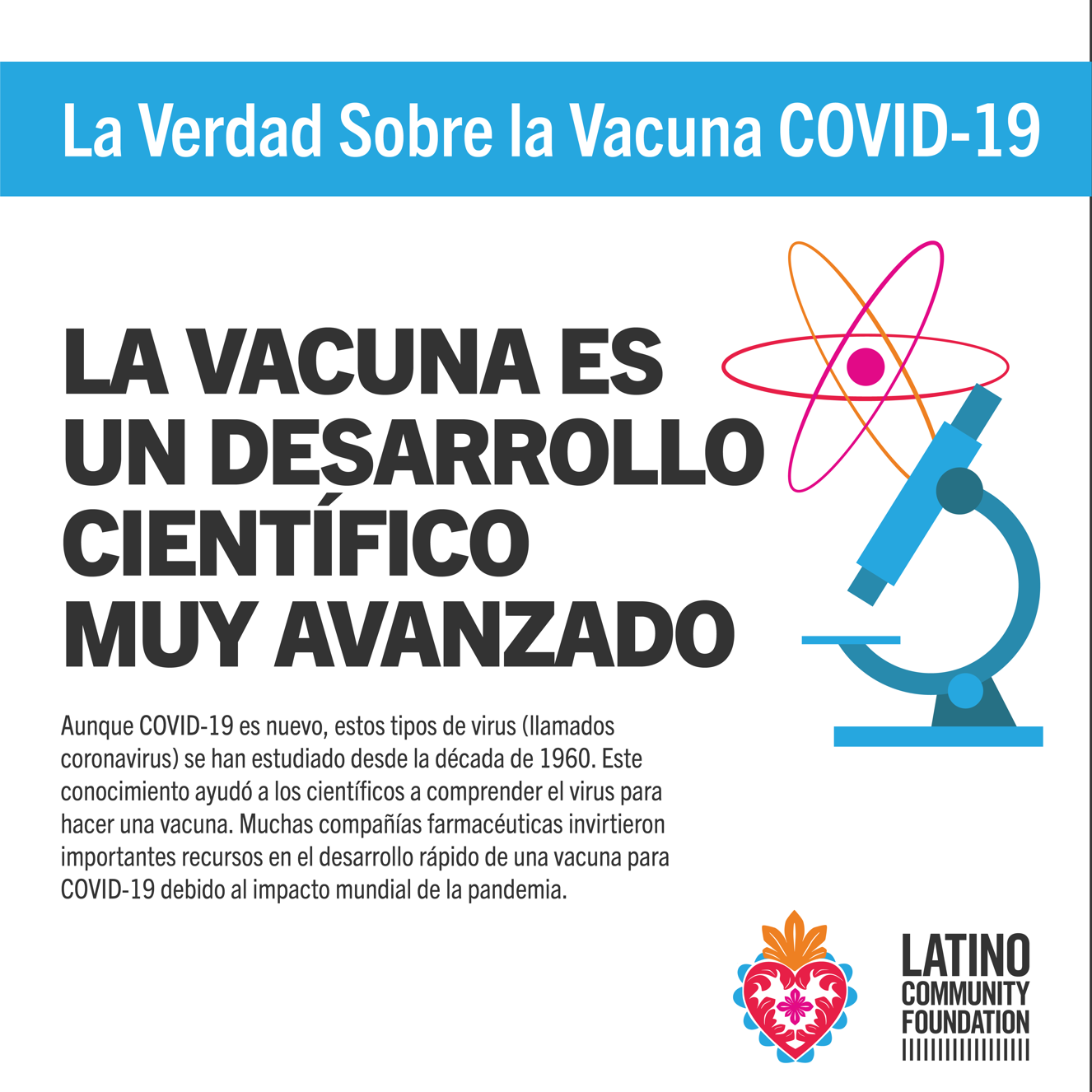


- ¿Qué es su primera reacción o qué son sus pensamientos iniciales cuando ves este mensaje? [**Indicaciones de seguimiento si se necesitan:** ¿Crees que este mensaje funcionaría para ti? Por ejemplo, ¿te convencería de vacunarte? ¿Por qué sí o por qué no?]
- ¿Cree que este mensaje sería efectivo para las personas de su comunidad? Piense en las person con que interactúas y con que vives. [**Indicaciones de seguimiento si se necesitan:** ¿Por qué o en que manera? ¿Cómo se podría mejorar?]


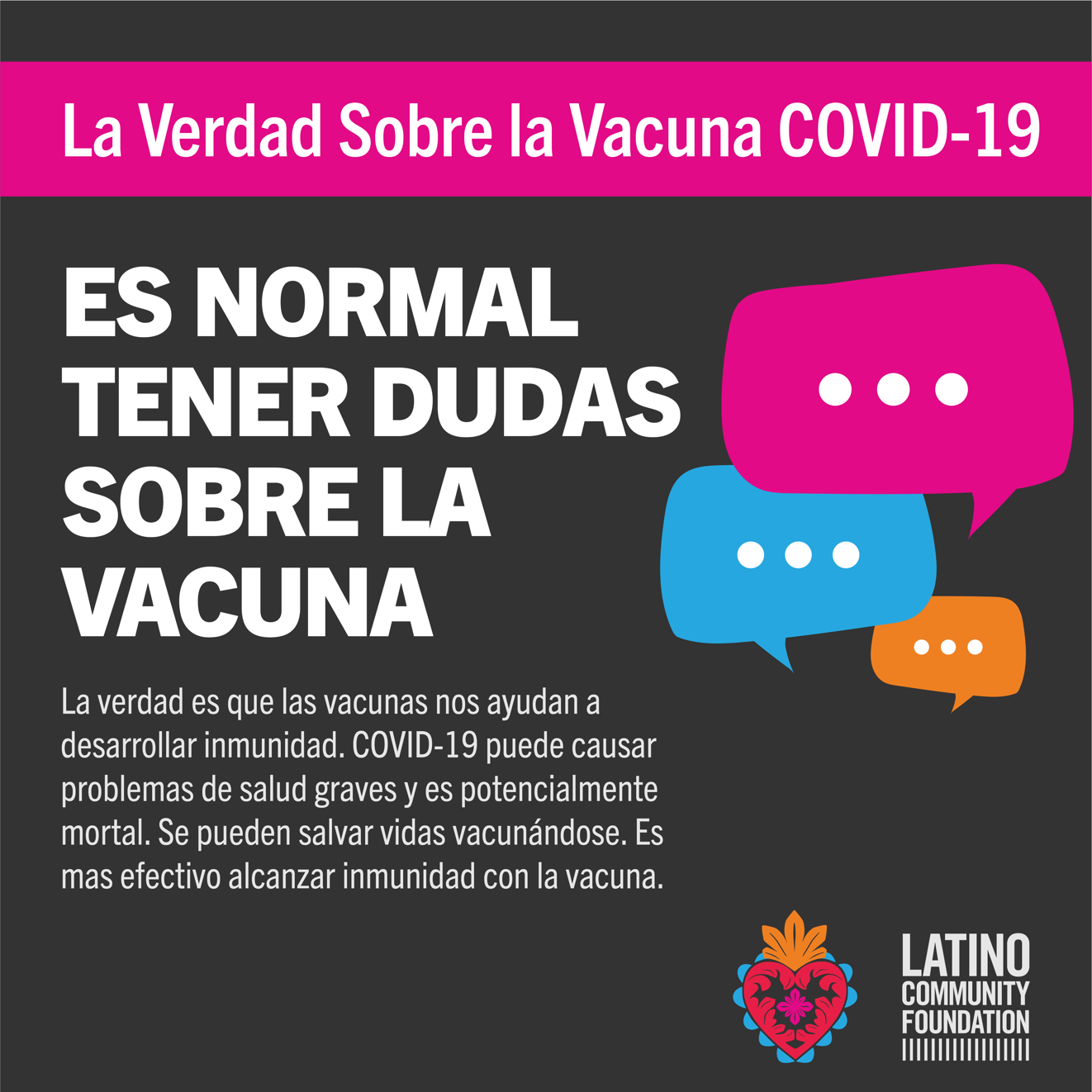


- ¿Qué es su primera reacción o qué son sus pensamientos iniciales cuando ves este mensaje? [**Indicaciones de seguimiento si se necesitan:** ¿Crees que este mensaje funcionaría para ti? Por ejemplo, ¿te convencería de vacunarte? ¿Por qué sí o por qué no?]
- ¿Cree que este mensaje sería efectivo para las personas de su comunidad? Piense en las person con que interactúas y con que vives. [**Indicaciones de seguimiento si se necesitan:** ¿Por qué o en que manera? ¿Cómo se podría mejorar?


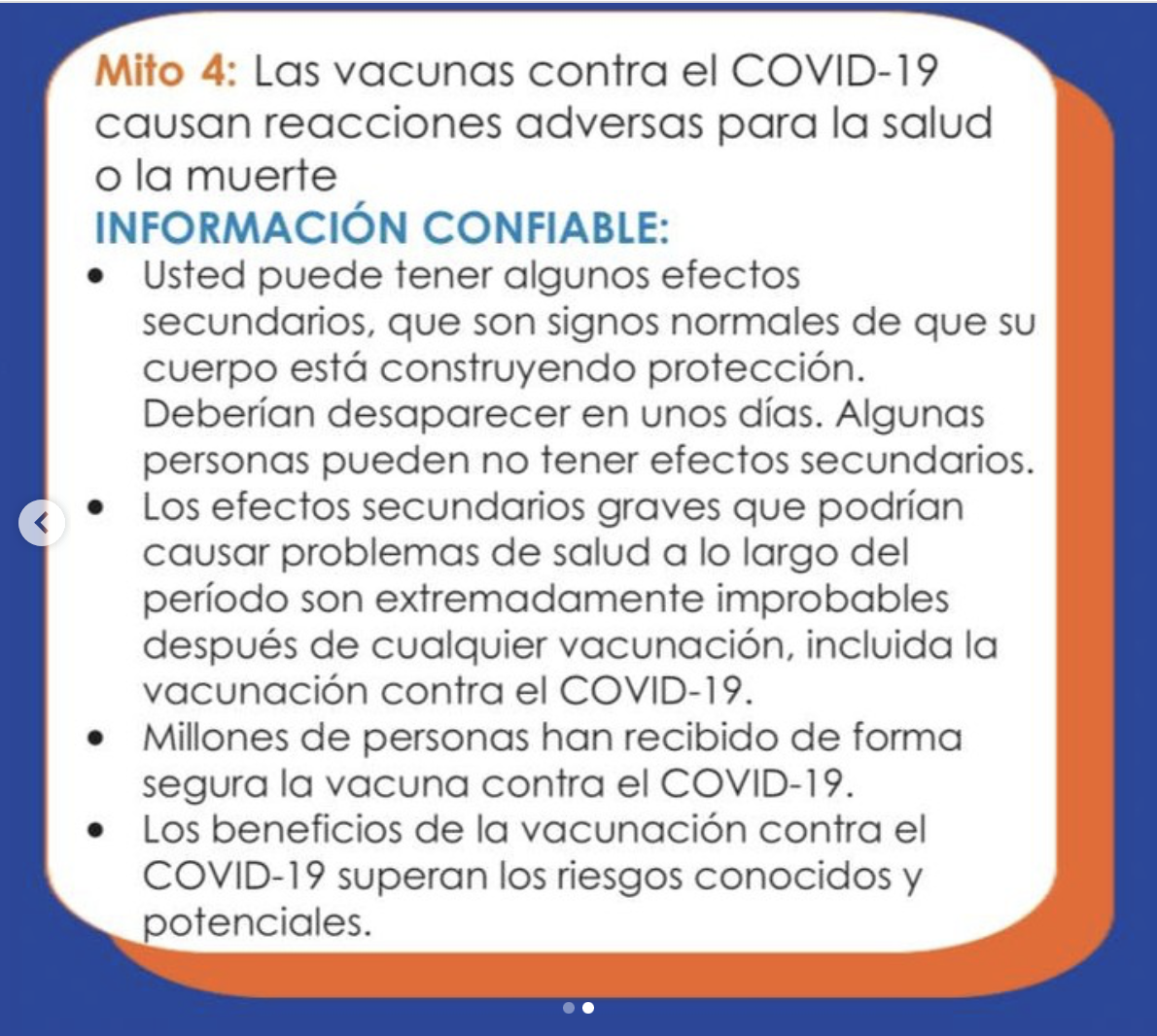


- ¿Qué es su primera reacción o qué son sus pensamientos iniciales cuando ves este mensaje? [**Indicaciones de seguimiento si se necesitan:** ¿Crees que este mensaje funcionaría para ti? Por ejemplo, ¿te convencería de vacunarte? ¿Por qué sí o por qué no?]
- ¿Cree que este mensaje sería efectivo para las personas de su comunidad? Piense en las person con que interactúas y con que vives. [**Indicaciones de seguimiento si se necesitan:** ¿Por qué o en que manera? ¿Cómo se podría mejorar?]

**
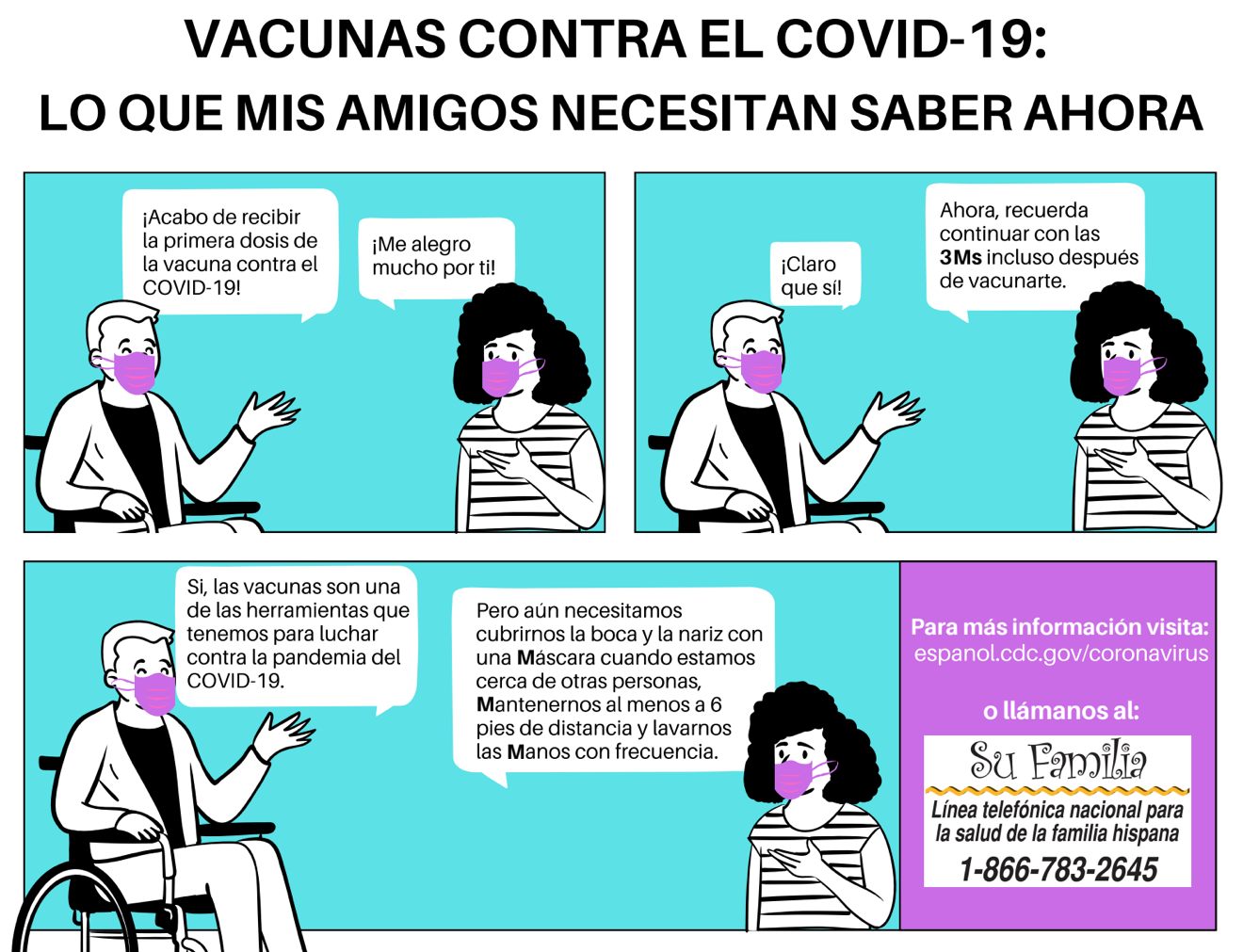
**

- ¿Qué es su primera reacción o qué son sus pensamientos iniciales cuando ves este mensaje? [**Indicaciones de seguimiento si se necesitan:** ¿Crees que este mensaje funcionaría para ti? Por ejemplo, ¿te convencería de vacunarte? ¿Por qué sí o por qué no?]
- ¿Cree que este mensaje sería efectivo para las personas de su comunidad? Piense en las person con que interactúas y con que vives. [**Indicaciones de seguimiento si se necesitan:** ¿Por qué o en que manera? ¿Cómo se podría mejorar?]

**
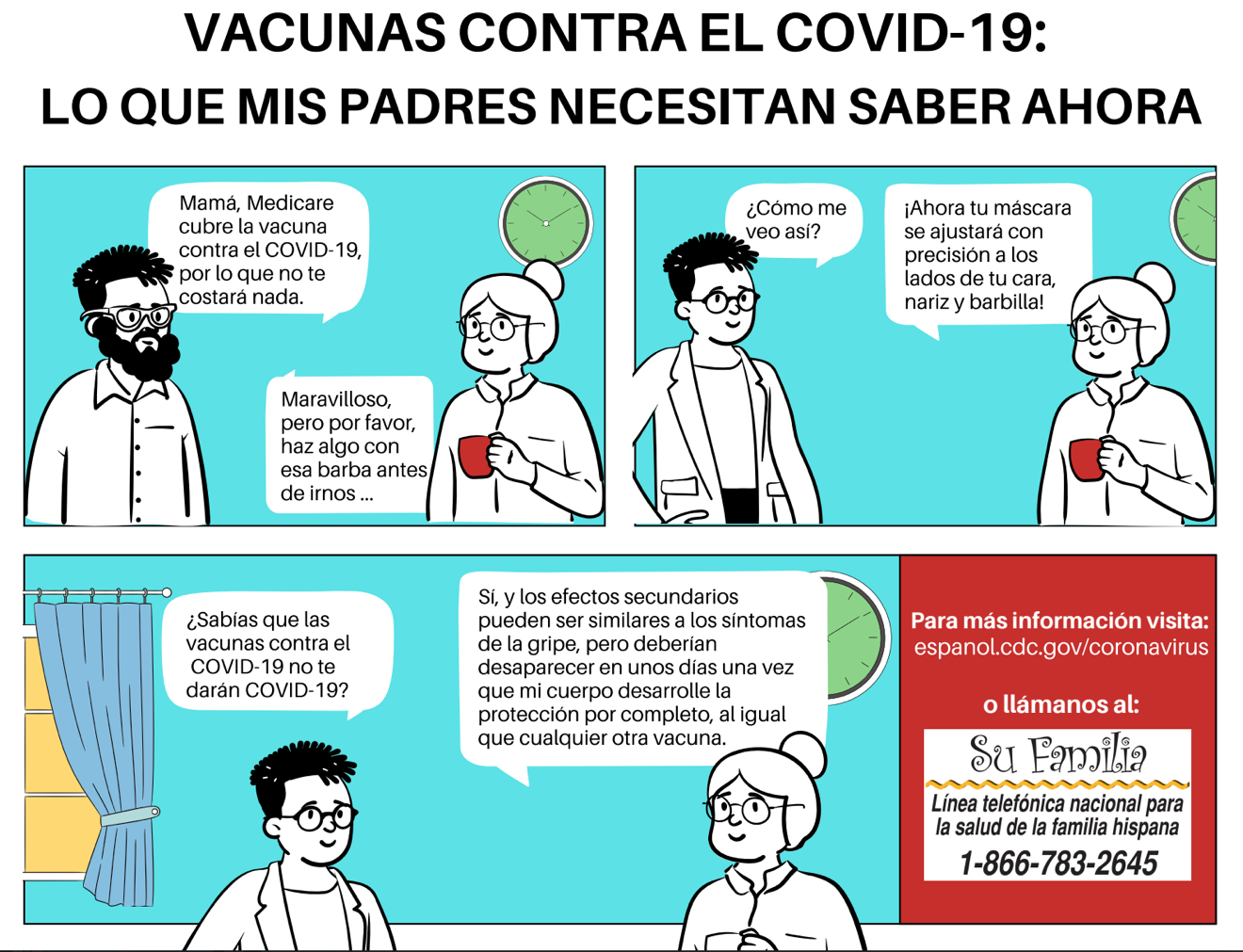
**

- ¿Qué es su primera reacción o qué son sus pensamientos iniciales cuando ves este mensaje? [**Indicaciones de seguimiento si se necesitan:** ¿Crees que este mensaje funcionaría para ti? Por ejemplo, ¿te convencería de vacunarte? ¿Por qué sí o por qué no?]
- ¿Cree que este mensaje sería efectivo para las personas de su comunidad? Piense en las person con que interactúas y con que vives. [**Indicaciones de seguimiento si se necesitan:** ¿Por qué o en que manera? ¿Cómo se podría mejorar?]

**
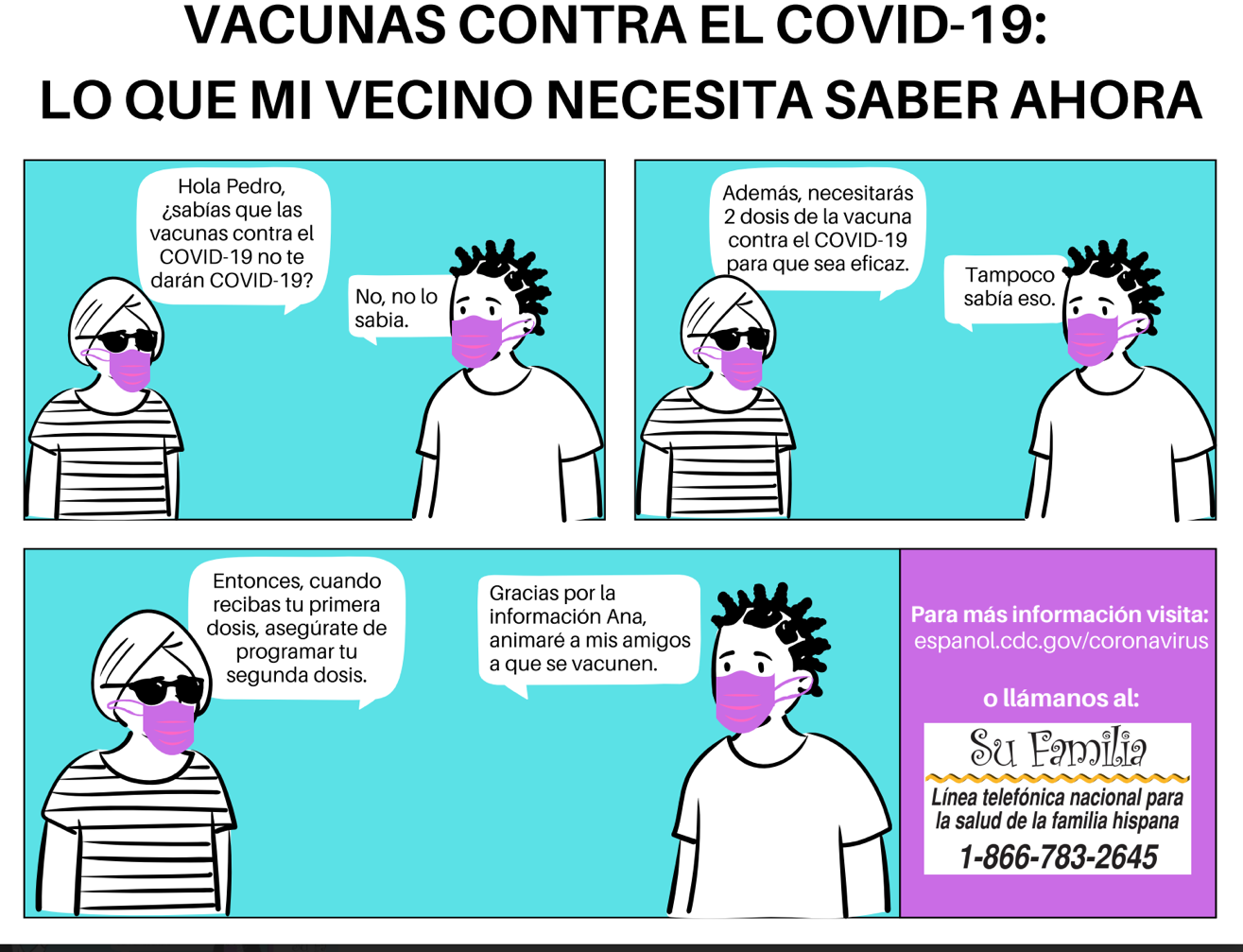
**

- ¿Qué es su primera reacción o qué son sus pensamientos iniciales cuando ves este mensaje? [**Indicaciones de seguimiento si se necesitan:** ¿Crees que este mensaje funcionaría para ti? Por ejemplo, ¿te convencería de vacunarte? ¿Por qué sí o por qué no?]
- ¿Cree que este mensaje sería efectivo para las personas de su comunidad? Piense en las person con que interactúas y con que vives. [**Indicaciones de seguimiento si se necesitan:** ¿Por qué o en que manera? ¿Cómo se podría mejorar?]

**Después del Grupo Focal**

- “Muchas gracias por compartir sus pensamientos y opiniones honestos relacionados con la vacuna COVID-19. Con esto concluye nuestra discusión de grupo focal”.
- “Ahora vamos a tomar un breve descanso de 10 minutos y luego les pediré a cada uno de ustedes que completen una encuesta posterior a la discusión. El tema es como nuestra discusión de hoy y está relacionado con sus opiniones y creencias sobre el COVID-19”.
- "La encuesta debería durar aproximadamente 30 minutos, pero algunos de ustedes necesitarán menos o más tiempo".
- “Una vez que haya terminado con la encuesta, entréguemela y le daré su tarjeta de regalo por participar hoy en nuestro estudio de investigación. Muchas gracias."

Appendix B

*Example Qualitative Survey Measures for Step 4 (Phase 2)*

**English**

Which language would you prefer to take this survey in? / ¿En qué idioma preferiría realizar esta encuesta?

- English
- Español

Are you aged 18 years or older?

- Yes
- No

Do you identify as Hispanic or Latino/Latina?

- Yes
- No (please write how you identify here):

Do you live in one of these counties?

Butte, Colusa, Fresno, Glenn, Kern, Kings, Madera, Merced, Placer, Sacramento, San Joaquin, Shasta, Stanislaus, Sutter, Tehama, Tulare, Yolo, Yuba.

- Yes
- No

**CONSENT TO PARTICIPATE IN A RESEARCH STUDY**

Study Title: Beliefs about COVID-19 Vaccines

**Investigator’s Name(s), Department(s), Email, Telephone Number(s):** Martin Hagger, Department of Psychological Sciences, mhagger@ucmerced.edu, (209)228-4400.

**PURPOSE**

You are being asked to participate in a research study. We hope to learn about peoples’ attitudes and beliefs about getting or not getting the COVID-19 vaccines.

**PROCEDURES**

If you decide to volunteer, you will be asked to complete an online survey on your own that will contain some simple questions about your attitudes and beliefs toward the COVID-19 vaccine. You will also be asked to provide some background demographic details. This information is not used to identify you in any way but rather it will tell us about the representation of the individuals participating in the study. Overall, the study will take approximately 10-15 minutes to complete.

**RISKS**

We anticipate no risks to you if you agree to participate in this study beyond the normal risk you would reasonably expect to experience as you go about your daily living.

**BENEFITS**

**It is possible that you will not benefit directly by participating in this study.** Although the knowledge and conclusions of this research project will not be of direct benefit to you, your involvement will provide valuable information on the attitudes and beliefs people hold toward the COVID-19 vaccine and help us in future campaigns about the vaccine. You participation may, therefore, benefit other people like you through a greater understanding of these processes.

**CONFIDENTIALITY**

The information you provide will be treated confidentially and all comments and responses are anonymous. Your responses in the focus group and questionnaire will form part of a larger data response set, which will initially be stored by audio recording and Qualtrics. Your responses will be recorded using a unique code identifier. Data will be password-protected and accessible only to members of the research team until all participant identifiers are removed from the data set. Participants’ data will not be identifiable in any publication or reporting. In the interest of researcher transparency, a strictly de-identified version of the research data will be prepared and made available on the online open data repository Open Science Framework (https://osf.io/). **Absolute confidentiality cannot be guaranteed, since research documents are not protected from subpoena.**

Your information collected as part of the research, even if identifiers are removed, will not be used or distributed for future research studies.

**RIGHT TO REFUSE OR WITHDRAW**

**Participation is completely voluntary. You may refuse to participate in this study. You may change your mind about being in the study and quit after the study has started.**

**QUESTIONS**

If you have any questions about this research project please contact Martin Hagger who will answer at (209)228-4400.

For questions about your rights while taking part in this study call the Office of Research at (209) 228-4805 or write to the Office of Research,5200 North Lake Rd, UC Merced, Merced, CA 95343. The Office of Research will inform the Institutional Review Board which is a group of people who review the research to protect your rights. If you have any complaints or concerns about this study, you may address them to Rose Scott, Chair of the IRB at (209) 228-4362, irbchair@ucmerced.edu.

**CONSENT**

**YOUR CONTINUATION TO THE SURVEY ON THE NEXT PAGE WILL INDICATE THAT YOU HAVE DECIDED TO VOLUNTEER AS A RESEARCH SUBJECT AND THAT YOU HAVE READ AND UNDERSTOOD THE INFORMATION PROVIDED ABOVE. PLEASE FEEL FREE TO PRINT AND KEEP A COPY OF THIS PAGE FOR YOUR RECORDS.**

Gender:

- Male
- Female
- Non-binary
- Prefer not to disclose
- Prefer to self-disclose:

Age (in years):

Postal code or zip code of residence:

Highest level of education achieved:

- Did not complete high school
- Completed high school
- Completed vocational school / trade school / AA degree / college certificate
- Completed an undergraduate college degree (B.A. or B.S.)
- Completed a postgraduate degree (Ph.D., M.A., M.S., MD, DDS, etc)

What is your main language spoken at home?

- English
- Spanish
- Other:

What is your preferred language for everyday written and oral communication?

- English
- Spanish
- Other:

Total family income in the last 12 months:

- Under $19,000
- $19,000 - $32,000
- $32,001 - $53,000
- $53,001 - $87,000
- $87,001 - $144,000
- More than $144,000
- Prefer not to disclose

Employment status:

- Employed full-time
- Employed part-time
- Employed in temporary or seasonal work
- Not employed, but looking for work
- Not employed, and not looking for work
- Not employed, unable to work due to disability or illness
- Retired
- Student
- Stay-at-home spouse/partner

What is your occupation or usual occupation (if currently unemployed)?

Does your occupation or usual occupation (if currently unemployed) involve direct contact with the general public outside of the home (e.g., customers, clients, patients, students)?

- Yes
- No

Have you received at least one dose of a COVID-19 vaccine?

- Yes
- No

Have you received the updated 2023-2024 COVID-19 vaccine?

- Yes
- No

The following questions are concerned with your views about the COVID-19 vaccine. There are no right or wrong responses; we are merely interested in your personal opinions.

Please take a few minutes to tell us what you think about **getting the COVID-19 vaccine (primary series or booster)**. In response to the questions below, please list the thoughts that come immediately to mind.

1. What do you see as the advantages of getting **a COVID-19 vaccine**?
2. What do you see as the disadvantages of getting **a COVID-19 vaccine**?
3. What else comes to mind when you think about getting **a COVID-19 vaccine**?
4. Please list the people who are important to you (e.g., your friends, doctor, coworkers, etc.) who might encourage you to get **a COVID-19 vaccine**.
5. Please list the people who are important to you (e.g., your friends, doctor, coworkers, etc.) who might discourage you from getting **a COVID-19 vaccine**.
6. Please list the individuals or groups that you know, and are important to you, that have gotten **a COVID-19 vaccine**.
7. Please list any factors or circumstances that would make it easy or enable you or someone in your community to get **a COVID-19 vaccine**.
8. Please list any factors or circumstances that would make it difficult or prevent you or someone in your community from getting **a COVID-19 vaccine**.

**Spanish**

Which language would you prefer to take this survey in? / ¿En qué idioma preferiría realizar esta encuesta?

- English
- Español

¿Tiene 18 años o más?

- Sí
- No

¿Usted se identifica como Hispano o Latino?

- Sí
- No (por favor, indique cómo se identifica):

¿Vive usted en uno de estos condados?

Butte, Colusa, Fresno, Glenn, Kern, Kings, Madera, Merced, Placer, Sacramento, San Joaquin, Shasta, Stanislaus, Sutter, Tehama, Tulare, Yolo, Yuba.

- Sí
- No

**CONSENTIMIENTO PARA PARTICIPAR EN UN ESTUDIO DE INVESTIGACIÓN**

Título del estudio: Creencias sobre las vacunas contra el COVID-19

**Nombre(s) del investigador, Departamento(s), correo electrónico, Número(s) de teléfono:** Martin Hagger, Departamento de Ciencias Psicológicas, mhagger@ucmerced.edu, (209)228-4400.

**OBJETIVO**

Se le está solicitando que participe en un estudio de investigación. Esperamos aprender sobre las actitudes y creencias de las personas sobre recibir o no las vacunas contra el COVID-19.

**PROCEDIMIENTOS**

Si decide ser voluntario, se le pedirá que complete una encuesta en línea por su cuenta que contendrá algunas preguntas simples sobre sus actitudes y creencias hacia la vacuna contra el COVID-19. También se le pedirá que proporcione algunos detalles demográficos generales. Esta información no se utiliza para identificarlo de ninguna manera, sino que nos informará sobre la representación de las personas que participan en el estudio. En general, el estudio tardará aproximadamente entre 1,5 y 2 horas en completarse.

**RIESGOS**

A. No anticipamos ningún riesgo para usted si acepta participar en este estudio más allá del riesgo normal que razonablemente esperaría experimentar en su vida diaria.

**BENEFICIOS**

**Es posible que usted no se beneficie directamente al participar en este estudio.** Aunque el conocimiento y las conclusiones de este proyecto de investigación no serán de beneficio directo para usted, su participación proporcionará información valiosa sobre las actitudes y creencias que las personas tienen hacia la vacuna COVID-19 y nos ayudará en futuras campañas sobre la vacuna. Por lo tanto, su participación puede beneficiar a otras personas como usted a través de una mayor comprensión de estos procesos.

**CONFIDENCIALIDAD**

La información que usted proporcione será tratada de manera confidencial y todos los comentarios y respuestas serán anónimos. Sus respuestas en el grupo focal y el cuestionario formarán parte de un conjunto de respuestas de datos más amplio, que inicialmente se almacenará mediante grabaciones de audio y Qualtrics. Sus respuestas se registrarán utilizando un código identificador único. Los datos estarán protegidos con contraseña y solo serán accesibles para los miembros del equipo de investigación hasta que todos los identificadores de los participantes se eliminen del conjunto de datos. Los datos de los participantes no serán identificables en ninguna publicación o informe. En interés de la transparencia de los investigadores, se preparará y se pondrá a disposición una versión estrictamente desidentificada de los datos de la investigación en el repositorio de datos en línea llamando Open Science Framework (https://osf.io/). **No se puede garantizar una confidencialidad absoluta, ya que los documentos de investigación no están protegidos contra citaciones legales.**

Su información recopilada como parte de la investigación, incluso si se eliminan los identificadores, no se utilizará ni se distribuirá para futuros estudios de investigación.

**DERECHO A RECHAZAR O RETIRAR**

**La participación es completamente voluntaria. Puede negarse a participar en este estudio. Usted puede cambiar de opinión sobre su participación en el estudio y retirarse después de que haya comenzado.**

**PREGUNTAS**

Si tiene alguna pregunta sobre este proyecto de investigación, por favor comuníquese con Martin Hagger que responderá al (209)228-4400.

Si tiene preguntas sobre sus derechos durante su participación en este estudio, llame a la Oficina de Investigación al (209) 228-4805 o escriba a la Oficina de Investigación, 5200 North Lake Rd, UC Merced, Merced, CA 95343. La Oficina de Investigación le informará la Institutional Review Board (IRB), que es un grupo de personas que revisan la investigación para proteger sus derechos. Si tiene alguna queja o inquietud sobre este estudio, puede dirigirla a Rose Scott, presidenta del IRB al (209) 228-4362, [irbchair@ucmerced.edu](mailto:irbchair@ucmerced.edu).

**CONSENTIMIENTO**

**SU FIRMA, A CONTINUACIÓN, INDICARÁ QUE HA DECIDIDO SER VOLUNTARIO COMO SUJETO DE INVESTIGACIÓN Y QUE HA LEÍDO Y ENTENDIDO LA INFORMACIÓN PROPORCIONADA ARRIBA. SE LE ENTREGARÁ UNA COPIA FIRMADA Y FECHADA DE ESTA FORMA PARA LLEVAR CONTIGO.**

Género:

- Hombre
- Mujer
- No binario
- Preferiría no revelar
- Yo preferiría revelar:

Edad (en años):

Código postal o código ZIP o lugar de residencia:

Nivel más alto de educación alcanzado:

- No completé la escuela secundaria
- Completé la escuela secundaria
- Completé una escuela de formación profesional / escuela de oficios o escuela técnica / título AA / certificado de universidad
- Completé un título universitario de pregrado (B.A. o B.S.)
- Completé un título de posgrado (Ph.D., M.A., M.S., MD, DDS, etc.)

¿Qué idioma hablas principalmente en casa?

- Inglés
- Español
- Otro:

¿Qué idioma prefieres para la comunicación escrita y oral?

- Inglés
- Español
- Otro:

Ingresos totales de la familia durante los 12 meses anteriores:

- Menos que $19,000
- $19,000 a $32,000
- $32,001 a $53,000
- $53,001 a $87,000
- $87,001 a $144,000
- Mas que $144,000
- Preferiría no revelar

Estado de empleo:

- Empleo a tiempo completo
- Empleo a tiempo parcial
- Empleo en trabjo que es temporal o estacional
- Sin empleo, pero buscando trabajo
- Sin empleo, y no buscando trabajo
- Sin empleo, y sin poder trabajar por discapacidad o enfermedad
- Retirado
- Estudiante
- Pareja/esposo/esposa que se quede en casa (por ejemplo, ama de casa)

¿Cuál es su trabajo o trabajo habitual (si actualmente está desempleado)?

¿Su trabajo o trabajo habitual (si actualmente está desempleado) implica contacto directo con personas del público general fuera de su hogar (por ejemplo, clientes, pacientes, estudiantes)?

- Sí
- No

¿Ha recibido al menos una vacuna contra el COVID-19?

- Sí
- No

¿Ha recibido la vacuna contra el COVID-19 que se actualizó en 2023-2024?

- Sí
- No

Las siguientes preguntas están relacionadas a su opinión sobre la vacuna COVID-19. No hay respuestas correctas o incorrectas; simplemente nos interesan sus opiniones personales.

Por favor tómese unos minutos para decirnos lo que piensas de **recibir la vacuna contra el COVID-19 (serie primaria o refuerzo)**. En respuesta a las siguientes preguntas, describa or explica los pensamientos que le vienen inmediatamente a la mente.

1. ¿Cuáles son las ventajas que considera al recibir **la** **vacuna contra el COVID-19**?
2. ¿Cuáles son las desventajas que considera al recibir **la** **vacuna contra el COVID-19**?
3. ¿Cuándo pienses recibir **la vacuna contra el COVID-19**, que se viene a la mente?
4. Por favor, haga una lista de las personas que son importante para usted (por ejemplo, tus amigos, doctor, compañeros de trabajo) y quien a lo mejor podrían animarte a recibir una **vacuna contra el COVID-19**.
5. Por favor, haga una lista de las personas que son importante para usted (por ejemplo, tus amigos, doctor, compañeros de trabajo) y quien a lo mejor podrían desanimarte a recibir una **vacuna contra el COVID-19**.
6. Por favor, indique las personas o grupos que conoces y son importantes para usted, y que han recibido **la vacuna contra el COVID-19**.
7. Por favor, indique o describa los factores o circunstancias que facilitarían o permitirían que usted o alguien en su comunidad reciba una **vacuna contra el COVID-19**.
8. Por favor, indique o describa los factores o circunstancias que dificultarían o impedirían que usted o alguien en su comunidad reciba una **vacuna contra el COVID-19**.

Appendix C

*Example Quantitative Survey Measures for Phase 3 (Step 5) (Illustrative Questions)*

**English**

**Your Opinions about Vaccines and COVID-19 Vaccines**

Thank you for participating in this study. The aim of the study is to develop an understanding of people’s beliefs about COVID-19 vaccines and vaccines in general.

There are many kinds of vaccines. When we talk about the vaccine for COVID-19, it is important for us to be clear what we mean:

- A COVID-19 *primary* vaccine refers to the first and second doses of the COVID-19 vaccine series that people get to immunize them against the virus. Together, these are known as the primary series of vaccines.
- Then there is also the COVID-19 *booster* vaccine, which refers to all doses of the vaccine you might receive after those first two doses or *primary* series of vaccines.

Keeping these definitions in mind, please respond to each of the statements below as they relate to YOU getting the COVID-19 vaccine that is *currently available* to you, or will be *likely be available* to you in future. By “available” we mean that you are eligible to receive the vaccine according to government vaccine recommendations approved by the FDA and CDC. There are no right or wrong answers. We are interested in your personal views

Your answers to the questions are confidential and will not be used for purposes other than the present research. All questions are voluntary. Answers to the questions are anonymous.

**VaCCS Scale**

Please respond to the following statements. Remember: when we talk about the **‘COVID-19 vaccine’**, we are referring to 1 dose of any updated 2023-2024 COVID-19 vaccine (Pfizer-BioNTech, Moderna or Novavax).

1. If I get the COVID-19 vaccine it will help to protect my family and friends against the coronavirus.

Strongly Disagree Somewhat Neither agree Somewhat Agree Strongly

disagree disagree nor disagree agree agree

1 2 3 4 5 6 7

2. The COVID-19 vaccine will help protect me from the coronavirus.

Strongly Disagree Somewhat Neither agree Somewhat Agree Strongly

disagree disagree nor disagree agree agree

1 2 3 4 5 6 7

3. The COVID-19 vaccine will stop the spread of the coronavirus.

Strongly Disagree Somewhat Neither agree Somewhat Agree Strongly

disagree disagree nor disagree agree agree

1 2 3 4 5 6 7

4. The COVID-19 vaccine is effective.

Strongly Disagree Somewhat Neither agree Somewhat Agree Strongly

disagree disagree nor disagree agree agree

1 2 3 4 5 6 7

5. The COVID-19 vaccine will reduce the severity of symptoms if I get the coronavirus.

Strongly Disagree Somewhat Neither agree Somewhat Agree Strongly

disagree disagree nor disagree agree agree

1 2 3 4 5 6 7

6. Getting the COVID-19 vaccine will help to get things back to normal.

Strongly Disagree Somewhat Neither agree Somewhat Agree Strongly

disagree disagree nor disagree agree agree

1 2 3 4 5 6 7

7. It is important to get the COVID-19 vaccine so that future outbreaks do not occur.

Strongly Disagree Somewhat Neither agree Somewhat Agree Strongly

disagree disagree nor disagree agree agree

1 2 3 4 5 6 7

8. Getting the COVID-19 vaccine is important for the health of others in my community.

Strongly Disagree Somewhat Neither agree Somewhat Agree Strongly

disagree disagree nor disagree agree agree

1 2 3 4 5 6 7

9. I trust the government to give me reliable information on the benefits and risks of the COVID-19 vaccine.

Strongly Disagree Somewhat Neither agree Somewhat Agree Strongly

disagree disagree nor disagree agree agree

1 2 3 4 5 6 7

10. I trust healthcare providers and health professionals to give me reliable information on the benefits and risks of the COVID-19 vaccine.

Strongly Disagree Somewhat Neither agree Somewhat Agree Strongly

disagree disagree nor disagree agree agree

1 2 3 4 5 6 7

11. I trust the government’s conclusions that the COVID-19 vaccine is safe.

Strongly Disagree Somewhat Neither agree Somewhat Agree Strongly

disagree disagree nor disagree agree agree

1 2 3 4 5 6 7

12. I trust scientists to give me reliable information on the benefits and risks of the COVID-19 vaccine.

Strongly Disagree Somewhat Neither agree Somewhat Agree Strongly

disagree disagree nor disagree agree agree

1 2 3 4 5 6 7

13. I trust scientists’ conclusions that the COVID-19 vaccine is safe.

Strongly Disagree Somewhat Neither agree Somewhat Agree Strongly

disagree disagree nor disagree agree agree

1 2 3 4 5 6 7

14. I trust healthcare providers’ and health professionals’ conclusions that the COVID-19 vaccine is safe.

Strongly Disagree Somewhat Neither agree Somewhat Agree Strongly

disagree disagree nor disagree agree agree

1 2 3 4 5 6 7

15. I trust vaccine manufacturers to give me reliable information on the benefits and risks of the COVID-19 vaccine.

Strongly Disagree Somewhat Neither agree Somewhat Agree Strongly

disagree disagree nor disagree agree agree

1 2 3 4 5 6 7

16. I am concerned about the side effects of the COVID-19 vaccine.

Strongly Disagree Somewhat Neither agree Somewhat Agree Strongly

disagree disagree nor disagree agree agree

1 2 3 4 5 6 7

17. I fear that the COVID-19 vaccine will cause side effects.

Strongly Disagree Somewhat Neither agree Somewhat Agree Strongly

disagree disagree nor disagree agree agree

1 2 3 4 5 6 7

18. I am worried about the safety of the COVID-19 vaccine.

Strongly Disagree Somewhat Neither agree Somewhat Agree Strongly

disagree disagree nor disagree agree agree

1 2 3 4 5 6 7

19. The COVID-19 vaccine can cause the coronavirus in some people.

Strongly Disagree Somewhat Neither agree Somewhat Agree Strongly

disagree disagree nor disagree agree agree

1 2 3 4 5 6 7

20. The COVID-19 vaccine can give you a serious case of the very same virus you’re trying to avoid.

Strongly Disagree Somewhat Neither agree Somewhat Agree Strongly

disagree disagree nor disagree agree agree

1 2 3 4 5 6 7

21. I can get the coronavirus from the COVID-19 vaccine.

Strongly Disagree Somewhat Neither agree Somewhat Agree Strongly

disagree disagree nor disagree agree agree

1 2 3 4 5 6 7

22. The COVID-19 vaccine safety data is often made up.

Strongly Disagree Somewhat Neither agree Somewhat Agree Strongly

disagree disagree nor disagree agree agree

1 2 3 4 5 6 7

23. People have been deceived about the safety of the COVID-19 vaccine.

Strongly Disagree Somewhat Neither agree Somewhat Agree Strongly

disagree disagree nor disagree agree agree

1 2 3 4 5 6 7

24. The COVID-19 vaccine is promoted mainly because of manufacturers’ profit.

Strongly Disagree Somewhat Neither agree Somewhat Agree Strongly

disagree disagree nor disagree agree agree

1 2 3 4 5 6 7

25. The main reason for promoting the COVID-19 vaccine is for drug companies to make money.

Strongly Disagree Somewhat Neither agree Somewhat Agree Strongly

disagree disagree nor disagree agree agree

1 2 3 4 5 6 7

26. I am opposed to the COVID-19 vaccine because it goes against freedom of choice.

Strongly Disagree Somewhat Neither agree Somewhat Agree Strongly

disagree disagree nor disagree agree agree

1 2 3 4 5 6 7

27. I am afraid of getting the COVID-19 vaccine.

Strongly Disagree Somewhat Neither agree Somewhat Agree Strongly

disagree disagree nor disagree agree agree

1 2 3 4 5 6 7

28. I am fearful about getting the COVID-19 vaccine.

Strongly Disagree Somewhat Neither agree Somewhat Agree Strongly

disagree disagree nor disagree agree agree

1 2 3 4 5 6 7

29. Getting the COVID-19 vaccine makes me feel anxious.

Strongly Disagree Somewhat Neither agree Somewhat Agree Strongly

disagree disagree nor disagree agree agree

1 2 3 4 5 6 7

30. The COVID-19 vaccine is too new so I should wait before deciding to get it.

Strongly Disagree Somewhat Neither agree Somewhat Agree Strongly

disagree disagree nor disagree agree agree

1 2 3 4 5 6 7

31. More time is needed to be able to fully investigate the true effects of the COVID-19 vaccine.

Strongly Disagree Somewhat Neither agree Somewhat Agree Strongly

disagree disagree nor disagree agree agree

1 2 3 4 5 6 7

32. I am afraid that the COVID-19 vaccine has not been successfully tested on enough people.

Strongly Disagree Somewhat Neither agree Somewhat Agree Strongly

disagree disagree nor disagree agree agree

1 2 3 4 5 6 7

33. I have access to all the information I need to make good decisions about getting the COVID-19 vaccine.

Strongly Disagree Somewhat Neither agree Somewhat Agree Strongly

disagree disagree nor disagree agree agree

1 2 3 4 5 6 7

34. Information about the COVID-19 vaccine is easy to understand.

Strongly Disagree Somewhat Neither agree Somewhat Agree Strongly

disagree disagree nor disagree agree agree

1 2 3 4 5 6 7

35. I don’t have enough information about the COVID-19 vaccine to decide.

Strongly Disagree Somewhat Neither agree Somewhat Agree Strongly

disagree disagree nor disagree agree agree

1 2 3 4 5 6 7

**Past Vaccination**

Please respond to each of the statements below. There are no right or wrong answers. We are interested in your personal experience.

1. Did you get the seasonal influenza vaccination in the prior year?

No Yes

1. 2

2. Have you ever refused or elected to forego a doctor-recommended vaccine?

No Yes

1. 2

3. When the seasonal influence vaccine is available each year I:

Never get it Always get it

1 2 3 4 5 6 7

**Intention**

Please respond to the following statements. Remember: when we talk about the **‘COVID-19 vaccine’**, we are referring to 1 dose of any updated 2023-2024 COVID-19 vaccine (Pfizer-BioNTech, Moderna or Novavax).

1. I intend to get the COVID-19 vaccine.

Strongly Disagree Somewhat Neither agree Somewhat Agree Strongly

disagree disagree nor disagree agree agree

1 2 3 4 5 6 7

2. I plan to get the COVID-19 vaccine.

Strongly Disagree Somewhat Neither agree Somewhat Agree Strongly

disagree disagree nor disagree agree agree

1 2 3 4 5 6 7

3. It is likely I will get the COVID-19 vaccine.

Strongly Disagree Somewhat Neither agree Somewhat Agree Strongly

disagree disagree nor disagree agree agree

1 2 3 4 5 6 7

**Attitudes**

Please respond to the following statements. Remember: when we talk about the **‘COVID-19 vaccine’**, we are referring to 1 dose of any updated 2023-2024 COVID-19 vaccine (Pfizer-BioNTech, Moderna or Novavax).

1. Getting the COVID-19 vaccine when it is offered to me would be:

Bad Good

1 2 3 4 5 6 7

2. Getting the COVID-19 vaccine when it is offered to me would be:

Worthless Valuable

1 2 3 4 5 6 7

3. Getting the COVID-19 vaccine when it is offered to me would be:

Harmful Beneficial

1 2 3 4 5 6 7

**Behavioral Beliefs**

Please respond to the following statements. 

1. If I were to get the updated COVID-19 vaccine, it would…

a. …protect me from COVID-19.

Strongly Disagree Somewhat Neither agree Somewhat Agree Strongly

disagree disagree nor disagree agree agree

1 2 3 4 5 6 7

b. …prevent me from getting sick.

Strongly Disagree Somewhat Neither agree Somewhat Agree Strongly

disagree disagree nor disagree agree agree

1 2 3 4 5 6 7

c. …prevent me from getting severely ill.

Strongly Disagree Somewhat Neither agree Somewhat Agree Strongly

disagree disagree nor disagree agree agree

1 2 3 4 5 6 7

d. …help me stay safe.

Strongly Disagree Somewhat Neither agree Somewhat Agree Strongly

disagree disagree nor disagree agree agree

1 2 3 4 5 6 7

e. …make me more employable.

Strongly Disagree Somewhat Neither agree Somewhat Agree Strongly

disagree disagree nor disagree agree agree

1 2 3 4 5 6 7

f. …give me peace of mind.

Strongly Disagree Somewhat Neither agree Somewhat Agree Strongly

disagree disagree nor disagree agree agree

1 2 3 4 5 6 7

g. …cause short-term side effects.

Strongly Disagree Somewhat Neither agree Somewhat Agree Strongly

disagree disagree nor disagree agree agree

1 2 3 4 5 6 7

h. …cause long-term health issues.

Strongly Disagree Somewhat Neither agree Somewhat Agree Strongly

disagree disagree nor disagree agree agree

1 2 3 4 5 6 7

i. …not be effective.

Strongly Disagree Somewhat Neither agree Somewhat Agree Strongly

disagree disagree nor disagree agree agree

1 2 3 4 5 6 7

j. …be unsafe.

Strongly Disagree Somewhat Neither agree Somewhat Agree Strongly

disagree disagree nor disagree agree agree

1 2 3 4 5 6 7

k. …be too inconvenient.

Strongly Disagree Somewhat Neither agree Somewhat Agree Strongly

disagree disagree nor disagree agree agree

1 2 3 4 5 6 7

**Subjective/Descriptive Norms**

Please respond to the following statements. Remember: when we talk about the **‘COVID-19 vaccine’**, we are referring to 1 dose of any updated 2023-2024 COVID-19 vaccine (Pfizer-BioNTech, Moderna or Novavax).

1. Most people who are important to me would approve of me getting the COVID-19 vaccine.

Strongly Disagree Somewhat Neither agree Somewhat Agree Strongly

disagree disagree nor disagree agree agree

1 2 3 4 5 6 7

2. Those people who are important to me think that I should get the COVID-19 vaccine.

Strongly Disagree Somewhat Neither agree Somewhat Agree Strongly

disagree disagree nor disagree agree agree

1 2 3 4 5 6 7

3. Of the people important to me, most would get the COVID-19 vaccine.

Strongly Disagree Somewhat Neither agree Somewhat Agree Strongly

disagree disagree nor disagree agree agree

1 2 3 4 5 6 7

**Moral Norms**

Please respond to the following statements.

1. It is the right thing to do to get the COVID-19 vaccine.

Strongly Disagree Somewhat Neither agree Somewhat Agree Strongly

disagree disagree nor disagree agree agree

1 2 3 4 5 6 7

2. It is morally responsible to get the COVID-19 vaccine.

Strongly Disagree Somewhat Neither agree Somewhat Agree Strongly

disagree disagree nor disagree agree agree

1 2 3 4 5 6 7

3. It is my moral obligation to get the COVID-19 vaccine.

Strongly Disagree Somewhat Neither agree Somewhat Agree Strongly

disagree disagree nor disagree agree agree

1 2 3 4 5 6 7

**Normative Beliefs**

1. The following people are likely to think I should get the COVID-19 vaccine when it is offered to me:

a. My family.

Strongly Disagree Somewhat Neither agree Somewhat Agree Strongly

disagree disagree nor disagree agree agree

1 2 3 4 5 6 7

b. My friends.

Strongly Disagree Somewhat Neither agree Somewhat Agree Strongly

disagree disagree nor disagree agree agree

1 2 3 4 5 6 7

c. Health professionals.

Strongly Disagree Somewhat Neither agree Somewhat Agree Strongly

disagree disagree nor disagree agree agree

1 2 3 4 5 6 7

d. My employer/coworkers.

Strongly Disagree Somewhat Neither agree Somewhat Agree Strongly

disagree disagree nor disagree agree agree

1 2 3 4 5 6 7

j. If my family and friends were thinking of getting the COVID-19 vaccine, I would:

Suggest they Strongly encourage

do not get it them to get it

1 2 3 4 5

**Perceived Behavioral Control**

Please respond to the following statements. Remember: when we talk about the **‘COVID-19 vaccine’**, we are referring to 1 dose of any updated 2023-2024 COVID-19 vaccine (Pfizer-BioNTech, Moderna or Novavax).

1. It is mostly up to me whether I get the COVID-19 vaccine when it is available to me.

Strongly Disagree Somewhat Neither agree Somewhat Agree Strongly

disagree disagree nor disagree agree agree

1 2 3 4 5 6 7

2. I am confident I can get the COVID-19 vaccine when it is available to me.

Strongly Disagree Somewhat Neither agree Somewhat Agree Strongly

disagree disagree nor disagree agree agree

1 2 3 4 5 6 7

3. I have complete control over whether I get the COVID-19 vaccine when it is available to me.

Strongly Disagree Somewhat Neither agree Somewhat Agree Strongly

disagree disagree nor disagree agree agree

1 2 3 4 5 6 7

**Control Beliefs**

1. How likely are the following to prevent or discourage you from getting the COVID-19 vaccine…

a. Lack of available vaccination clinics near me.

Strongly Disagree Somewhat Neither agree Somewhat Agree Strongly

disagree disagree nor disagree agree agree

1 2 3 4 5 6 7

b. Lack of transportation to a vaccination clinic.

Strongly Disagree Somewhat Neither agree Somewhat Agree Strongly

disagree disagree nor disagree agree agree

1 2 3 4 5 6 7

c. Incurring out-of-pocket cost.

Strongly Disagree Somewhat Neither agree Somewhat Agree Strongly

disagree disagree nor disagree agree agree

1 2 3 4 5 6 7

d. Lack of insurance.

Strongly Disagree Somewhat Neither agree Somewhat Agree Strongly

disagree disagree nor disagree agree agree

1 2 3 4 5 6 7

e. Concern over side-effects.

Strongly Disagree Somewhat Neither agree Somewhat Agree Strongly

disagree disagree nor disagree agree agree

1 2 3 4 5 6 7

f. Concern over the vaccine causing health issues.

Strongly Disagree Somewhat Neither agree Somewhat Agree Strongly

disagree disagree nor disagree agree agree

1 2 3 4 5 6 7

g. Concern that the vaccine is poison.

Strongly Disagree Somewhat Neither agree Somewhat Agree Strongly

disagree disagree nor disagree agree agree

1 2 3 4 5 6 7

h. Negative beliefs about the vaccine.

Strongly Disagree Somewhat Neither agree Somewhat Agree Strongly

disagree disagree nor disagree agree agree

1 2 3 4 5 6 7

i. Lack of information about the vaccine.

Strongly Disagree Somewhat Neither agree Somewhat Agree Strongly

disagree disagree nor disagree agree agree

1 2 3 4 5 6 7

**Risk Perceptions**

Please respond to the following statements. Remember: when we talk about the **‘COVID-19 vaccine’**, we are referring to 1 dose of any updated 2023-2024 COVID-19 vaccine (Pfizer-BioNTech, Moderna or Novavax).

1. It would be risky for me to get the COVID-19 vaccine.

Strongly Disagree Somewhat Neither agree Somewhat Agree Strongly

disagree disagree nor disagree agree agree

1 2 3 4 5 6 7

2. If I got the COVID-19 vaccine there would be risk involved.

Strongly Disagree Somewhat Neither agree Somewhat Agree Strongly

disagree disagree nor disagree agree agree

1 2 3 4 5 6 7

**Vaccine Hesitancy**

Please respond to the following statements.

1. I would describe myself as:

Anti-vaccination Eager to get the

for COVID-19 COVID-19 vaccine

1 2 3 4 5

2. Getting the COVID-19 vaccine is:

Really Really

not important important

1 2 3 4 5

3. Overall, how hesitant are you about getting the COVID-19 vaccine?

Not at all Very much

1 2 3 4 5

**Knowledge About Vaccines**

Please respond to the following statements.

1. Without broadly applied vaccine programs, smallpox would still exist.

Yes No

1 2

2. The efficacy of vaccines has been proven.

Yes No

1 2

3. Children would be more resistant if they were not always vaccinated against all diseases.

Yes No

1 2

4. Diseases like autism, multiple sclerosis, and diabetes might be triggered through vaccinations.

Yes No

1 2

5. The immune system of children is not overloaded with many vaccinations.

Yes No

1 2

6. Many vaccinations are administered too early, so that the body’s own immune system has no possibility to develop.

Yes No

1 2

7. The doses of the chemicals used in the vaccines are not dangerous for humans.

Yes No

1 2

8. Vaccinations increase the occurrence of allergies.

Yes No

1 2

**Vaccine Confidence**

Please respond to the following statements.

1. I am completely confident that vaccines are safe.

Strongly Somewhat Neither agree Somewhat Strongly agree

disagree disagree nor disagree agree

1 2 3 4 5

2. Vaccination is unnecessary because vaccine preventable diseases are not common anymore.

Strongly Somewhat Neither agree Somewhat Strongly agree

disagree disagree nor disagree agree

1 2 3 4 5

3. Everyday stress prevents me from getting vaccinated.

Strongly Somewhat Neither agree Somewhat Strongly agree

disagree disagree nor disagree agree

1 2 3 4 5

4. When I think about getting vaccinated, I weigh benefits and risks to make the best decision possible.

Strongly Somewhat Neither agree Somewhat Strongly agree

disagree disagree nor disagree agree

1 2 3 4 5

5. When everyone is vaccinated, I don’t have to get vaccinated, too.

Strongly Somewhat Neither agree Somewhat Strongly agree

disagree disagree nor disagree agree

1 2 3 4 5

**Conspiracy Beliefs**

Please respond to the following statements.

1. COVID-19 is intentionally presented as dangerous in order to mislead the public.

Strongly Disagree Somewhat Neither agree Somewhat Agree Strongly

disagree disagree nor disagree agree agree

1 2 3 4 5 6 7

2. Experts intentionally mislead us for their own benefit, even though COVID-19 is no worse than the flu.

Strongly Disagree Somewhat Neither agree Somewhat Agree Strongly

disagree disagree nor disagree agree agree

1 2 3 4 5 6 7

3. We should believe experts when they say that COVID-19 is dangerous.

Strongly Disagree Somewhat Neither agree Somewhat Agree Strongly

disagree disagree nor disagree agree agree

1 2 3 4 5 6 7

4. Dark forces want to use COVID-19 to rule the world.

Strongly Disagree Somewhat Neither agree Somewhat Agree Strongly

disagree disagree nor disagree agree agree

1 2 3 4 5 6 7

5. COVID-19 was intentionally brought into the world to reduce the population.

Strongly Disagree Somewhat Neither agree Somewhat Agree Strongly

disagree disagree nor disagree agree agree

1 2 3 4 5 6 7

6. I think it’s nonsense that the virus was created in a laboratory.

Strongly Disagree Somewhat Neither agree Somewhat Agree Strongly

disagree disagree nor disagree agree agree

1 2 3 4 5 6 7

7. Overall, I would describe my attitude towards getting the COVID-19 vaccine as:

Against it Very keen

1 2 3 4 5

**Spanish**

**Sus opiniónes sobre las vacunas y las vacunas contra el COVID-19**

Gracias por participar en este estudio. El objetivo del estudio es desarrollar una comprensión de las creencias de las personas sobre las vacunas COVID-19 y las vacunas en general.

Hay muchos tipos de vacunas. Cuando hablamos de la vacuna para el COVID-19, es importante que tengamos claro a qué nos referimos:

- Una vacuna primaria contra el COVID-19 se refiere a las primeras y segundas dosis de la serie de vacunas contra el COVID-19 que las personas reciben para inmunizarse contra el virus. Juntas, se conocen como la serie primaria de vacunas.
- Luego también está la vacuna de refuerzo contra el COVID-19, que se refiere a todas las dosis de la vacuna que podrías recibir después de esas primeras dos dosis o serie primaria de vacunas.

Teniendo en cuenta estas definiciones, responda a cada una de las afirmaciones a continuación en lo que se refiere a que USTED reciba la vacuna contra el COVID-19, es decir,*actualmente disponible*para ti, o será*probablemente esté disponible* para usted en el futuro. Por "disponible" queremos decir que usted es elegible para recibir la vacuna de acuerdo con las recomendaciones gubernamentales sobre vacunas aprobadas por la FDA y los CDC. No hay respuestas correctas o incorrectas. Nos interesa su opinión personal.

Sus respuestas a las preguntas son confidenciales y no se utilizarán para otros fines que no sean los de la presente investigación. Todas las preguntas son voluntarias. Las respuestas a las preguntas son anónimas.

**VaCCS Scale**

Por favor responda a las siguientes afirmaciones. Recordar: cuando hablamos de la **'vacuna contra la COVID-19'**, nos referimos a 1 dosis de cualquier vacuna contra la COVID-19 (Pfizer-BioNTech, Moderna, o Novavax) que se actualizó en 2023-2024.

1. Si recibo la vacuna contra el COVID-19, ayudaré a proteger a mi familia y amigos contra el coronavirus.

Muy en Discrepar Algo en Ni de acuerdo Parcialmente Aceptar Totalmente

desacuerdo desacuerdo ni desacuerdo de acuerdo de acuerdo

1 2 3 4 5 6 7

2. La vacuna COVID-19 me ayudará a protegerme del coronavirus.

Muy en Discrepar Algo en Ni de acuerdo Parcialmente Aceptar Totalmente

desacuerdo desacuerdo ni desacuerdo de acuerdo de acuerdo

1 2 3 4 5 6 7

3. La vacuna COVID-19 detendrá la propagación del coronavirus.

Muy en Discrepar Algo en Ni de acuerdo Parcialmente Aceptar Totalmente

desacuerdo desacuerdo ni desacuerdo de acuerdo de acuerdo

1 2 3 4 5 6 7

4. La vacuna COVID-19 es eficaz.

Muy en Discrepar Algo en Ni de acuerdo Parcialmente Aceptar Totalmente

desacuerdo desacuerdo ni desacuerdo de acuerdo de acuerdo

1 2 3 4 5 6 7

5. La vacuna contra el COVID-19 reducirá la gravedad de los síntomas si contraigo el coronavirus.

Muy en Discrepar Algo en Ni de acuerdo Parcialmente Aceptar Totalmente

desacuerdo desacuerdo ni desacuerdo de acuerdo de acuerdo

1 2 3 4 5 6 7

6. Recibir la vacuna COVID-19 ayudará a que todo vuelva a la normalidad.

Muy en Discrepar Algo en Ni de acuerdo Parcialmente Aceptar Totalmente

desacuerdo desacuerdo ni desacuerdo de acuerdo de acuerdo

1 2 3 4 5 6 7

7. Es importante vacunarse contra el COVID-19 para que no se produzcan brotes futuros.

Muy en Discrepar Algo en Ni de acuerdo Parcialmente Aceptar Totalmente

desacuerdo desacuerdo ni desacuerdo de acuerdo de acuerdo

1 2 3 4 5 6 7

8. Recibir la vacuna COVID-19 es importante para la salud de otras personas en mi comunidad.

Muy en Discrepar Algo en Ni de acuerdo Parcialmente Aceptar Totalmente

desacuerdo desacuerdo ni desacuerdo de acuerdo de acuerdo

1 2 3 4 5 6 7

9. Confío en que el gobierno me brinde información confiable sobre los beneficios y riesgos de la vacuna contra el COVID-19.

Muy en Discrepar Algo en Ni de acuerdo Parcialmente Aceptar Totalmente

desacuerdo desacuerdo ni desacuerdo de acuerdo de acuerdo

1 2 3 4 5 6 7

10. Confío en que los proveedores de atención médica y los profesionales de la salud me brinden información sobre los beneficios y riesgos de la vacuna contra el COVID-19.

Muy en Discrepar Algo en Ni de acuerdo Parcialmente Aceptar Totalmente

desacuerdo desacuerdo ni desacuerdo de acuerdo de acuerdo

1 2 3 4 5 6 7

11. Yo confío en las conclusiones del gobierno de que la vacuna contra el COVID-19 es segura.

Muy en Discrepar Algo en Ni de acuerdo Parcialmente Aceptar Totalmente

desacuerdo desacuerdo ni desacuerdo de acuerdo de acuerdo

1 2 3 4 5 6 7

12. Confío en que los cientificos me brinden información confiable sobre los beneficios y riesgos de la vacuna contra el COVID-19.

Muy en Discrepar Algo en Ni de acuerdo Parcialmente Aceptar Totalmente

desacuerdo desacuerdo ni desacuerdo de acuerdo de acuerdo

1 2 3 4 5 6 7

13. Yo confío en las conclusiones del los cientificos de que la vacuna contra el COVID-19 es segura.

Muy en Discrepar Algo en Ni de acuerdo Parcialmente Aceptar Totalmente

desacuerdo desacuerdo ni desacuerdo de acuerdo de acuerdo

1 2 3 4 5 6 7

14. Yo confío en las conclusiones de los proveedores de atención médica y los profesionales de la salud de que las vacuna contra el COVID-19 es segura.

Muy en Discrepar Algo en Ni de acuerdo Parcialmente Aceptar Totalmente

desacuerdo desacuerdo ni desacuerdo de acuerdo de acuerdo

1 2 3 4 5 6 7

15. Confío en que los fabricantes de vacunas me brinden información confiable sobre los beneficios y riesgos de la vacuna contra el COVID-19.

Muy en Discrepar Algo en Ni de acuerdo Parcialmente Aceptar Totalmente

desacuerdo desacuerdo ni desacuerdo de acuerdo de acuerdo

1 2 3 4 5 6 7

16. Me preocupan los efectos secundarios de la vacuna contra el COVID-19.

Muy en Discrepar Algo en Ni de acuerdo Parcialmente Aceptar Totalmente

desacuerdo desacuerdo ni desacuerdo de acuerdo de acuerdo

1 2 3 4 5 6 7

17. Temo que la vacuna contra el COVID-19 cause efectos secundarios.

Muy en Discrepar Algo en Ni de acuerdo Parcialmente Aceptar Totalmente

desacuerdo desacuerdo ni desacuerdo de acuerdo de acuerdo

1 2 3 4 5 6 7

18. Me preocupa la seguridad de la vacuna contra el COVID-19.

Muy en Discrepar Algo en Ni de acuerdo Parcialmente Aceptar Totalmente

desacuerdo desacuerdo ni desacuerdo de acuerdo de acuerdo

1 2 3 4 5 6 7

19. La vacuna COVID-19 puede causar el coronavirus en algunas personas.

Muy en Discrepar Algo en Ni de acuerdo Parcialmente Aceptar Totalmente

desacuerdo desacuerdo ni desacuerdo de acuerdo de acuerdo

1 2 3 4 5 6 7

20. La vacuna contra el COVID-19 puede provocarle un caso grave del mismo virus que está tratando de evitar.

Muy en Discrepar Algo en Ni de acuerdo Parcialmente Aceptar Totalmente

desacuerdo desacuerdo ni desacuerdo de acuerdo de acuerdo

1 2 3 4 5 6 7

21. Puedo contraer el coronavirus mediante la vacuna COVID-19.

Muy en Discrepar Algo en Ni de acuerdo Parcialmente Aceptar Totalmente

desacuerdo desacuerdo ni desacuerdo de acuerdo de acuerdo

1 2 3 4 5 6 7

22. Los datos sobre la seguridad de la vacuna contra el COVID-19 suelen ser inventados.

Muy en Discrepar Algo en Ni de acuerdo Parcialmente Aceptar Totalmente

desacuerdo desacuerdo ni desacuerdo de acuerdo de acuerdo

1 2 3 4 5 6 7

23. Se ha engañado a la gente sobre la seguridad de la vacuna contra el COVID-19.

Muy en Discrepar Algo en Ni de acuerdo Parcialmente Aceptar Totalmente

desacuerdo desacuerdo ni desacuerdo de acuerdo de acuerdo

1 2 3 4 5 6 7

24. La vacuna contra el COVID-19 se promociona principalmente por las ganancias de los fabricantes.

Muy en Discrepar Algo en Ni de acuerdo Parcialmente Aceptar Totalmente

desacuerdo desacuerdo ni desacuerdo de acuerdo de acuerdo

1 2 3 4 5 6 7

25. La razón principal par promover la vacuna contra el COVID-19 es que las compañías farmacéuticas ganen dinero.

Muy en Discrepar Algo en Ni de acuerdo Parcialmente Aceptar Totalmente

desacuerdo desacuerdo ni desacuerdo de acuerdo de acuerdo

1 2 3 4 5 6 7

26. Me opongo a la vacuna contra el COVID-19 porque va en contra de la libertad de elección.

Muy en Discrepar Algo en Ni de acuerdo Parcialmente Aceptar Totalmente

desacuerdo desacuerdo ni desacuerdo de acuerdo de acuerdo

1 2 3 4 5 6 7

27. Tengo miedo de recibir la vacuna COVID-19.

Muy en Discrepar Algo en Ni de acuerdo Parcialmente Aceptar Totalmente

desacuerdo desacuerdo ni desacuerdo de acuerdo de acuerdo

1 2 3 4 5 6 7

28. Tengo miedo de recibir la vacuna contra el COVID-19.

Muy en Discrepar Algo en Ni de acuerdo Parcialmente Aceptar Totalmente

desacuerdo desacuerdo ni desacuerdo de acuerdo de acuerdo

1 2 3 4 5 6 7

29. Recibir la vacuna COVID-19 me produce ansiedad.

Muy en Discrepar Algo en Ni de acuerdo Parcialmente Aceptar Totalmente

desacuerdo desacuerdo ni desacuerdo de acuerdo de acuerdo

1 2 3 4 5 6 7

30. La vacuna contra el COVID-19 es demasiado nueva, por lo que debo esperar antes de decidirme a recibirla.

Muy en Discrepar Algo en Ni de acuerdo Parcialmente Aceptar Totalmente

desacuerdo desacuerdo ni desacuerdo de acuerdo de acuerdo

1 2 3 4 5 6 7

31. Se necesita más tiempo para poder investigar completamente los verdaderos efectos de la vacuna COVID-19.

Muy en Discrepar Algo en Ni de acuerdo Parcialmente Aceptar Totalmente

desacuerdo desacuerdo ni desacuerdo de acuerdo de acuerdo

1 2 3 4 5 6 7

32. Me temo que la vacuna contra la COVID-19 no se ha probado con éxito en un número suficiente de personas.

Muy en Discrepar Algo en Ni de acuerdo Parcialmente Aceptar Totalmente

desacuerdo desacuerdo ni desacuerdo de acuerdo de acuerdo

1 2 3 4 5 6 7

33. Tengo acceso a toda la información que necesito para tomar buenas decisiones sobre recibir la vacuna contra el COVID-19.

Muy en Discrepar Algo en Ni de acuerdo Parcialmente Aceptar Totalmente

desacuerdo desacuerdo ni desacuerdo de acuerdo de acuerdo

1 2 3 4 5 6 7

34. La información sobre la vacuna COVID-19 es fácil de entender.

Muy en Discrepar Algo en Ni de acuerdo Parcialmente Aceptar Totalmente

desacuerdo desacuerdo ni desacuerdo de acuerdo de acuerdo

1 2 3 4 5 6 7

35. No tengo suficiente información sobre la vacuna COVID-19 para decidir.

Muy en Discrepar Algo en Ni de acuerdo Parcialmente Aceptar Totalmente

desacuerdo desacuerdo ni desacuerdo de acuerdo de acuerdo

1 2 3 4 5 6 7

**Past Vaccination**

Por favor responda a cada una de las siguientes afirmaciones. No hay respuestas correctas o incorrectas. Nos interesa tu experiencia personal.

1. ¿Recibió la vacuna contra la influenza estacional el año anterior?

No Sí

1 2

2. ¿Alguna vez se ha negado o ha elegido renunciar a una vacuna recomendada por un médico?

No Sí

1 2

3. Cuando la vacuna contra la influenza estacional esté disponible cada año:

Nunca lo entiendas Siempre la recibo

1 2 3 4 5 6 7

**Intention**

Por favor responda a las siguientes afirmaciones. Recordar: cuando hablamos de la **'vacuna contra la COVID-19'**, nos referimos a 1 dosis de cualquier vacuna contra la COVID-19 (Pfizer-BioNTech, Moderna, o Novavax) que se actualizó en 2023-2024.

1. Tengo la intención de recibir la vacuna COVID-19.

Muy en Discrepar Algo en Ni de acuerdo Parcialmente Aceptar Totalmente

desacuerdo desacuerdo ni desacuerdo de acuerdo de acuerdo

1 2 3 4 5 6 7

2. Planeo recibir la vacuna COVID-19.

Muy en Discrepar Algo en Ni de acuerdo Parcialmente Aceptar Totalmente

desacuerdo desacuerdo ni desacuerdo de acuerdo de acuerdo

1 2 3 4 5 6 7

3. Es probable que reciba la vacuna contra el COVID-19.

Muy en Discrepar Algo en Ni de acuerdo Parcialmente Aceptar Totalmente

desacuerdo desacuerdo ni desacuerdo de acuerdo de acuerdo

1 2 3 4 5 6 7

**Attitudes**

Por favor responda a las siguientes afirmaciones. Recordar: cuando hablamos de la **'vacuna contra la COVID-19'**, nos referimos a 1 dosis de cualquier vacuna contra la COVID-19 (Pfizer-BioNTech, Moderna, o Novavax) que se actualizó en 2023-2024.

1. Recibir la vacuna contra el COVID-19 sería:

Mal Bueno

1 2 3 4 5 6 7

2. Recibir la vacuna contra el COVID-19 sería:

Inútil Valioso

1 2 3 4 5 6 7

3. Recibir la vacuna contra el COVID-19 sería:

Dañino Beneficioso

1 2 3 4 5 6 7

**Behavioral Beliefs**

Por favor responda a las siguientes afirmaciones.

Si recibiera la vacuna contra el COVID-19 más reciente (actualizada en 2023-2024), sería...

a. ...me protegería del COVID-19.

Muy en Discrepar Algo en Ni de acuerdo Parcialmente Aceptar Totalmente

desacuerdo desacuerdo ni desacuerdo de acuerdo de acuerdo

1 2 3 4 5 6 7

b. ...evitaría que me enfermara.

Muy en Discrepar Algo en Ni de acuerdo Parcialmente Aceptar Totalmente

desacuerdo desacuerdo ni desacuerdo de acuerdo de acuerdo

1 2 3 4 5 6 7

c. ...evitaría que me enferemara gravemente.

Muy en Discrepar Algo en Ni de acuerdo Parcialmente Aceptar Totalmente

desacuerdo desacuerdo ni desacuerdo de acuerdo de acuerdo

1 2 3 4 5 6 7

d. ...me ayudaría a mantenerme seguro.

Muy en Discrepar Algo en Ni de acuerdo Parcialmente Aceptar Totalmente

desacuerdo desacuerdo ni desacuerdo de acuerdo de acuerdo

1 2 3 4 5 6 7

e. …me haría más empleable.

Muy en Discrepar Algo en Ni de acuerdo Parcialmente Aceptar Totalmente

desacuerdo desacuerdo ni desacuerdo de acuerdo de acuerdo

1 2 3 4 5 6 7

f. ...me daría tranquilidad.

Muy en Discrepar Algo en Ni de acuerdo Parcialmente Aceptar Totalmente

desacuerdo desacuerdo ni desacuerdo de acuerdo de acuerdo

1 2 3 4 5 6 7

g. ...causaría efectos secundarios a corto plazo.

Muy en Discrepar Algo en Ni de acuerdo Parcialmente Aceptar Totalmente

desacuerdo desacuerdo ni desacuerdo de acuerdo de acuerdo

1 2 3 4 5 6 7

h. ...causaría efectos secundarios a largo plazo.

Muy en Discrepar Algo en Ni de acuerdo Parcialmente Aceptar Totalmente

desacuerdo desacuerdo ni desacuerdo de acuerdo de acuerdo

1 2 3 4 5 6 7

i. ...no sería efectiva.

Muy en Discrepar Algo en Ni de acuerdo Parcialmente Aceptar Totalmente

desacuerdo desacuerdo ni desacuerdo de acuerdo de acuerdo

1 2 3 4 5 6 7

j. ...no sería segura.

Muy en Discrepar Algo en Ni de acuerdo Parcialmente Aceptar Totalmente

desacuerdo desacuerdo ni desacuerdo de acuerdo de acuerdo

1 2 3 4 5 6 7

k. …sería demasiado inconviente.

Muy en Discrepar Algo en Ni de acuerdo Parcialmente Aceptar Totalmente

desacuerdo desacuerdo ni desacuerdo de acuerdo de acuerdo

1 2 3 4 5 6 7

**Subjective/Descriptive Norms**

Por favor responda a las siguientes afirmaciones. Recordar: cuando hablamos de la **'vacuna contra la COVID-19'**, nos referimos a 1 dosis de cualquier vacuna contra la COVID-19 (Pfizer-BioNTech, Moderna, o Novavax) que se actualizó en 2023-2024.

1. La mayoría de las personas que son importantes para mí aprobarían que me pusiera la vacuna contra el COVID-19.

Muy en Discrepar Algo en Ni de acuerdo Parcialmente Aceptar Totalmente

desacuerdo desacuerdo ni desacuerdo de acuerdo de acuerdo

1 2 3 4 5 6 7

2. Las personas que son importantes para mí piensan que debería recibir la vacuna contra el COVID-19.

Muy en Discrepar Algo en Ni de acuerdo Parcialmente Aceptar Totalmente

desacuerdo desacuerdo ni desacuerdo de acuerdo de acuerdo

1 2 3 4 5 6 7

3. De las personas importantes para mí, la mayoría recibiría la vacuna contra el COVID-19.

Muy en Discrepar Algo en Ni de acuerdo Parcialmente Aceptar Totalmente

desacuerdo desacuerdo ni desacuerdo de acuerdo de acuerdo

1 2 3 4 5 6 7

**Moral Norms**

Por favor responda a cada una de las siguientes afirmaciones.

1. Es lo correcto recibir la vacuna contra el COVID-19.

Muy en Discrepar Algo en Ni de acuerdo Parcialmente Aceptar Totalmente

desacuerdo desacuerdo ni desacuerdo de acuerdo de acuerdo

1 2 3 4 5 6 7

2. Es moralmente responsable recibir la vacuna contra el COVID-19.

Muy en Discrepar Algo en Ni de acuerdo Parcialmente Aceptar Totalmente

desacuerdo desacuerdo ni desacuerdo de acuerdo de acuerdo

1 2 3 4 5 6 7

3. Es mi obligación moral recibir la vacuna contra el COVID-19.

Muy en Discrepar Algo en Ni de acuerdo Parcialmente Aceptar Totalmente

desacuerdo desacuerdo ni desacuerdo de acuerdo de acuerdo

1 2 3 4 5 6 7

**Normative Beliefs**

1. Es probable que las siguientes personas piensen que debería vacunarme contra el COVID-19:

a. Mi familia.

Muy en Discrepar Algo en Ni de acuerdo Parcialmente Aceptar Totalmente

desacuerdo desacuerdo ni desacuerdo de acuerdo de acuerdo

1 2 3 4 5 6 7

b. Mis amigos.

Muy en Discrepar Algo en Ni de acuerdo Parcialmente Aceptar Totalmente

desacuerdo desacuerdo ni desacuerdo de acuerdo de acuerdo

1 2 3 4 5 6 7

c. Profesionales de la salud.

Muy en Discrepar Algo en Ni de acuerdo Parcialmente Aceptar Totalmente

desacuerdo desacuerdo ni desacuerdo de acuerdo de acuerdo

1 2 3 4 5 6 7

d. Mi empleador o mis compañeros de trabajo.

Muy en Discrepar Algo en Ni de acuerdo Parcialmente Aceptar Totalmente

desacuerdo desacuerdo ni desacuerdo de acuerdo de acuerdo

1 2 3 4 5 6 7

j. Si mi familia y amigos estuvieran pensando en vacunarse contra el COVID-19, yo:

Sugeriría que no Les animaría fuertemente a

lo hagan que lo hagan

1 2 3 4 5

**Perceived Behavioral Control**

Por favor responda a las siguientes afirmaciones. Recordar: cuando hablamos de la **'vacuna contra la COVID-19'**, nos referimos a 1 dosis de cualquier vacuna contra la COVID-19 (Pfizer-BioNTech, Moderna, o Novavax) que se actualizó en 2023-2024.

1. Depende principalmente de mí recibir la vacuna contra el COVID-19.

Muy en Discrepar Algo en Ni de acuerdo Parcialmente Aceptar Totalmente

desacuerdo desacuerdo ni desacuerdo de acuerdo de acuerdo

1 2 3 4 5 6 7

2. Estoy seguro de que podré recibir la vacuna contra el COVID-19.

Muy en Discrepar Algo en Ni de acuerdo Parcialmente Aceptar Totalmente

desacuerdo desacuerdo ni desacuerdo de acuerdo de acuerdo

1 2 3 4 5 6 7

3. Tengo control total sobre si recibiré la vacuna contra el COVID-19.

Muy en Discrepar Algo en Ni de acuerdo Parcialmente Aceptar Totalmente

desacuerdo desacuerdo ni desacuerdo de acuerdo de acuerdo

1 2 3 4 5 6 7

**Control Beliefs**

1. ¿Qué tan probable es que los siguientes le impidan o desanimen a vacunarse sobre el COVID-19?

a. Falta de clínicas de vacunación disponibles cerca de mí.

Muy en Discrepar Algo en Ni de acuerdo Parcialmente Aceptar Totalmente

desacuerdo desacuerdo ni desacuerdo de acuerdo de acuerdo

1 2 3 4 5 6 7

b. Falta de transporte a una clínica de vacunación.

Muy en Discrepar Algo en Ni de acuerdo Parcialmente Aceptar Totalmente

desacuerdo desacuerdo ni desacuerdo de acuerdo de acuerdo

1 2 3 4 5 6 7

c. Incurrir en costos de bolsillo propio.

Muy en Discrepar Algo en Ni de acuerdo Parcialmente Aceptar Totalmente

desacuerdo desacuerdo ni desacuerdo de acuerdo de acuerdo

1 2 3 4 5 6 7

d. Falta de serguro médico.

Muy en Discrepar Algo en Ni de acuerdo Parcialmente Aceptar Totalmente

desacuerdo desacuerdo ni desacuerdo de acuerdo de acuerdo

1 2 3 4 5 6 7

e. Preocupación por los efectos secundarios.

Muy en Discrepar Algo en Ni de acuerdo Parcialmente Aceptar Totalmente

desacuerdo desacuerdo ni desacuerdo de acuerdo de acuerdo

1 2 3 4 5 6 7

f. Preocupación por la causa de problemas de salud de la vacuna.

Muy en Discrepar Algo en Ni de acuerdo Parcialmente Aceptar Totalmente

desacuerdo desacuerdo ni desacuerdo de acuerdo de acuerdo

1 2 3 4 5 6 7

g. Preocupación de que la vacuna sea venenosa.

Muy en Discrepar Algo en Ni de acuerdo Parcialmente Aceptar Totalmente

desacuerdo desacuerdo ni desacuerdo de acuerdo de acuerdo

1 2 3 4 5 6 7

h. Creencias negativas sobre la vacuna.

Muy en Discrepar Algo en Ni de acuerdo Parcialmente Aceptar Totalmente

desacuerdo desacuerdo ni desacuerdo de acuerdo de acuerdo

1 2 3 4 5 6 7

i. Falta de información sobre la vacuna.

Muy en Discrepar Algo en Ni de acuerdo Parcialmente Aceptar Totalmente

desacuerdo desacuerdo ni desacuerdo de acuerdo de acuerdo

1 2 3 4 5 6 7

**Risk Perceptions**

Por favor responda a las siguientes afirmaciones. Recordar: cuando hablamos de la **'vacuna contra la COVID-19'**, nos referimos a 1 dosis de cualquier vacuna contra la COVID-19 (Pfizer-BioNTech, Moderna, o Novavax) que se actualizó en 2023-2024.

1. Sería riesgoso para mí recibir la vacuna contra el COVID-19.

Muy en Discrepar Algo en Ni de acuerdo Parcialmente Aceptar Totalmente

desacuerdo desacuerdo ni desacuerdo de acuerdo de acuerdo

1 2 3 4 5 6 7

2. Si recibiera la vacuna contra el COVID-19, habría riesgos.

Muy en Discrepar Algo en Ni de acuerdo Parcialmente Aceptar Totalmente

desacuerdo desacuerdo ni desacuerdo de acuerdo de acuerdo

1 2 3 4 5 6 7

**Vaccine Hesitancy**

Por favor responda a cada una de las siguientes afirmaciones.

1. Me describiría como:

Antivacunas para Ansiosos por recibir la

el COVID-19 vacuna contra el COVID-19

1 2 3 4 5

2. Recibir la vacuna COVID-19 es:

Realmente no Realmente

es importante importante

1 2 3 4 5

3. En general, ¿qué tan reacio está a recibir la vacuna contra el COVID-19?

De nada Mucho

1 2 3 4 5

**Knowledge About Vaccines**

Por favor responda a las siguientes afirmaciones.

1. Sin programas de vacunación ampliamente aplicados, la viruela seguiría existiendo.

Sí No

1 2

2. La eficacia de las vacunas ha sido probada.

Sí No

1 2

3. Los niños serían más resistentes si no estuvieran siempre vacunados contra todas las enfermedades.

Sí No

1 2

4. Enfermedades como el autismo, la esclerosis múltiple y la diabetes pueden desencadenarse mediante vacunas.

Sí No

1 2

5. El sistema inmunológico de los niños no se sobrecarga con muchas vacunas.

Sí No

1 2

6. Muchas vacunas se administran demasiado pronto, de modo que el propio sistema inmunológico del cuerpo no tiene posibilidad de desarrollarse.

Sí No

1 2

7. Las dosis de los químicos utilizados en las vacunas no son peligrosas para los humanos.

Sí No

1 2

8. Las vacunas aumentan la aparición de alergias.

Sí No

1 2

**Vaccine Confidence**

Por favor responda a las siguientes afirmaciones.

1. Estoy completamente seguro de que las vacunas son seguras.

Muy en Algo en Ni de acuerdo Parcialmente Totalmente

desacuerdo desacuerdo ni desacuerdo de acuerdo de acuerdo

1 2 3 4 5

2. La vacunación es innecesaria porque las enfermedades prevenibles con vacunas ya no son comunes.

Muy en Algo en Ni de acuerdo Parcialmente Totalmente

desacuerdo desacuerdo ni desacuerdo de acuerdo de acuerdo

1 2 3 4 5

3. El estrés diario me impide vacunarme.

Muy en Algo en Ni de acuerdo Parcialmente Totalmente

desacuerdo desacuerdo ni desacuerdo de acuerdo de acuerdo

1 2 3 4 5

4. Cuando pienso en vacunarme, sopeso los beneficios y los riesgos para tomar la mejor decisión posible.

Muy en Algo en Ni de acuerdo Parcialmente Totalmente

desacuerdo desacuerdo ni desacuerdo de acuerdo de acuerdo

1 2 3 4 5

5. Cuando todos estén vacunados, yo no tendré que vacunarme también.

Muy en Algo en Ni de acuerdo Parcialmente Totalmente

desacuerdo desacuerdo ni desacuerdo de acuerdo de acuerdo

1 2 3 4 5

**Conspiracy Beliefs**

Por favor responda a las siguientes afirmaciones.

1. El COVID-19 se presenta intencionalmente como peligroso para engañar al público.

Muy en Discrepar Algo en Ni de acuerdo Parcialmente Aceptar Totalmente

desacuerdo desacuerdo ni desacuerdo de acuerdo de acuerdo

1 2 3 4 5 6 7

2. Los expertos nos engañan intencionalmente para su propio beneficio, a pesar de que el COVID-19 no es peor que la gripe.

Muy en Discrepar Algo en Ni de acuerdo Parcialmente Aceptar Totalmente

desacuerdo desacuerdo ni desacuerdo de acuerdo de acuerdo

1 2 3 4 5 6 7

3. Deberíamos creer a los expertos cuando dicen que el COVID-19 es peligroso.

Muy en Discrepar Algo en Ni de acuerdo Parcialmente Aceptar Totalmente

desacuerdo desacuerdo ni desacuerdo de acuerdo de acuerdo

1 2 3 4 5 6 7

4. Las fuerzas oscuras quieren utilizar el COVID-19 para gobernar el mundo.

Muy en Discrepar Algo en Ni de acuerdo Parcialmente Aceptar Totalmente

desacuerdo desacuerdo ni desacuerdo de acuerdo de acuerdo

1 2 3 4 5 6 7

5. El COVID-19 fue traído al mundo intencionalmente para reducir la población.

Muy en Discrepar Algo en Ni de acuerdo Parcialmente Aceptar Totalmente

desacuerdo desacuerdo ni desacuerdo de acuerdo de acuerdo

1 2 3 4 5 6 7

6. Creo que es una tontería que el virus haya sido creado en un laboratorio.

Muy en Discrepar Algo en Ni de acuerdo Parcialmente Aceptar Totalmente

desacuerdo desacuerdo ni desacuerdo de acuerdo de acuerdo

1 2 3 4 5 6 7

7. En general, describiría mi actitud hacia la vacuna contra el COVID-19 como:

En contra Muy entusiasta

1 2 3 4 5

Appendix D

*Example Outline of Semi-Structured Interview Protocols for Phase 4 (Step 6)*

**English**

# **Focus Group 3 Protocol**

**Pre-Interview**

- "Welcome, thanks for coming in. I will explain the focus and purpose of today’s research study to the group soon. But before we can start, we have a few formalities. Could I ask you to read this form and brief survey, and, if you agree, please complete the consent form and return it to me. If you have any questions at all about this study please feel free to ask me now.”
- *Present participants with consent form and demographic survey*.
- *Participants provide written consent and completed demographic survey, collected by researcher/facilitator.*

# **Introduction**

- *Promotor introduces themself and their affiliation.*

“Welcome again, I would like to thank you for agreeing to participate in this focus group discussion for our research study. My name is [NAME] and my role is to facilitate this focus group discussion by asking questions and, if needed, asking for clarification or further information. My role is to also guide the conversation so we do not deviate from the topic of discussion.”

- "Participating in this study today will take about 1 hour in total.”
- “This is a ***focus group*** discussion, this means that I will ask the group questions, and anyone can volunteer to give an answer. It is meant to be a discussion, and all of you can contribute and give your responses. Also, not everyone has to agree with the views and opinions expressed, and there may be some differences in opinion, and this is completely natural, we encourage everyone to express their own views and perspectives on the ideas we discuss. There are no specific rules about giving your responses, you can just indicate that you are about to talk and go ahead and contribute. We just ask you all to please respect other people when they are talking and try not to interrupt. It is also important that if you disagree, please do so in a respectful, friendly way *<<smile>>*.”
- “In this focus group discussion, we are interested in exploring your views of the COVID-19 vaccines. So, I will ask the group a number of questions about some COVID-19 messages, and I expect those questions will be a starting point for the discussion. There are NO right or wrong answers so please respond as honestly as you can. We really value your opinion on this topic and so please feel free to share your opinions as openly as possible. I expect to spend about an hour discussing this issue, but there is no specific fixed time.”
- “Just a reminder, although you will have seen it on the form I just gave you, it is important to remember that we will not write down any information today that can identify you, and all the information and opinions we gather during this focus group will be recorded in such a way that no individual participant can be identified. So, please remember not to state your name or other people’s names during our discussion.”
- “The focus group session is being audio-recorded. Please try to speak as clearly as you can to help with the audio recording. This will allow me to focus on what you are saying and not rely on my memory. The audio tapes will be securely stored and only accessible to the researchers. When the study is completed, all audio recordings will be destroyed.”

# **Icebreaker activity**

- “To begin the discussion, I’m wondering if you would like to share a little about your hobbies or things you like to do?”

# **COVID Understanding Exercise**

- I’d also like to learn what you all think about COVID-19. What do you think it is?
- How do you think COVID-19 spreads from person to person?

# **Main Interview (Group Questions)**

- **“**The specific topic of today’s focus group will be a discussion about your views and opinions on a series of COVID-19 vaccine messages. **These original messages were presented to people like you, and from their valuable feedback we redesigned them.**
- We are particularly interested in how well you think the **resigned message text** will be understood by people from this community in <<LOCATION>>, whether people and people like you will understand them, and whether they are clear and well-put or phrased, or whether **they** could be **further** improved. As a reminder, all information that you give in this focus group will be kept anonymous, so please do not state your name or anyone else’s name during this focus group.”
- “So I am now going read a series of passages to you which make up the **original** COVID-19 vaccination messages, **and then I will present the suggested text for the redesign of the message.** As you can see I have also projected the messages on the screen. Please listen to the messages, and read along if you like. All the while, please think about whether you think the **redesigned message text** would be effective in promoting you to get vaccinated, and whether they would be useful or effective for people in your community. Also, think about whether you think the **resigned message** could be **further** improved, or whether there are other things that could be added or changed to improve understanding. Also, please also let me know if there is something you would like me to repeat”.

# **Message review: Appropriateness & Feasibility**


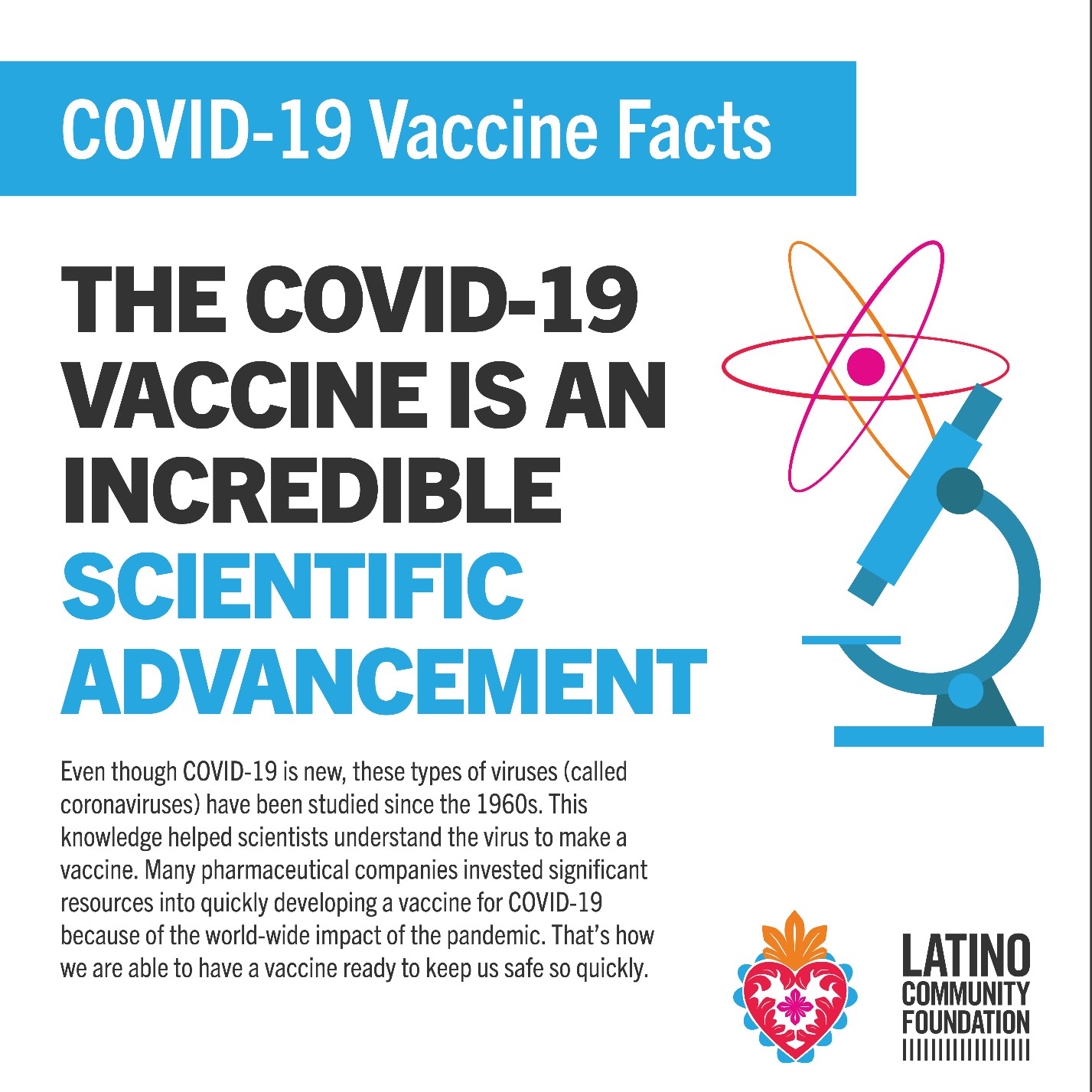


Message 1 Revised Message:

THE COVID -19 VACCINE IS AN INCREDIBLE SCIENTIFIC ADVANCEMENT

Even though COVID-19 is a new virus, it is the same kind of virus that has been studied by scientists for many years since the 1960s. This knowledge helped scientists understand the virus and helped them make a COVID-19 vaccine. Due to the world-wide impact of the pandemic, pharmaceutical companies worked together and invested significant resources to quickly develop a vaccine for COVID-19. Even though it was developed rapidly, safety was always the most important concern and the vaccine was subject to strict safety testing to make sure it was safe before it was launched. That’s how we are able to have a vaccine ready to help protect everyone from the virus so quickly but also safely.

Latino Community Foundation

Where to get more information: Latino Community Foundation, website provided.

- - What is your first reaction or initial thoughts when you **read the redesigned message text?** [Do you think this message would work for you; for example, would you be persuaded to get vaccinated?] [Why or why not?]
  - Do you think the **redesigned message text** would be effective for the people in the community you interact and live with? [How so?] [How could it be **further** improved?]


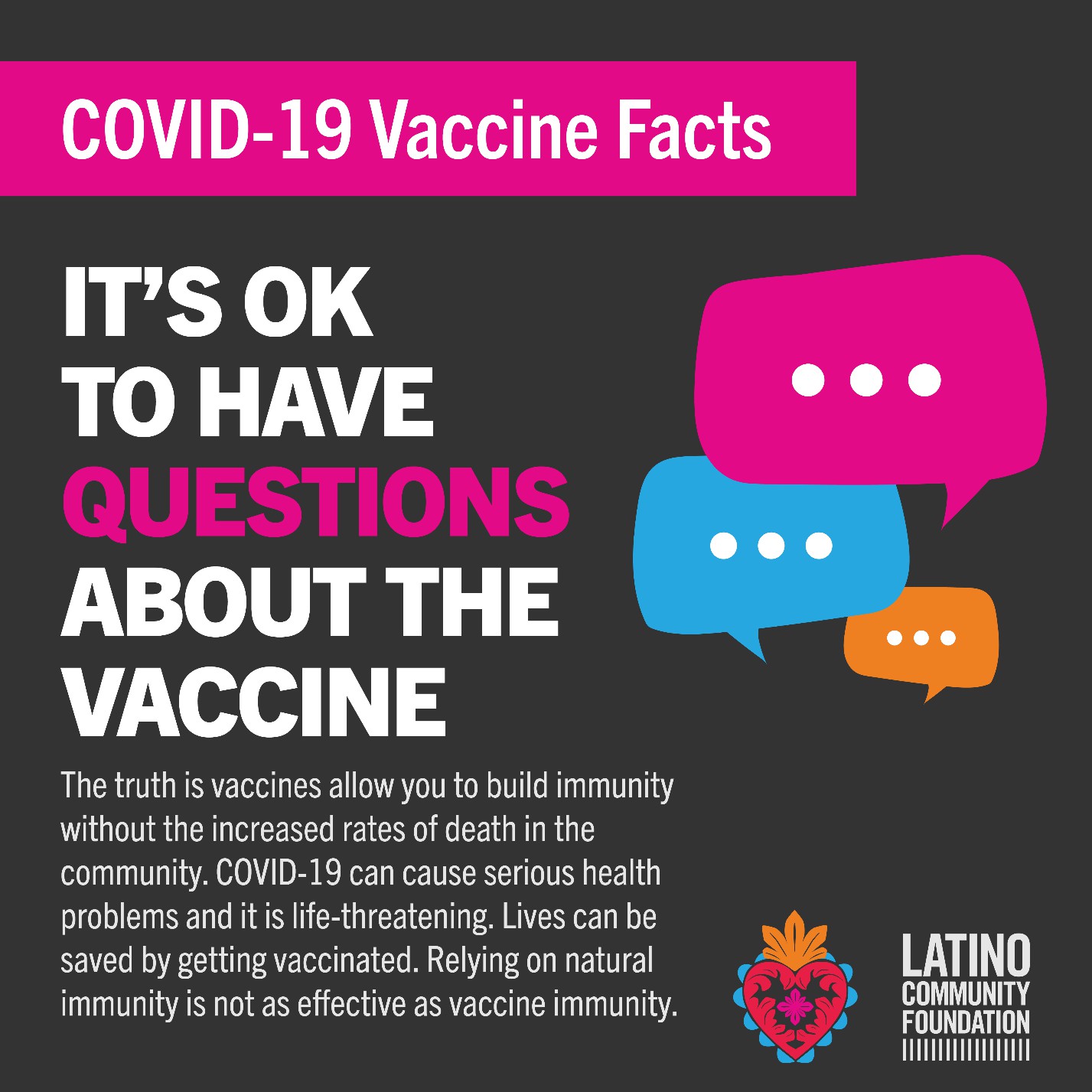


Message 2 Revised Message:

COVID-19 Vaccine Facts

IT’S OK TO HAVE CONCERNS OR QUESTIONS ABOUT THE COVID-19 VACCINE

COVID-19 can cause serious health problems and is life-threatening. Serious cases of the virus can be prevented and lives can be saved by getting vaccinated. The COVID-19 vaccine has been developed to help you and others build immunity without increased rates of serious illness or death in the community. This is especially important for those who are at very high risk, such as those with chronic conditions and the elderly. Relying on natural immunity is not as effective as vaccine immunity.

Where to get more information: Latino Community Foundation, website provided.

- - What is your first reaction or initial thoughts when you **read the redesigned message text?** [Do you think this message would work for you; for example, would you be persuaded to get vaccinated?] [Why or why not?]
  - Do you think the **redesigned message text** would be effective for the people in the community you interact and live with? [How so?] [How could it be **further** improved?]


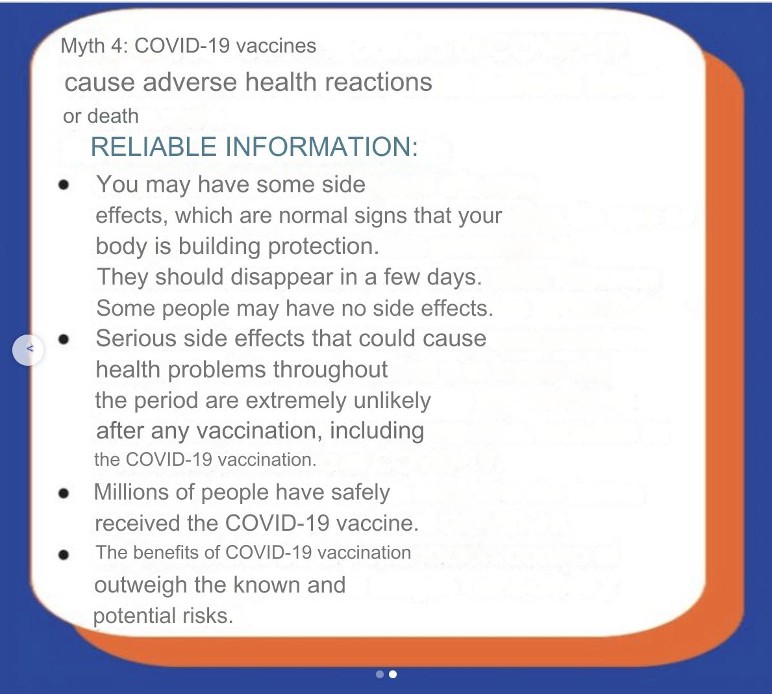


Message 3 Revised Message:

The COVID-19 vaccine has been developed by scientists and safety was the top priority. Even though it was developed rapidly, it was thoroughly tested for safety before it was made available.

Millions of people have safely received the COVID-19 vaccine.

There is a need to get an up-to-date vaccine to maintain your immunity to protect yourself and others. It is not true that the vaccine causes adverse health reactions or death or gives you COVID-19.

If you get the vaccine, you may have some side effects. This is completely normal and safe, it is just a sign that you body is building protection against the virus. Any side effects will disappear in a few days.

Some people have no side effects.

The benefits of COVID-19 vaccination outweigh any known risks. Serious side effects that could cause health problems are extremely unlikely.

- - What is your first reaction or initial thoughts when you **read the redesigned message text?** [Do you think this message would work for you; for example, would you be persuaded to get vaccinated?] [Why or why not?]
  - Do you think the **redesigned message text** would be effective for the people in the community you interact and live with? [How so?] [How could it be **further** improved?]


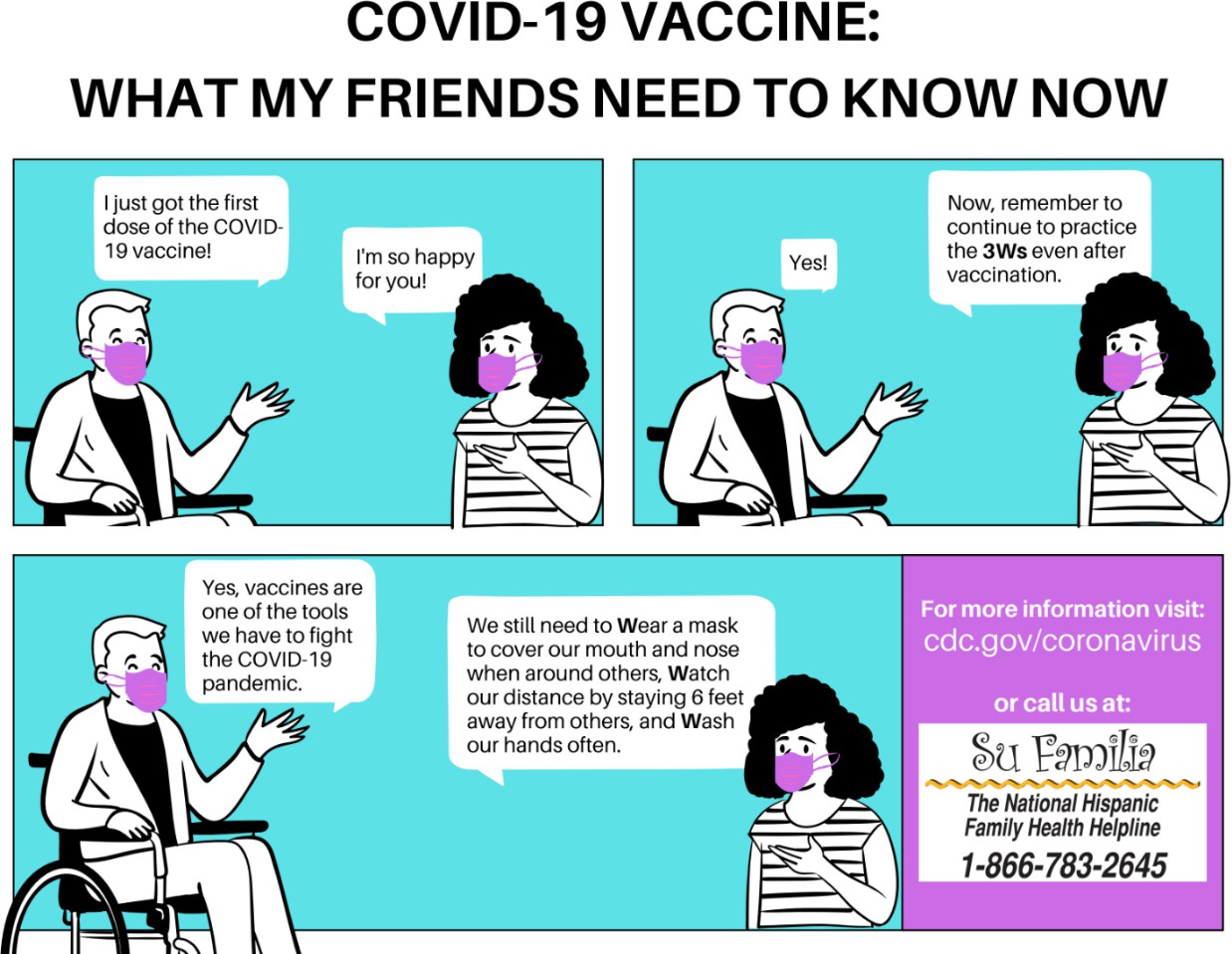


Message 4 Revised Message:

Picture 1

Man: I just got my first dose of the COVID-19 vaccine! Woman: I’m so happy for you! It’s really important that you keep up-to-date with your COVID-19 vaccine to maintain your immunity to protect yourself and others. Picture 2

Woman: Now it’s important to remember to still continue to practice the “3Ws” in certain situations, even if you have been vaccinated. For example if you have symptoms or if you are around people who are at high risk like those with chronic conditions or the elderly.

Man: Yes!

Picture 3

Woman: Remember, the three **W**s? **W**ear a mask to cover our nose and mouth when around others, **W**atch your distance by staying 6 feet away from others, and **W**ash your hands often.

Man: Yes, vaccines is the best tool we have to continue to keep the COVID-19 under control, but it is also important to remember the 3Ws, particularly around those people who are vulnerable.

For more information and to find your nearest vaccination location and transport links visit: website

- - What is your first reaction or initial thoughts when you **read the redesigned message text?** [Do you think this message would work for you; for example, would you be persuaded to get vaccinated?] [Why or why not?]
  - Do you think the **redesigned message text** would be effective for the people in the community you interact and live with? [How so?] [How could it be **further** improved?]


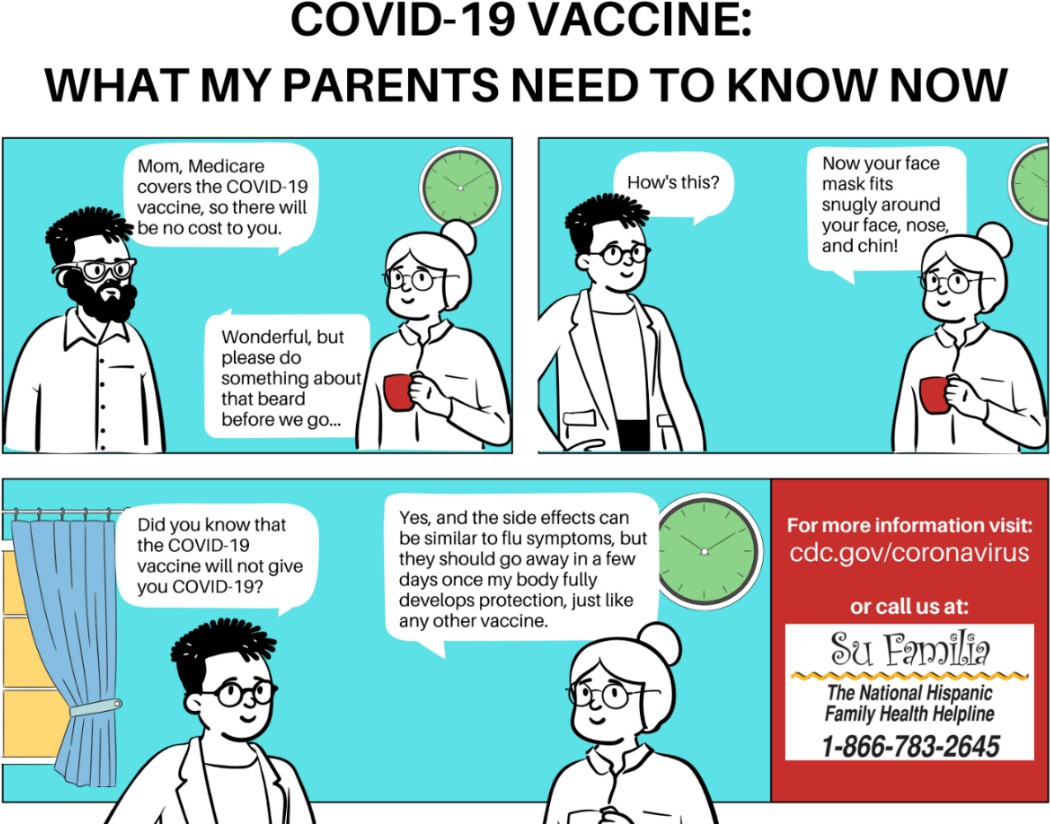


Message 5 Revised Message:

Picture 1

Son: Mom, at your age it’s really important that you keep up-to-date with your COVID-19 vaccine to protect yourself and your family and loved ones. And Medicare covers the COVID-19 vaccine, so there will be no cost to you!

Mother: Wonderful, and thanks for mentioning my age! But seriously, people who are older and those with chronic conditions are most at risk, so it is important for you to get vaccinated as well and everyone else! Now, are you going to do something about that beard before we go and get vaccinated…? Picture 2

Son: How’s this?

Mother: Not bad. It also means that if you need to wear a mask to protect yourself and the people close to you from COVID-19, it will fit snugly around your face and chin!

Picture 3

Son: By the way, did you know that the COVID-19 vaccine will not actually give you COVID-19?

Mother: Yes, I have been reading about that! And even though there might be flu like side effects, they will go away in a few days once your body fully develops protection, just like any other vaccine. I also read that even though the vaccine was developed very quickly, it was thoroughly tested for safety, so I am confident that it is safe.

For more information and to find your nearest vaccination location and transport links visit: website

- - What is your first reaction or initial thoughts when you **read the redesigned message text?** [Do you think this message would work for you; for example, would you be persuaded to get vaccinated?] [Why or why not?]
  - Do you think the **redesigned message text** would be effective for the people in the community you interact and live with? [How so?] [How could it be **further** improved?]


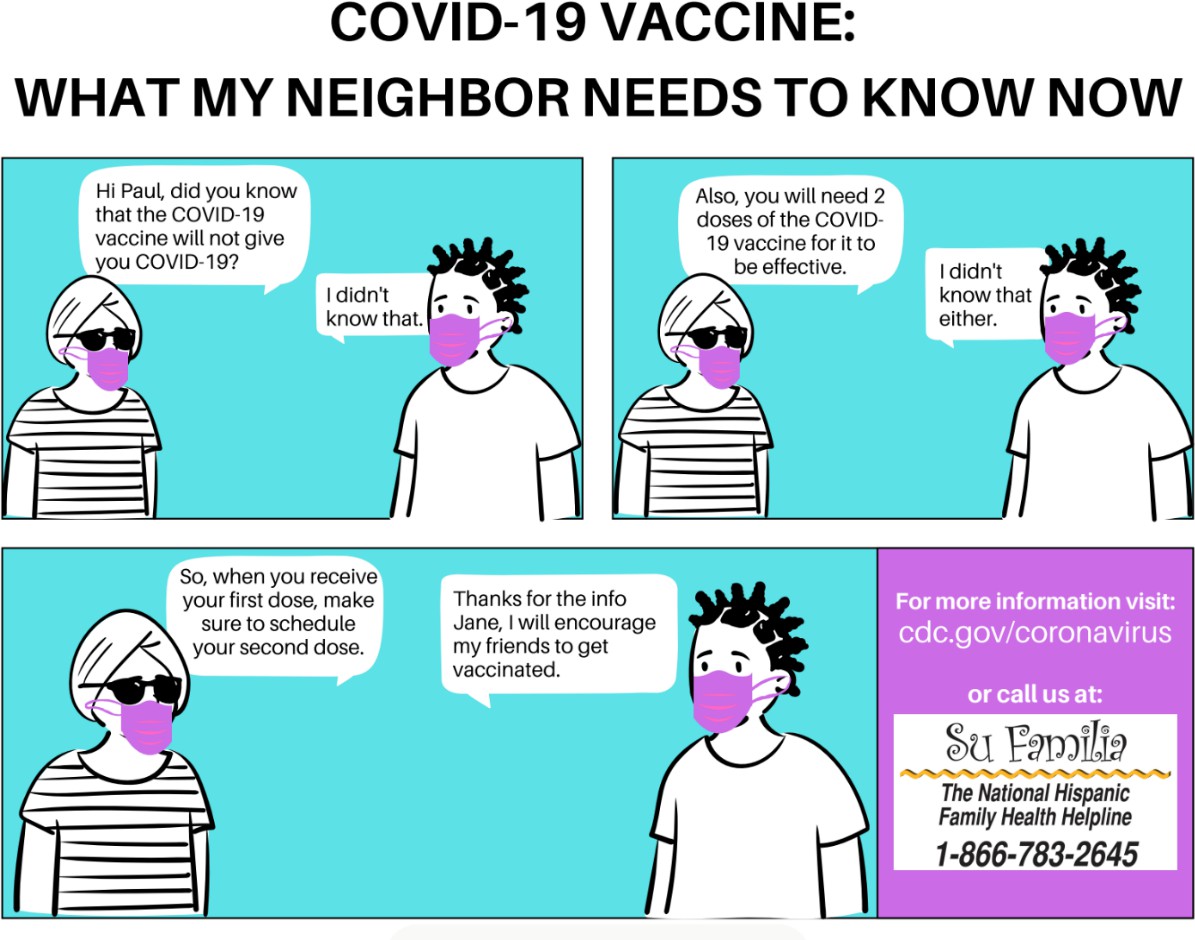


Message 6 Revised Message:

Picture 1

Jane: Hi Paul, did you know than the COVID-19 vaccine will not give you COVID-19?

Paul: I didn’t know that. I am just worried about its safety.

Jane: It’s very safe. Even though the vaccine was developed by scientists very quickly, it was stringently tested for safety, so you can be certain it’s very safe. Paul: That’s good to know!

Picture 2

Jane: Also, it’s important that you keep up-to-date with your vaccine to maintain your immunity to protect yourself and others.

Paul: I didn’t know that either. Picture 3

Jane: So, even if you have had the COVID-19 vaccine in the past, because the virus keep changing you still need to get the updated one.

Paul: Thanks for the info Jane, I will encourage my friends to get vaccinated.

For more information and to find your nearest vaccination location and transport links visit: website

- - What is your first reaction or initial thoughts when you **read the redesigned message text?** [Do you think this message would work for you; for example, would you be persuaded to get vaccinated?] [Why or why not?]
  - Do you think the **redesigned message text** would be effective for the people in the community you interact and live with? [How so?] [How could it be **further** improved?]

# **Post-Interview**

- “Thank you very much for sharing your honest thoughts and opinions related to the COVID-19 vaccine. This concludes our focus group discussion.”
- “I will give you your gift card for participating in our research study today. Thank you very much.”

**Spanish**

# **Protocolo del grupo focal 3**

**Antes del Grupo Focal**

- "Bienvenidos, gracias por venir. En un momento, les explicaré el enfoque y el propósito del estudio de investigación de hoy. Pero antes de que podamos empezar, tenemos algunas formalidades. ¿Podría pedirles que lean esta forma y encuesta y, si están de acuerdo, por favor completen la forma de consentimiento y entréguenmelo. Si tienen alguna pregunta sobre este estudio, no duden en preguntarme ahora."
- *Entregue la forma de consentimiento y la encuesta de información demográfica a los participantes*
- *Los partcipantes entreguen la forma de consentimiento y la encuesta de información demográfica completa a los facilitadores*

# **Introducción**

- *Los facilitadores se presentan y tambien presentan a su afiliación*

“Bienvenido nuevamente, me gustaría agradecerle por aceptar participar en esta discusión de grupo focal para nuestro estudio de investigación. Mi nombre es [NOMBRE] y mi función es facilitar esta discusión de grupo focal haciendo preguntas y, si es necesario, solicitando aclaraciones o más información. Mi función también es guiar la conversación para que no nos desviemos del tema de discusión.”

- "Participación en este estudio de hoy tomará aproximadamente 1 hora en total.”
- "Es un discusión ***de grupo de enfoque***, esto significa que haré preguntas al grupo y quien quiera puede ofrecerse como voluntario para dar una respuesta. Está destinado a ser una discusión y todos ustedes pueden contribuir y dar sus respuestas. Además, no todos tienen que estar de acuerdo con los puntos de vista y opiniones expresados, y puede haber algunas diferencias de opinión, y esto es completamente natural; alentamos a todos a expresar sus propios puntos de vista y perspectivas sobre las ideas que discutimos. No existen reglas específicas para dar tus respuestas, simplemente puedes indicar que estás a punto de hablar y seguir adelante y contribuir. Sólo les pedimos a todos que por favor respeten a todos los demás cuando hablan y traten de no interrumpir. También es importante que si no está de acuerdo, lo haga de manera respetuosa y amigable.*<<sonrisa>>*.”
- “En esta discusión de grupo focal, estamos interesados en explorar sus experiencias con las vacunas sobre el COVID-19. Entonces, le haré al grupo una serie de preguntas sobre experiencias relacionadas con las vacunas sobre el COVID-19, y espero que esas preguntas sean un punto de partida para la discusión. NO hay respuestas correctas o incorrectas, así que responda lo más honestamenste posible. Realmente valoramos su opinión sobre este tema y, por lo tanto, siéntase libre de compartir sus experiencias de la manera más abierta posible. Espero dedicar aproximadamente una hora a discutir este tema, pero no hay un tiempo fijo específico”.
- “Solo un recordatorio, aunque habrá visto en el formulario que le acabo de dar, es importante recordar que hoy no escribiremos ninguna información que pueda identificarlo, ni toda la información y opiniones que recojamos durante este grupo focal. Se registrarán de tal manera que no se pueda identificar a ningún participante individual. Por lo tanto, recuerde no decir su nombre ni el de otras personas durante nuestra conversación.”
- “La sesión del grupo focal se está grabando en audio. Intente hablar lo más claramente posible para ayudar con la grabación de audio. Esto me permitirá concentrarme en lo que estás diciendo y no confiar en mi memoria. Las cintas de audio se almacenarán de forma segura y solo serán accesibles para los investigadores. Cuando se complete el estudio, todas las grabaciones de audio serán destruidas”.

# **Rompehielo**

- “Para comenzar la discusión, pregunto si le gustaría compartir un poco sobre sus pasatiempos o las cosas que le gusta hacer.”

# **Ejercicio de comprensión de COVID**

- También me gustaría saber qué piensan todos ustedes sobre el COVID-19.

¿Qué creen que es?

- ¿Cómo creen que se transmite el COVID-19 de persona a persona?

# **Entrevista Principal (preguntas para el grupo)**

- **“**El tema específico del grupo focal de hoy será una discusión sobre sus puntos de vista y opiniones sobre una serie de mensajes sobre la vacuna COVID-19. **Los mensajes originales se presentaron a personas como ustedes y, con sus valiosos comentarios los rediseñamos.**
- Estamos particularmente interesados en saber qué tan bien crees que **el texto de los mensajes rediseñados sería** entendido por las personas de esta comunidad en Madera, si personas como usted los entenderán, y si son claros y bien expresados o redactados, o si se podrían mejorar **más.** Como recordatorio, toda la información que proporcione en este grupo de enfoque se mantendrá anónima, así que no diga su nombre ni el de ninguna otra persona durante este grupo de enfoque”.
- “Así que ahora les leeré una serie de pasajes que componen los mensajes **originales** de vacunación **contra el COVID-19 y luego presentaré el texto sugerido para el rediseño del mensaje**. También he proyectado los mensajes en la pantalla. Escuche los mensajes y léalos si lo desea. Mientras, piense si cree que **los mensajes rediseñados** serían eficaces para promover que usted se vacune y si serían útiles o eficaces para las personas de su comunidad. Además, piense si cree que **el texto del mensaje rediseñado** se podría mejorar **más** o si hay otras cosas que podrían agregarse o cambiarse para mejorar la comprensión. Además, por favor déjame saber si hay algo que le gustaría que repitiera”.

**Revisión de los mensajes: La Idoneidad y Viabilidad**


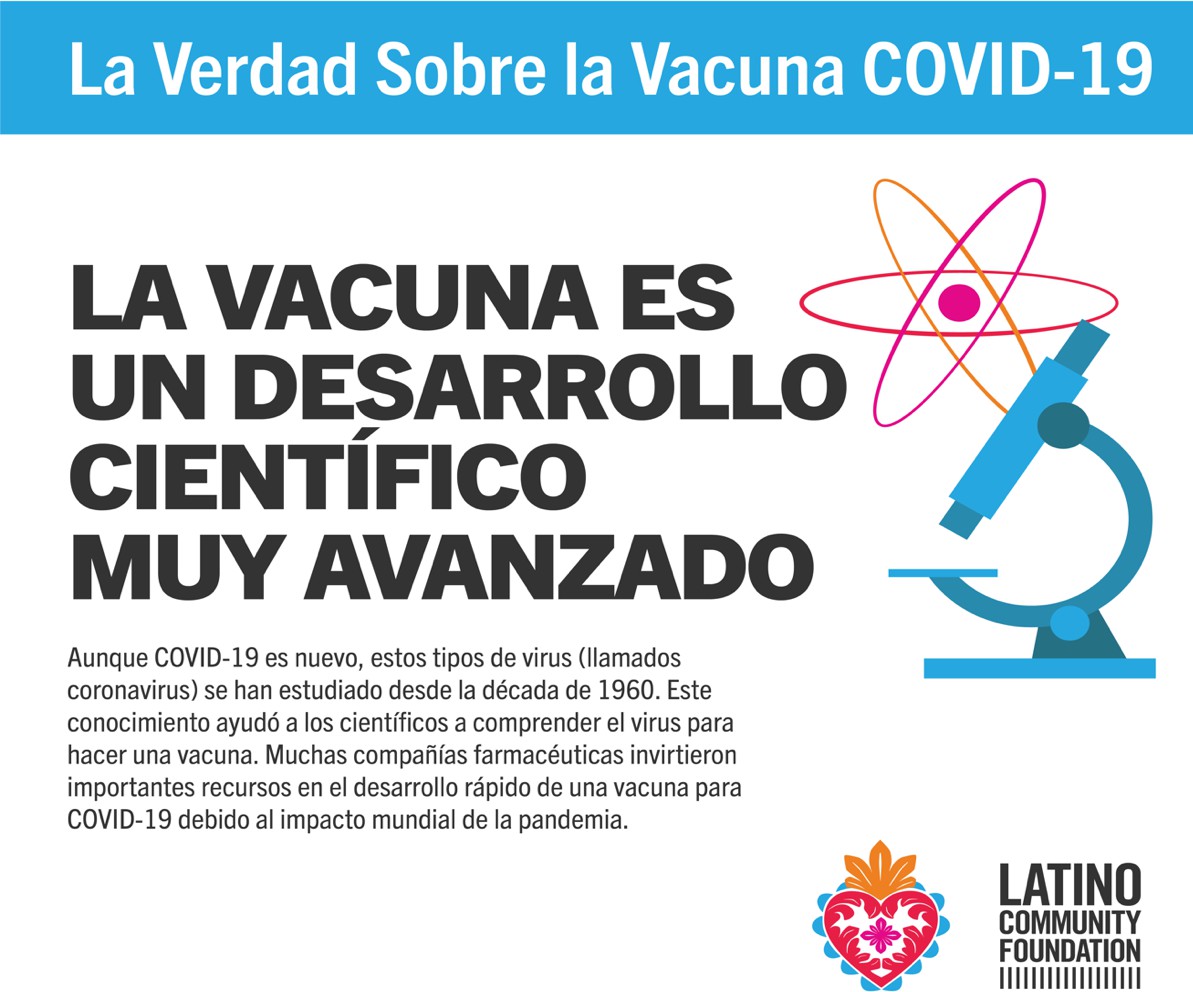


**Revisión del mensaje 1:**

LA VACUNA ES UN DESARROLLO CIENTÍFICO MUY AVANZADO

Aunque COVID-19 es un virus nuevo, es el mismo tipo de virus que los científicos han estado estudiando durante muchos años desde la década de 1960. Este conocimiento ayudó a los científicos a comprender el virus y les ayudó a hacer una vacuna contra el COVID-

19. Debido al impacto mundial de la pandemia, las compañías farmacéuticas trabajaron juntas e invirtieron importantes recursos para desarrollar rápidamente una vacuna contra el COVID-19. Aunque se desarrolló rápidamente, la seguridad siempre fue la preocupación más importante y la vacuna se sometió a estrictas pruebas de seguridad para asegurarse de que fuera segura antes de su lanzamiento. Así es como podemos tener una vacuna lista para ayudar a proteger a todos del virus de manera tan rápida pero también segura.

Latino Community Foundation

Dónde obtener más información: Latino Community Foundation, sitio web proporcionado.


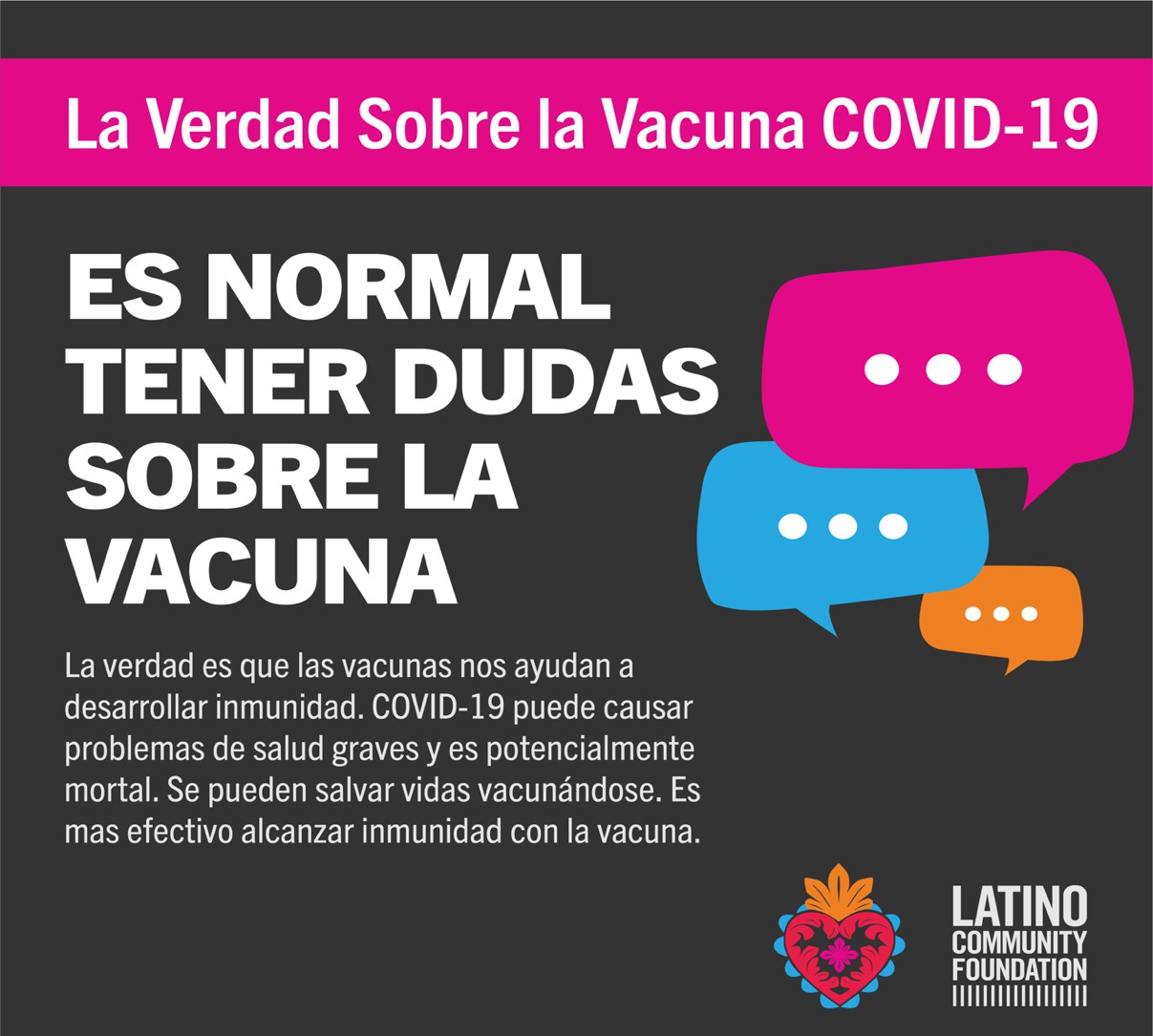


**Revisión del mensaje 2:**

La Verdad Sobre la Vacuna COVID-19

ES NORMAL TENER PREOCUPACIONES O PREGUNTAS SOBRE LA VACUNA CONTRA EL COVID-19

COVID-19 puede causar problemas de salud graves y es potencialmente mortal. Los casos graves del virus se pueden prevenir y de pueden salvar vidas vacunándose. La vacuna contra el COVID-19 se ha desarrollado para ayudar a usted y a otras personas a desarrollar inmunidad sin aumentar las tasas de enfermedad grave o muerte en la comunidad. Esto es especialmente importante para aquellos que tienen un riesgo muy alto, como las personas con afecciones crónicas y la población anciana. Es más efectivo alcanzar inmunidad con la vacuna.

Dónde obtener más información: Latino Community Foundation, sitio web proporcionado.


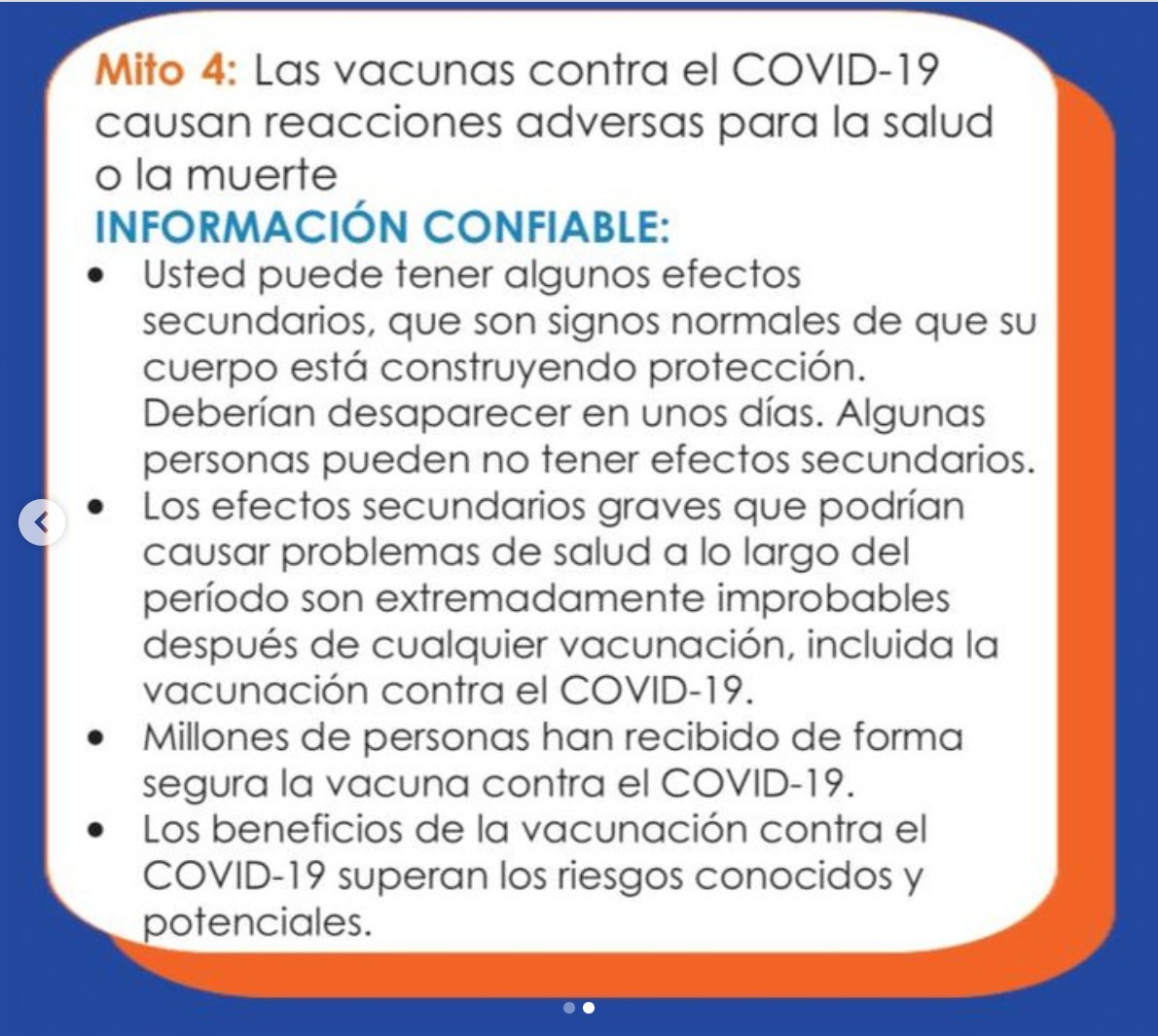


**Revisión del mensaje 3:**

La vacuna contra el COVID-19 ha sido desarrollada por científicos y la seguridad era la máxima prioridad. Aunque se desarrolló rápidamente, se probó minuciosamente su seguridad antes de que estuviera disponible.

Millones de personas han recibido de forma segura la vacuna contra el COVID-19.

Es necesario recibir la vacuna más reciente para mantener su inmunidad y protegerse a sí mismo y a los demás.

No es cierto que la vacuna cause reacciones adversas a la salud o la muerte o que le dé COVID-19.

Si usted recibe la vacuna, puede tener algunos efectos secundarios. Esto es completamente normal y seguro, y es un signo que su cuerpo está construyendo protección contra el virus. Deberían desparecer en unos días. Algunas personas pueden no tener efectos secundarios.

Los beneficios de la vacunación contra el COVID-19 superan los riesgos conocidos y potenciales. Los efectos secundarios graves que podrían causar problemas de salud son extremadamente improbables.


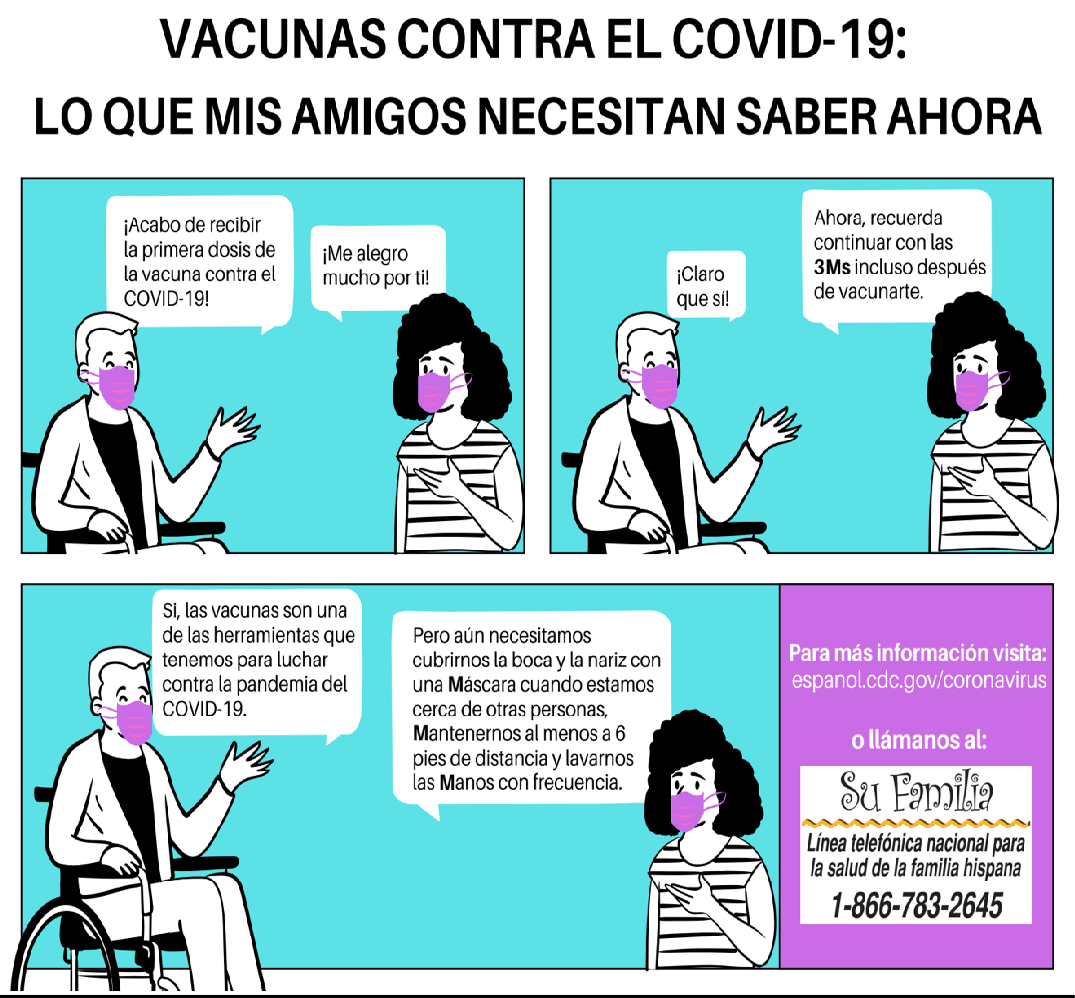


**Revisión del mensaje 4:**

**Imagen 1 (recuadro 1)**

El hombre: ¡Acabo de recibir la primera dosis de la vacuna contra el COVID-19!

La mujer: ¡Me alegro mucho por usted! Es muy importante que continúe recibiendo la vacuna contra el COVID-19 más reciente para mantener su inmunidad y protegerse a sí mismo y a los demás.

**Imagen 2 (recuadro 2)**

La mujer: Ahora, es importante recordar seguir practicando las “3Ms” en ciertas situaciones, incluso si ha sido vacunado. Por ejemplo, si tiene síntomas o si está cerca de personas que están en alto riesgo, como las personas con afecciones crónicas o los de la población anciana.

El hombre: ¡Si!

**Imagen 3 (recuadro 3)**

La mujer: ¿Recuerdas las 3Ms? Necesitamos cubrirnos la boca y la nariz con una **M**áscara cuando estamos cerca de otras personas, **M**antenernos al menos a 6 pies de distancia y lavarnos las **M**anos con frecuencia.

El hombre: Si, las vacunas son una de las herramientas que tenemos para luchar contra la pandemia del COVID- 19, pero también es importante recordar las 3M, especialmente cuando estamos cerca de aquellas personas que son vulnerables.

Para obtener más información y encontrar el lugar de vacunación más cercano e información sobre el transporte, visite: sitio web


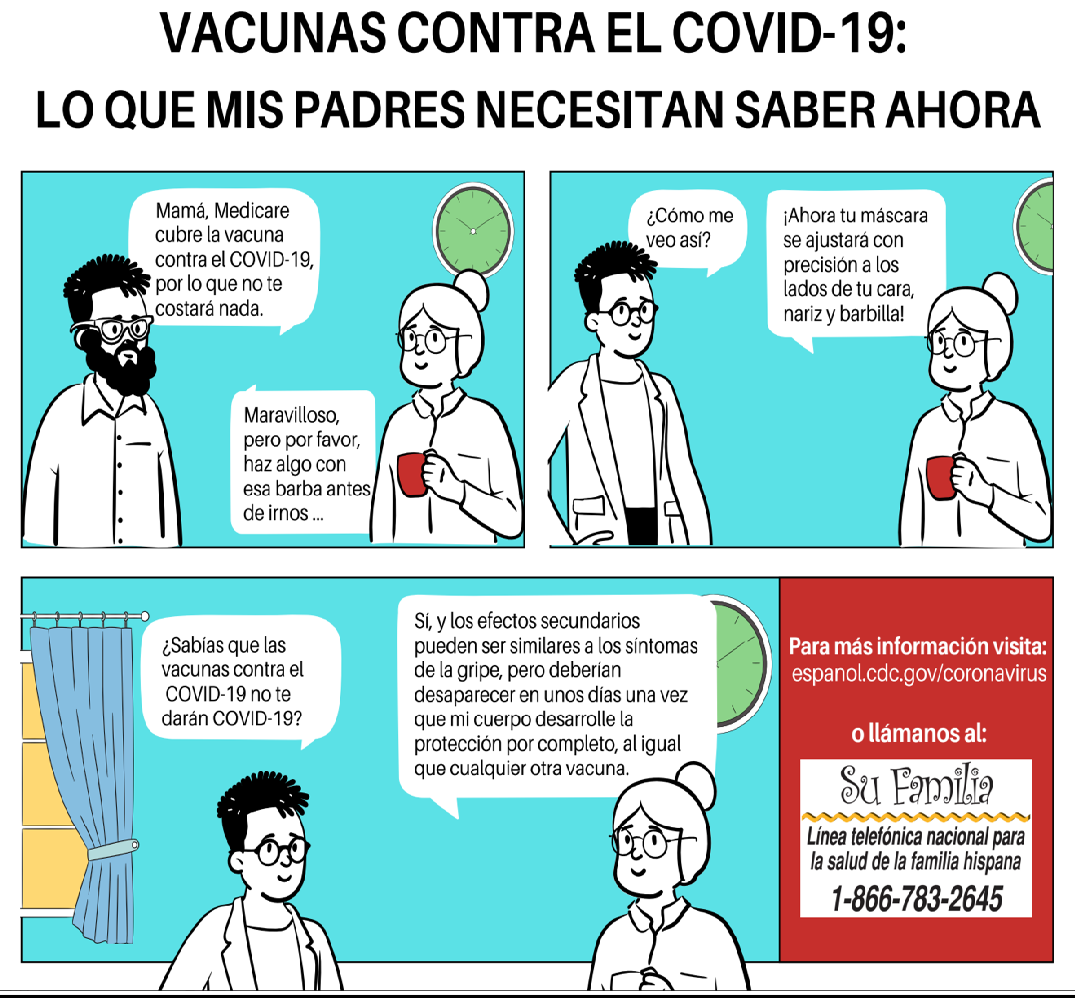


**Revisión del mensaje 5:**

**Imagen 1 (recuadro 1)**

El hijo: Mamá, a su edad es muy importante que sigas recibiendo la vacuna contra el COVID-19 más reciente para protegerte misma y proteger a la familia y sus queridos. Y Medicare cubre la vacuna contra el COVID-19, por lo que no costará nada.

La mamá: ¡Maravilloso, y gracias por mencionar mi edad!

¡Pero en serio, las personas mayores y los que tienen afecciones crónicas corren mayor riesgo, así que es importante que usted también se vacune y que todos los demás se vacunen! Ahora, ¿vas a hacer algo con esa barba antes de que vayamos a vacunarnos...?

**Imagen 2 (recuadro 2)**

El hijo: ¿Cómo me veo así?

La mamá: Bien. También significa que necesitas usar una mascarilla para protegerte a sí mismo y a las personas cercanas a usted del COVID-19, ¡ahora la máscara se ajustará perfectamente alrededor de su cara y barbilla!

**Imagen 3 (recuadro 3)**

El hijo: ¿A propósito, sabias que las vacunas contra el COVID-19 no te darán COVID-19?

La mamá: ¡Sí, he estado leyendo sobre eso! Aunque los efectos secundarios pueden ser similares a los síntomas de la gripe, pero deberían desaparecer en unos pocos días después de que su cuerpo desarrolle la protección por completo, al igual a cualquier otra vacuna. También leí que, aunque la vacuna se desarrolló muy rápidamente, se probó exhaustivamente su seguridad, así que tengo confianza en que es segura la vacuna.

Para obtener más información y encontrar el lugar de vacunación más cercano e información sobre el transporte, visite: sitio web


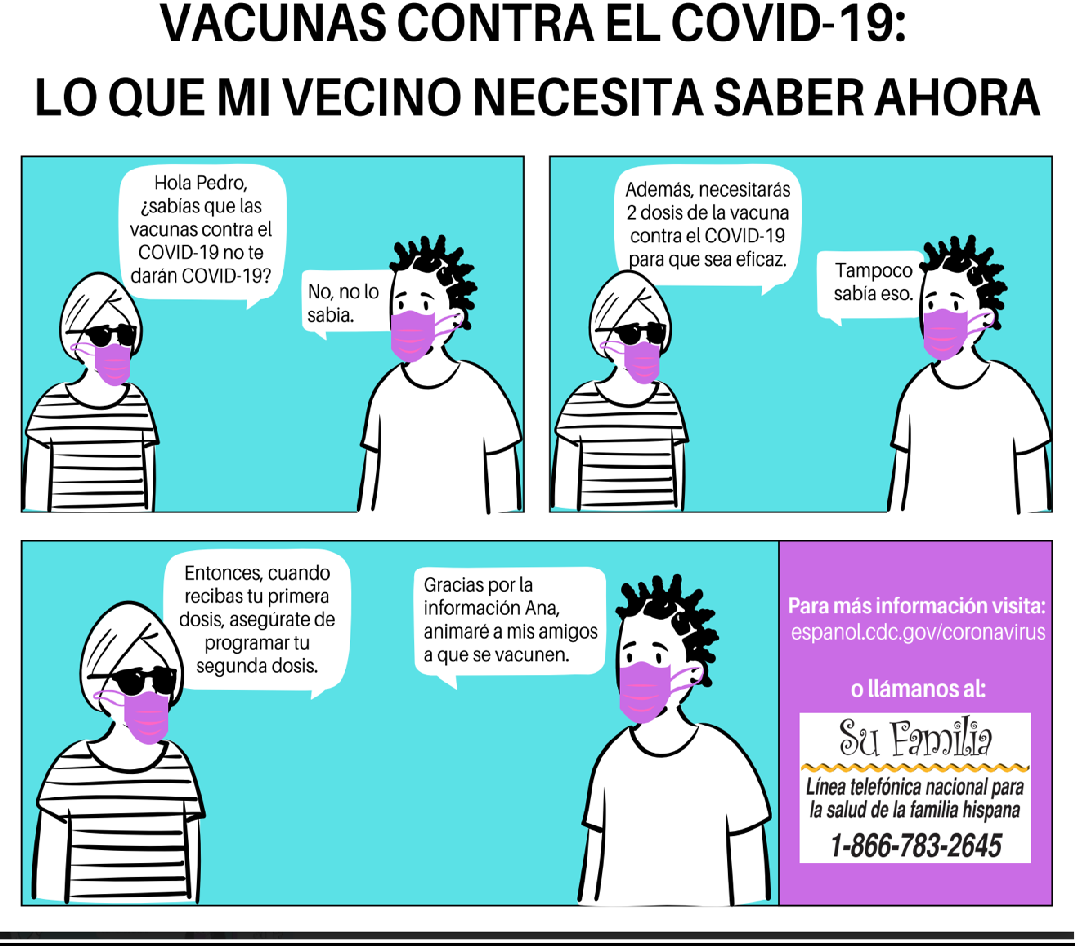


**Revisión del mensaje 6:**

**Imagen 1 (recuadro 1)**

Ana: Hola Pedro, ¿sabías que las vacunas contra el COVID-19 no te darán COVID-19?

Pedro: No, no lo sabía. Solo me preocupa su seguridad. Ana: Es muy seguro. Aunque los científicos desarrollaron la vacuna muy rápidamente, se sometió a pruebas estrictas de seguridad, así que puedes estar seguro de que es muy segura la vacuna.

Pedro: ¡Es bueno saberlo!

**Imagen 2 (recuadro 2)**

Ana: Además, es importante seguir recibiendo la vacuna más reciente para mantener su inmunidad y protegerse a sí mismo y a los demás.

Pedro: Tampoco sabía eso.

**Imagen 3 (recuadro 3)**

Ana: Por lo tanto, incluso si recibió la vacuna contra el COVID-19 en el pasado, aún debe recibir la vacuna más reciente porque el virus sigue cambiando.

Pedro: Gracias por la información Ana, animaré a mis amigos que se vacunen.

Para obtener más información y encontrar el lugar de vacunación más cercano e información sobre el transporte, visite: sitio web

**Después del Grupo Focal**

- “Muchas gracias por compartir sus pensamientos y opiniones honestos

relacionados con la vacuna sobre el COVID-19. Con esto se concluye el grupo

focal”.

- “Le daré su tarjeta de regalo por participar hoy en nuestro estudio de

investigación. Muchas gracias por su participación."
